# Extracellular Polymeric Substances (EPS) of Freshwater Biofilms Stabilize and Modify CeO_2_ and Ag Nanoparticles

**DOI:** 10.1371/journal.pone.0110709

**Published:** 2014-10-21

**Authors:** Alexandra Kroll, Renata Behra, Ralf Kaegi, Laura Sigg

**Affiliations:** 1 Environmental Toxicology, Swiss Federal Institute of Aquatic Science and Technology (Eawag), Dübendorf, Switzerland; 2 Institute of Biogeochemistry and Pollutant Dynamics IBP, Swiss Federal Institute of Technology in Zurich (ETHZ), Zurich, Switzerland; Catalan Institute for Water Research (ICRA), Spain

## Abstract

Streams are potential receiving compartments for engineered nanoparticles (NP). In streams, NP may remain dispersed or settle to the benthic compartment. Both dispersed and settling NP can accumulate in benthic biofilms called periphyton that are essential to stream ecosystems. Periphytic organisms excrete extracellular polymeric substances (EPS) that interact with any material reaching the biofilms. To understand the interaction of NP with periphyton it is therefore crucial to study the interaction of NP with EPS. We investigated the influence of EPS on the physicochemical properties of selected NP (CeO_2_, Ag) under controlled conditions at pH 6, 7.6, 8.6 and light or dark exposure. We extracted EPS from five different periphyton communities, characterized the extracts, and exposed CeO_2_ and carbonate-stabilized Ag NP (0.5 and 5 mg/L, both 25 nm primary particle size) and AgNO_3_ to EPS (10 mg/L) over two weeks. We measured NP size distribution, shape, primary particle size, surface plasmon resonance, and dissolution. All EPS extracts were composed of biopolymers, building blocks of humic substances, low molecular weight (M_r_) acids, and small amphiphilic or neutral compounds in varying concentrations. CeO_2_ NP were stabilized by EPS independent of pH and light/dark while dissolution increased over time in the dark at pH 6. EPS induced a size increase in Ag NP in the light with decreasing pH and the formation of metallic Ag NP from AgNO_3_ at the same conditions via EPS-enhanced photoreduction. NP transformation and formation were slower in the extract with the lowest biopolymer and low M_r_ acid concentrations. Periphytic EPS in combination with naturally varying pH and light/dark conditions influence the properties of the Ag and CeO_2_ NP tested and thus the exposure conditions within biofilms. Our results indicate that periphytic organisms may be exposed to a constantly changing mixture of engineered and naturally formed Ag NP and Ag^+^.

## Introduction

Due to the increased production of engineered nanoparticles (NP), they have become potential environmental contaminants that may be transported in streams. Possible routes of entry have been suggested previously [1] such as spills, treated sewage water, run-off, or rain. Depending on the route of entry, engineered NP may undergo transformation. Sewage treatment for example seems to sulfidize Ag NP depending on their size [2]. Heterogeneity in NP concentration and state of transformation in the environment can thus be expected. Consequently, to be able to estimate the impact of engineered NP on aquatic ecosystems, the interactions of NP with various components of these systems need to be studied.

One potential receiving compartment are biofilms, called periphyton, which colonize submerged surfaces. Periphyton is a taxonomically diverse and dynamic community of photo- and heterotrophic microorganisms, *i.e.* bacteria, algae, and fungi that adapts quickly to environmental changes [3]. It is an essential element of aquatic ecosystems contributing to primary production and release of oxygen [4], influencing the hydrodynamics of the water body [5], and being a habitat for different life stages of uni- and multicellular organisms. Periphyton was shown to be the primary receptor of gold nanorods in a model estuarine system [6] and to accumulate CuO NP when exposed in mesocosms [7]. The accumulation of TiO_2_ NP in freshwater biofilms and mono-species algal biofilms in mesocosm experiments positively correlated with the amount of biomass [8]. Sedimentation of micrometer sized particles is influenced by periphyton due to the change in hydrologic conditions near the biofilms [9]. Whether this applies to nano-sized particles has not been investigated, however, engineered NP may occur in micrometer-size agglomerates depending on NP properties, concentration, water chemistry, presence of other particles or colloids, hydrological conditions, solar radiation, and temperature. Relevant NP properties are size and surface charge, which are in turn influenced by NP surface modifications and solubility, as well as ionic strength, presence of bivalent cations, organic matter, and pH [10], [11], [12], [13], [14], [15]. In particular, these parameters determine whether engineered NP are stabilized, homo- or heteroagglomerate with other particulates, sediment, or remain dispersed.

For the present study, we chose Ag NP and CeO_2_ NP as model particles based on usage, the availability of data, and the difference in NP properties. Ag based NP are used in the highest number of NP containing consumer products [16] due to their bactericidal properties [17], which have been attributed to both the NP as well as to Ag^+^ released in aqueous media [18]. Ag^+^ release from Ag NP depends on NP surface modification and the dispersant [19], [20]. We selected carbonate stabilized Ag NP which have been characterized regarding stability in artificial and natural stream water as well as toxicity to the model green alga *Chlamydomonas reinhardtii* [10], [21]. As the NP were stabilized with carbonate we were able to use the carbonate-bicarbonate buffer to adjust the pH during our experiments, which is the dominant buffer system in streams [22]. CeO_2_ based NP have been studied due to Ce^III^-Ce^IV^ redox-cycling in NP surface atoms, which is associated with the capacity to store and release oxygen [23]. Thus CeO_2_ NP are used for example for the oxidative destruction of organic compounds in waste water [24] and have been suggested for biomedical applications involving redox processes [25], [26]. Recently, CeO_2_ NP have successfully been used to enhance the effect of chemotherapeutics on cancer cells and at the same time protect intact cells *in vitro* [27].

Any material reaching periphyton by settling from the overlaying water will first encounter the extracellular matrix secreted by periphytic organisms. It is made up of hydrated extracellular polymeric substances (EPS) consisting mostly of species-specific polysaccharides and proteins [28], [29], [30], that mediate adhesion of periphytic organisms to abiotic surfaces and other organisms. Up to 90% of the organic matter associated with biofilms can be EPS [31]. EPS of algal and bacterial monoculture biofilms have been shown in some cases to stabilize Ag NP [32], as well as under other conditions to induce Ag NP agglomeration [33]. EPS have been suggested to trap Ag NP outside bacterial cells possibly lowering their toxicity [33]. Thus, to understand the fate and effects of NP in periphyton, it is essential to study their interaction with the periphytic EPS.

The aim of this study was to investigate the influence of periphytic EPS on physicochemical properties of selected NP over time depending on exposure conditions relevant to the photoheterotrophic community. We exposed CeO_2_ and Ag NP to chemically characterized EPS extracted from five independent periphyton communities to account for inter-community variability of EPS. We assessed changes in NP physicochemical characteristics depending on NP material, EPS extract, exposure time and conditions (light, pH).

## Materials and Methods

### Chemicals

If not stated below, chemicals were purchased from Sigma-Aldrich Switzerland.

### Nanoparticles

Carbonate-coated Ag NP with a primary particle size of 25 nm (Table S1) have been described previously [10], [21] and were provided by NanoSys GmbH (Wolfhalden, Switzerland) as an aqueous suspension with a nominal concentration of 1 g/L (9.27 mM) Ag. The original suspension was kept in the dark. Unmodified CeO_2_ NP with a nominal primary particle size of 25 nm were provided by Nanograde AG (Staefa, Switzerland) (Table S1).

### Periphyton colonization

Periphyton was colonized on glass microscope slides (76×26 mm, Thermo Scientific) which were soaked in 0.03 M HNO_3_ and washed with nanopure water (18.1 MΩ•cm, Milli-Q) before use and were then placed horizontally in Plexiglas channels (86×10.4×10 cm, polymethyl methacrylate) in a flow-through system fed by water from river Chriesbach (on campus, Dübendorf, Switzerland) [34], [35]. An overview of Chriesbach water chemistry is provided in Table S2. A sediment trap (0.51×0.7×2.6 m, residence time ∼20 min) was used to remove part of the dispersed particles. The flow rate in the channels was maintained at ∼1 cm/s corresponding to a volume flow of 3 L/min. Typical flow rates in the benthic zone of Chriesbach are 1–4 cm/s. Illumination was provided in 12∶12 h light/dark cycles by BioSun fluorescent tubes with a radiation similar to the sunlight spectrum (Radium Lampenwerk GmbH, Germany, ML-T8 36W/965/G13B; photon flux during light periods: 100 µE/m^2^s). Temperature and photosynthetically active radiation (PAR) in the channels were monitored by a HOBO Pendant Temperature/Light Data Loggers (UA-002-64). Oxygen saturation was monitored with a Presens Microx TX3 system and an NTH-PSt1-L2.5-TS-NS40/0.8-YOP-EXT1 oxygen microsensor.

### Extraction and characterization of extracellular polymeric substances (EPS) from periphyton

Periphyton was colonized five times independently for three weeks each time in 2011 and 2012. EPS were extracted from periphyton grown on 64 glass slides (Table 1). EPS extracts were analyzed for cell lysis, organic carbon and organic nitrogen size distribution, and protein, calcium, magnesium, and chloride content.

**Table 1:** Properties of EPS extracts 1–5 as determined by weighing of dry mass, protein quantification and LC-OCD-OND.

	ExtractionDate	Dry mass[g/slide]	DOC/drymass [mg/g]	cDOC[% DOC]	Protein/drymass [mg/mg]	DOC/N(biopolymers)	DOC/protein	N/protein	Biopolymers[% cDOC]	HS building blocks[% cDOC]	Low M_r_ acids[% cDOC]	Neutrals,amphiphilics[% cDOC]
1	05.09.2011	0.06	0.86	94.6	0.07	10.5	14.5	1.4	14.2	15.3	10.3	54.8
2	04.10.2011	0.04	0.71	89.9	0.07	3.9	14.0	3.6	8.8	20.0	6.2	54.9
3	24.10.2011	0.02	1.75	100.3	0.011	5.9	9.3	1.6	15.4	25.3	10.9	48.7
4	11.04.2012	0.03	1.11	89.6	0.20	1.2	5.1	4.3	4.5	21.4	3.2	60.5
5	02.05.2012	0.03	1.14	93.1	0.08	3.3	13.1	4.0	10.3	24.3	13.6	44.9

DOC: dissolved organic carbon, cDOC: chromatographable DOC, N: nitrogen, HS: humic substances, M_r_: molecular weight.

The extraction procedure was adapted from Stewart et al [30]. After 21 days of colonization, the biofilms were scraped off the glass slides with a clean glass slide into 1 mL/slide NaHCO_3_ (2 mM, pH 7.6) in a 100 mL glass beaker. The buffer contained 1 µg/mL protease inhibitors (1∶1:1 Aprotinin (A2132.0010), Leupeptin (L9783), and Pepstatin A (A2205.0010); AppliChem AG). The biomass was resuspended by gentle pipetting and sonication in a water bath (45 kHz 60 W, VWR Ultrasonic Cleaner) for 30 s. Fine sediment and larger biomass was allowed to settle for ∼1 min, the supernatant was removed and centrifuged at 1,880·g for 10 min. Biomass was resuspended a second time in 1 mL/slide fresh solution and treated as described above. The resulting biomass pellets were frozen at −80°C and subsequently lyophilized to determine dry weight. All supernatants were sequentially filtered (1 µm glass fiber [VWR], 0.45 µm polypropylene [PALL], and 0.22 µm PES [Millipore] filters). Filters were washed with nanopure water (18.1 MΩ•cm, Milli-Q) prior to use. The procedure resulted in a final volume of about 130 mL per extract. EPS extracts were stored in glass bottles at 4°C (0.02% (w/v) NaN_3_). All glassware used for EPS extraction was muffled before use (450°C, 4 h). All extraction steps were performed on ice, the water bath for ultrasound treatment was at room temperature.

To verify that no measurable cell lysis occurred during the extraction procedure, glucose-6-phosphate dehydrogenase (G6P-DH) activity was measured as described previously [30], [36]. G6P-DH is an intracellular enzyme that would be released upon cell lysis. At each step of EPS extraction, 20 µL samples were transferred to a 96-well plate in triplicate (Greiner bio-one, black, transparent flat bottom, non-binding, 655906). 180 µL reaction mixture (50 mM Tris Base, 0.15 mM NADP (Merck 481972), 10 mM MgCl_2_, and 3 mM α-D-glucose-6-phosphate (Merck 346764); pH 8) were added and light absorption of NADPH being formed was measured at 340 nm at 30°C for 30 min. G6P-DH (*Leuconostoc mesenteroides*, Merck, 346774-500U) calibration curves were generated in both water and in the EPS extract to verify that the EPS did not interfere with the detection of G6P-DH activity. G6P-DH activity was below the detection limit in all EPS extracts (limit of detection: 0.00125 U/mL). Lysed periphyton samples served as positive controls as described previously [30].

Organic carbon and nitrogen size distribution was measured by size-exclusion chromatography – organic carbon detection – organic nitrogen detection (LC-OCD-OND) as described previously [30]. Samples were diluted 1∶50 with nanopure water (18.1 MΩ•cm, Milli-Q) right before they were measured. A size exclusion column (250×20 mm, Toyopearl TSK HW-50S) was used to separate EPS compounds. To quantify the carbon background of the extraction protocol, an aliquot of extraction buffer was treated the same way as periphyton suspensions and then assessed by LC-OCD-OND. The mobile phase was phosphate buffer (24 mM, pH 6.6) and the acidification solution was phosphoric acid (60 mM, pH 1.2). The detection limit was 10 µg/L for both OC and ON. Retention times of 35–70 min correspond to 70–0.5 kDa model proteins and 28–0.1 kDa model polysaccharides [30]. The software FIFFIKUS was used to quantify total organic carbon (TOC), dissolved (DOC), and chromatographable DOC compounds (cDOC). The chromatograms obtained from LC-OCD-OND are integrated to determine the amount of biopolymers (high M_r_ polysaccharides and proteins), humic substances (HS), building blocks of HS, low M_r_ acids, and amphiphilic/neutral compounds (alcohols, aldehydes, amino acids, and ketones). As an example of the reproducibility of the EPS extraction procedure Figure S4 shows chromatograms of two EPS extracts obtained independently by two individuals on the same day from two different channels after three weeks of colonization.

Total protein in EPS extracts was measured by the Bradford assay [37] using Bradford reagent (Bio-Rad Protein Assay Kit I). Calibration curves were produced with bovine serum albumin (BSA) diluted in equal amounts of EPS extracts to account for any interference of the EPS with protein detection.

Calcium, magnesium, and chloride content of EPS extracts was quantified by ion chromatography (Metrohm 761 Compact IC; with chemical suppression for Cl^−^ detection). Detection limits were: 5 mg/L Ca^2+^, 2.5 mg/L Mg^2+^, and 0.5 mg/L Cl^−^.

### Exposure of CeO_2_ and Ag NP and AgNO_3_ to EPS

CeO_2_ NP, Ag NP, and AgNO_3_ were dispersed in 20 mL 2 mM NaHCO_3_ at three different pH values (pH 6, 7.6 or 8.6) with or without EPS (10 mg/L DOC) in light (100 µE/m^2^s, BioSun fluorescent tubes, see above) or dark resulting in 12 exposure conditions. Samples were prepared in triplicates (n = 3) and were placed in a climate room at 20°C and stirred at 400 rpm (vessels: SNAP-CAP, Huber Switzerland, 8.9227.08; magnetic stir bars, with PTFE coating, 6×20 mm, Huber Switzerland, 13.1120.06). pH was adjusted with HNO_3_ and NaOH at the beginning of the experiments and was constant during exposure. Dispersions contained 0.02% (w/v) NaN_3_. Final nominal NP concentrations were 5 and 0.5 mg/L for both types of NP and 7.87 and 0.78 mg/L AgNO_3_ (4.6×10^−5^ and 4.6×10^−6^ mol L^−1^). Actual concentrations were determined by inductively coupled plasma mass spectrometry (ICP-MS) and used to calculate total and dissolved Ce and Ag during the exposure (see Characterization of NP dispersions below). CeO_2_ dispersions were generated from a stock dispersion (50 mg/L). The stock dispersion was made directly before use by adding 20 mL 2 mM NaHCO_3_, pH 7.6 to 1 mg CeO_2_ and stirring for 1 h at daylight conditions. Ag NP were freshly diluted to final concentrations from the original dispersion while stirring. AgNO_3_ was freshly diluted from a 0.3 g/L stock solution which was kept in the dark.

Experiments were conducted with five different EPS extracts (see Table 1). EPS extracts were used on the day after the extraction. All glassware used was muffled before use (450°C, 4 h). O_2_ concentrations in the samples were measured as described above. Average O_2_ saturation did not significantly change over time or with sample properties (light/dark, pH, EPS) and were 71.6±1.5% (∼6.18 mg/L; Ag NP dispersions), 69.4±3.5% (∼5.99 mg/L; AgNO_3_ suspensions), and 71.1±1.7% (∼6.14 mg/L; CeO_2_ dispersions).

### Characterization of nanoparticle dispersions

NP hydrodynamic diameter was determined by dynamic light scattering (DLS) and nanoparticle tracking analysis (NTA). NP zetapotential was derived from electrophoretic mobility (EPM) measurements. Light absorption of Ag containing dispersions was measured by UV-VIS spectroscopy. Selected samples were analyzed by transmission electron microscopy/energy dispersive X-ray analysis (TEM/EDX) and X-ray absorption spectroscopy (XAS) to determine size, shape, elemental composition and Ag speciation.

The concentration of dissolved Ag and Ce in samples containing 5 mg/L Ag or Ce was determined by ICP-MS.

Samples for NP characterization were taken at 3 h, 24 h, 48 h, 168 h, and 336 h (DLS, NTA, UV-VIS; see below) or 5 min ( = “0 h”), 168 h and 336 h (ICP-MS, see below).

Table S4 provides an overview of the sampling scheme.

### Dynamic light scattering (DLS) and electrophoretic mobility (EPM)

The hydrodynamic diameter of the NP was derived from DLS measurements and the zetapotential from the EPM, both measured on a Zetasizer Nano ZS (nano ZS, Malvern Instruments). For comparison with data from the literature, the Smoluchowski model was used to calculate the zetapotential from EPM measurements. To compare results within this study we use EPM due to the difference in systems being compared (with and without EPS). Three measurements were taken per sample and the autocorrelation function was analyzed using the cumulant analysis algorithm resulting in a mean size (z-average) and a standard deviation (polydispersity index, PDI). Data sets with a PDI above 0.5 were not taken into account (for settings see supplementary information). DLS measurements performed on aqueous suspension containing EPS did not indicate any particles. Representative count rates for Ag NP, CeO_2_ NP and NP formed in AgNO_3_ are provided in Tables S6–S8.

### Nanoparticle tracking analysis (NTA)

NTA (NanoSight LM10 equipped with a LM14 temperature controller, NanoSight Ltd.) was used to determine a number based particle size distribution. Each sample was directly measured three times for 60 s without any further processing (no dilution). All NTA videos were analyzed with the same settings in batch processing mode (for settings see supplementary information). Analyses that resulted in less than 200 tracked particles were not used. Settings were adapted to not detect any EPS particles. Videos were analyzed using the NanoSight NTA 2.2 and 2.3 Analytical Software (NanoSight Ltd.).

### UV-VIS absorption

UV−VIS light absorption (190–900 nm) of dispersions and AgNO_3_ was recorded with a UVIKON 930 spectrophotometer (Kontron Instruments). EPS and NaHCO_3_ were measured as controls. Ag NP show size- and surface-specific surface Plasmon resonance (SPR) which results in a specific light absorption spectrum [38].

### Transmission electron microscopy (TEM)

Selected samples were analyzed by TEM in combination with an energy dispersive x-ray (EDX) analysis system to determine the size and the elemental composition of the particles. For that purpose, a drop of suspension was drawn through a lacy carbon-coated TEM grid (holey Carbon Copper grids, mesh size 200, Plano GmbH, Wetzlar, Germany) with a paper tissue. Samples were analyzed using a TEM (Tecnai F30 STEM, FEI) equipped with an EDX system (EDAX, New Jersey, USA).

### X-ray absorption spectroscopy (XAS)

XAS analyses at the Ag K-edge were performed on the Dutch Belgian Beamline (Dubble, BM01B) at the European Synchrotron Radiation Facility (ESRF, Grenoble, France). Frozen samples were measured with a He-Cryostat adjusted to 80 K. Reference samples were prepared as pellets (Ag_2_S (Alfa Aesar; 011416)), or as suspensions (Ag NP). XAS data extraction and processing was performed using Athena [39] following standard procedures as described previously [40].

### Sample preparation for ICP-MS

NP and AgNO_3_ dispersions were centrifuged in 2 mL reaction tubes to separate particles (>3.5 nm) and dissolved ions (25°C and 20,800·g for 3 h (Ag, ρ_Ag_ 10.5 g/cm^3^) or 4.6 h (CeO_2_; ρ_CeO2_ 7.65 g/cm^3^). Ultrafiltration was not applicable in this study as we observed varying degrees of sorption of Ag to ultrafiltration membranes (Amicon Ultra-4 Centrifugal Filter Unit with Ultracel-3 membrane, Millipore) depending on pH and presence or absence of EPS. The supernatants (0.6 mL) were transferred to new reaction tubes, acidified (0.65% HNO_3_), and stored at 4°C. Non-centrifuged samples for the determination of total NP and AgNO_3_ concentration were stored in the same manner. All samples were treated by microwave digestion prior to ICP-MS analysis. 0.5 mL of each sample were digested with 4 mL of 65% HNO_3_ and 0.5 mL of 30% H_2_O_2_ in a microwave digestion unit (MLS ultraClave 4; 10 min at 180°C/100 bar, 14 min at 210°C/100 bar) and diluted 1∶100 with nanopure water. (18.1 MΩ•cm, Milli-Q). One sample per run contained only HNO_3_, H_2_O_2_, and EPS diluted in NaHCO_3_ to determine the background concentration of Ce and Ag.

### Inductively coupled plasma mass spectrometry (ICP-MS)

The total and dissolved Ag and Ce concentrations in NP and AgNO_3_ dispersions were measured by ICP-MS (Element 2 High Resolution Sector Field ICP-MS; Thermo Finnigan). The instrument was calibrated with a multi-element mass standard (Merck, 1113550100). The calibration curve for data analysis was made with the calibration standard SCP-33-MS (140-130-321, PlasmaCAL) in the concentration range 0–20 µg/L. A reference with a concentration within the calibration range was measured every 10 samples, the calibration samples were measured every 40 samples. The detection limit of Ce and Ag was 0.01 µg/L. Mean recovery was 88.4±4.4% for total Ce, 96.9±3.7% for total Ag from Ag NP, and 96.5±3.4% for AgNO_3_.

### Data analysis

Calculation of averages, standard deviations, percentages and oneway ANOVA and Tukey posthoc test or unpaired t test (both allowing a significance level α = 0.05) were performed with Microsoft Excel 2010 and GraphPad Prism 4 for Windows. Graphs were generated with the same software programs.

## Results

### Characterization of EPS extracts

EPS were extracted from five different 3-week-old communities between 09/2011 and 05/2012 (Table 1). The average water temperature of the corresponding growth periods ranged from 11.9°C to 18.3°C and is provided in the supplement (Figure S1).

Dry mass ranged from 0.02–0.06 g/slide with the highest mass measured in extract 1 at the beginning of September 2011. DOC concentration in EPS extracts ranged from 0.71–1.75 mg/g dry mass and protein concentrations ranged from 0.007–0.025 mg/mL. The ratios of DOC to organic nitrogen compounds (DOC/N) and to the amount of proteins (DOC/protein) were in the range of 1.2–10.5 and 5.1–14.5, respectively. Extract 4 of April 2012 had the lowest and extract 1 of September 2011 had the highest ratios of DOC to N or protein.

Chromatograms of all EPS extracts obtained by LC-OCD-OND showed four distinct features (Figure S2) which have been reported to correspond to high M_r_ biopolymers, building blocks of humic substances (HS), low M_r_ acids, and small amphiphilic/neutral compounds [30], [41]. The retention time and shape of the four features varied between the five extracts. Low M_r_ acids and amphiphilics/neutrals showed less variability in peak retention time and shape than biopolymers and HS building blocks. Extract 4 from April 2012 showed longer retention time in all four groups of components than extracts 1–3 and 5. In particular, the biopolymer signal appeared after 30 min retention time instead of after 25 min as observed in extracts 1–3. Extract 5 also lacked a biopolymer peak between 25–30 min retention time but had a biopolymer concentration in the same range as extracts 1–3. The UV-signal of extract 4 was lower than in the other samples and the peaks were less pronounced as well as shifted to longer retention times (Figure S3).

The percentages of the four groups of EPS components in each extract are listed in Table 1. The concentration of amphiphilic and neutral compounds was typically the highest followed by HS-building blocks, biopolymers, and low M_r_ acids. Extract 5 was an exception in having a higher fraction of low M_r_ acids than biopolymers. Extract 4 had the highest amount of neutral/amphiphilic compounds and the lowest percentage of all other components. cDOC/N ratios in the biopolymer fraction ranged from 1.2–10.5 with the lowest ratio observed in extract 4.

### Influence of EPS on the stability of CeO_2_ NP dispersions

**Figure 1:**
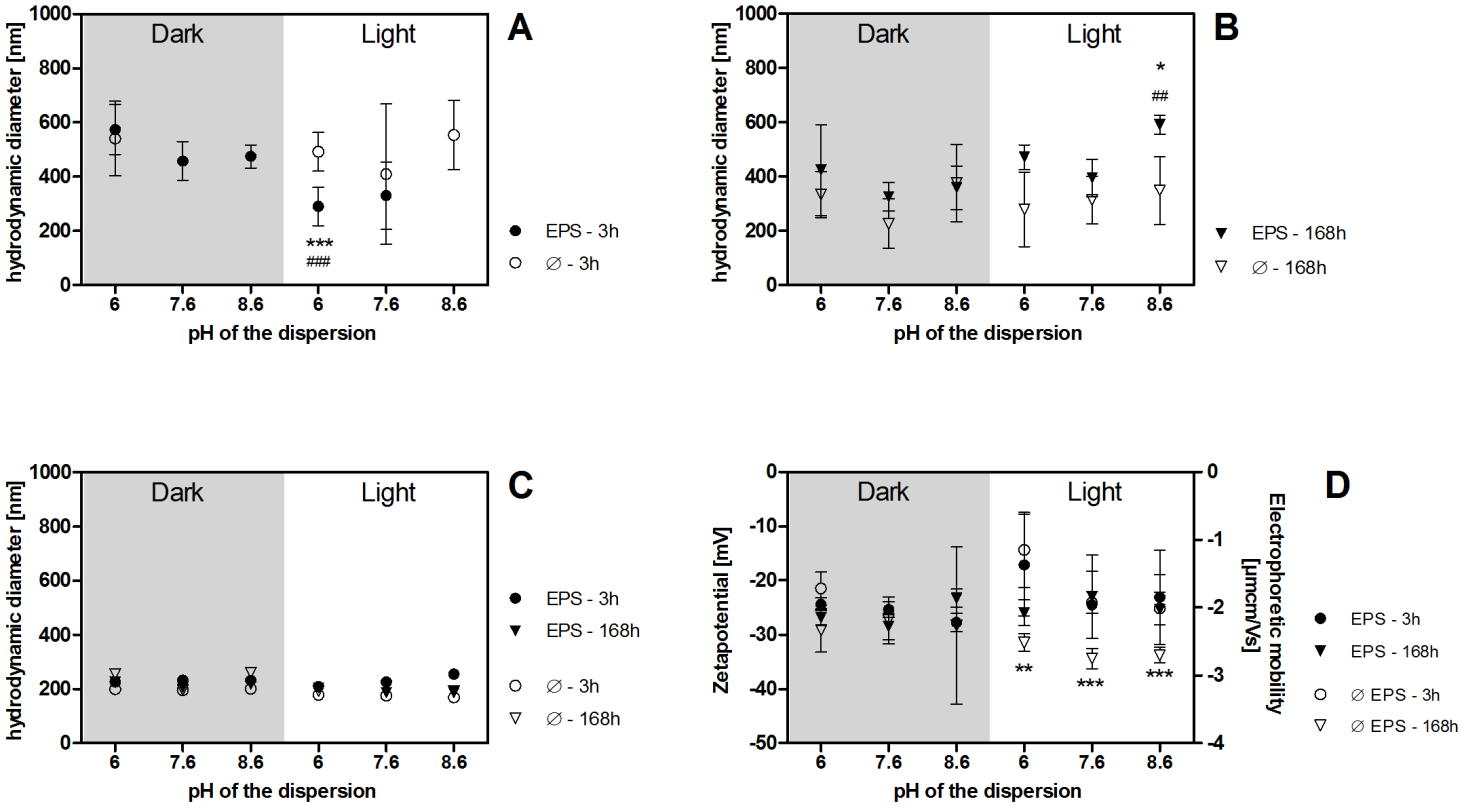
A–D: Mean hydrodynamic diameter of CeO_2_ NP with standard deviations in 5 mg CeO_2_/L dispersions as determined by DLS (z-average/intensity distribution; **A, B**) and NTA (mean size, **C**) and mean EPM and zetapotential (**D**) with standard deviations determined by electrophoresis/DLS. Samples were incubated in the dark or in light (100 µE/m^2^s). n = 15 from 5 independent experiments with 3 replicates each, each experiment was done with one EPS extract (10 mg/L). 3 h DLS results in panel **A** for CeO_2_ NP without EPS at pH 7.6 and 8.6 in the dark and for CeO_2_ NP with EPS at pH 8.6 in the light were discarded due to high polydispersity. **A** ** significantly different from light-treated samples without EPS and ### significantly different from dark-treated sample with and without EPS, p<0.0001, df = 11 (between groups), F = 15.79. **B** * significantly different from light-treated samples without EPS, ^##^ significantly different from dark-treated samples with and without EPS, p = 0.001, dg = 11 (between groups), F = 6.52. **D** 168 h: ** (pH 6) and *** (pH 7.6 and 8.6) significantly different from light-treated samples without EPS, p<0.0001, df = 11 (between groups), F = 11.62.

The mean hydrodynamic diameter of CeO_2_ NP dispersed in 2 mM NaHCO_3_ with and without EPS as determined by DLS ranged from ∼200 nm to ∼600 nm (Figure 1 A, B, Tables S9, S10). In EPS-free samples, CeO_2_ NP mean hydrodynamic diameter decreased over time independent of light and pH conditions (from 540 nm [3 h] to 226–376 nm [168 h] in the dark and from 409–553 nm [3 h] to 278–349 nm [168 h] in the light). With all EPS extracts, mean particle sizes were higher in the dark than in the light after 3 h (457–574 nm and 290–330 nm, respectively). Over time, mean sizes decreased slightly in the dark but increased in the light (325–423 nm and 395–591 nm, respectively).

The mean hydrodynamic diameter as determined by NTA was ∼200 nm, did not depend on pH or light/dark, or the presence of EPS and did not change over time (Figure 1 C, Tables S9, S10).

The mean EPM of CeO_2_ NP incubated in the dark without EPS ranged from −1.68±0.49 µmcm/Vs (pH 6) at 3 h to −2.28±0.24 µmcm/Vs (pH 6) at 168 h (Figure 1 D, Tables S9, S10). The differences between pH 6, 7.6, and 8.6 were not statistically significant. In the presence of EPS in the dark, EPM ranged from −1.91±0.09 µmcm/Vs (pH 6) at 3 h to −2.11±0.11 µmcm/Vs (pH 6) at 168 h. The differences between pH 6, 7.6, and 8.6 were not statistically significant.

In light-treated samples without EPS, EPM ranged from −1.12±0.45 µmcm/Vs (pH 6) at 3 h to −2.46±0.13 µmcm/Vs (pH 6) at 168 h and from −2.0±0.73 µmcm/Vs (pH 6) at 3 h to −1.87±0.18 µmcm/Vs (pH 6) at 168 h in the presence of EPS. After 168 h, EPM were statistically significantly lower without EPS than with EPS. The only statistically significant difference between different pH conditions was between light-exposed samples at pH 6 and pH 7.6/8.6 both in the presence and absence of EPS.

**Figure 2:**
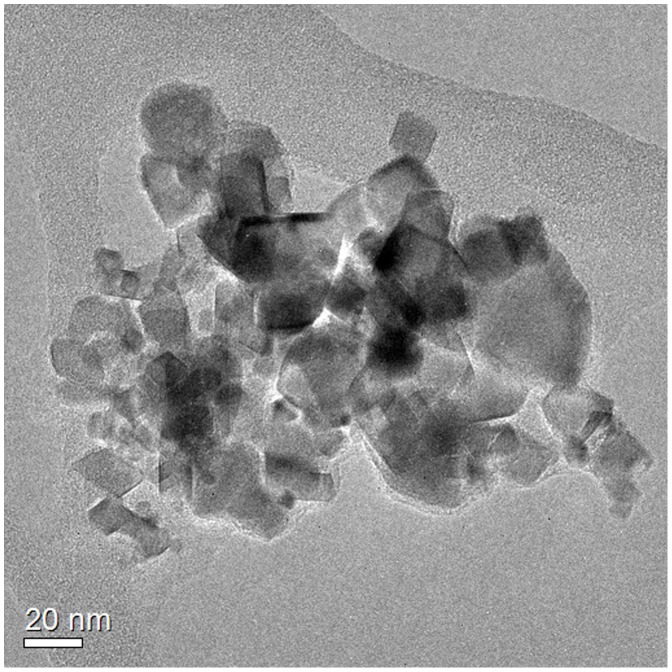
TEM bright field image of CeO_2_ NP after 24 h of stirring (5 mg/L, pH 7.6, 2 mM NaHCO_3_).

TEM analysis of the CeO_2_ suspensions revealed that the NP consisted of 10 −50 nm spherical, cuboidal, and octahedral primary particles that were fused together (Figure 2).

Results for 0.5 mg/L CeO_2_ NP dispersions showed the same trends regarding mean hydrodynamic diameter and EPM as dispersions containing 5 mg/L CeO_2_ NP (Figure S8, Tables S9, S10).

### Release of Ce^III^ from CeO_2_ NP in the presence of EPS

**Figure 3:**
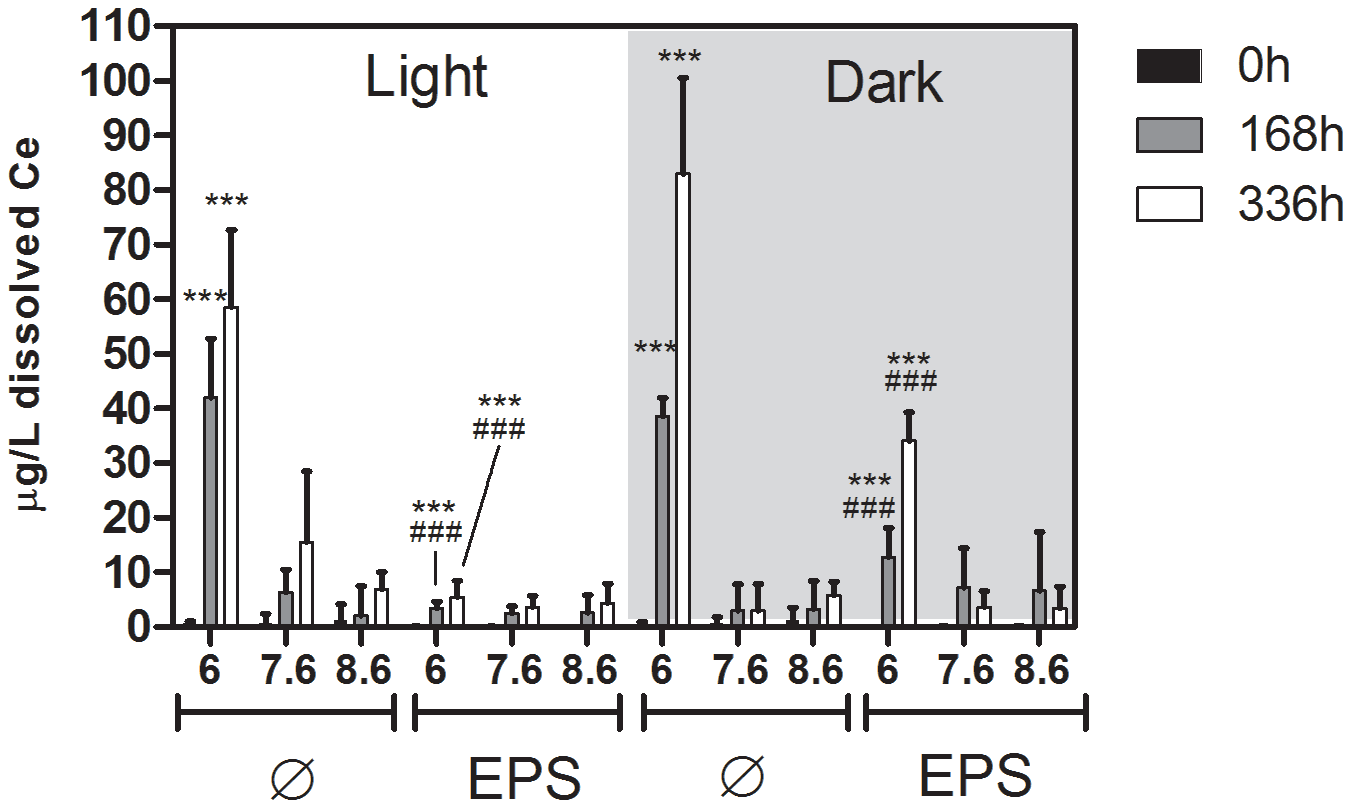
Mean mass concentrations with standard deviations of dissolved Ce in 5 mg/L CeO_2_ NP dispersions directly after addition of NP (,,0 h”) and after 168 h and 336 h of incubation as determined by ICP-MS of acid-digested samples. Samples were incubated in the dark or in light (100 µE/m^2^s). n = 9 in 3 independent experiments, each experiment was done with one EPS extract. *** Significant difference between EPS-free and EPS containing samples; ### significant difference between light and dark treated samples, 168 h: p<0.0001, df = 11 (between groups), F = 52.55, 336 h: p<0.0001, df = 11 (between groups), F = 25.65.

At 0 h, dissolved Ce (<0.01 −1 µg Ce/L, or <0.01 −0.03% of total Ce; Figure 3) was close to or below the detection limit. After 168 h and 336 h, all samples contained at least 3 µg/L dissolved Ce (∼0.1% of total Ce). At pH 6, the average concentration of dissolved Ce increased to 58.6±14.1 µg/L (∼1.6%) in the light and 82.9±17.6 µg/L (∼2.4%) in the dark. In EPS containing samples, the concentration of dissolved Ce did not increase significantly at pH 6 as compared to pH 7.6 and 8.6 in the light [5.5±2.9 µg/L (∼0.2%)] but increased to 34.0±5.2 µg/L (∼1%) in the dark. Analyses after 336 h showed a further increase in dissolved Ce at pH 6 in EPS-free samples in the light and the dark as well as in EPS containing samples in the dark. Overall, the concentration of dissolved Ce at pH 6 was significantly different between light and dark treated samples with EPS but not between EPS-free samples incubated in light or dark.

### Influence of EPS on the stability of Ag NP dispersions

**Figure 4:**
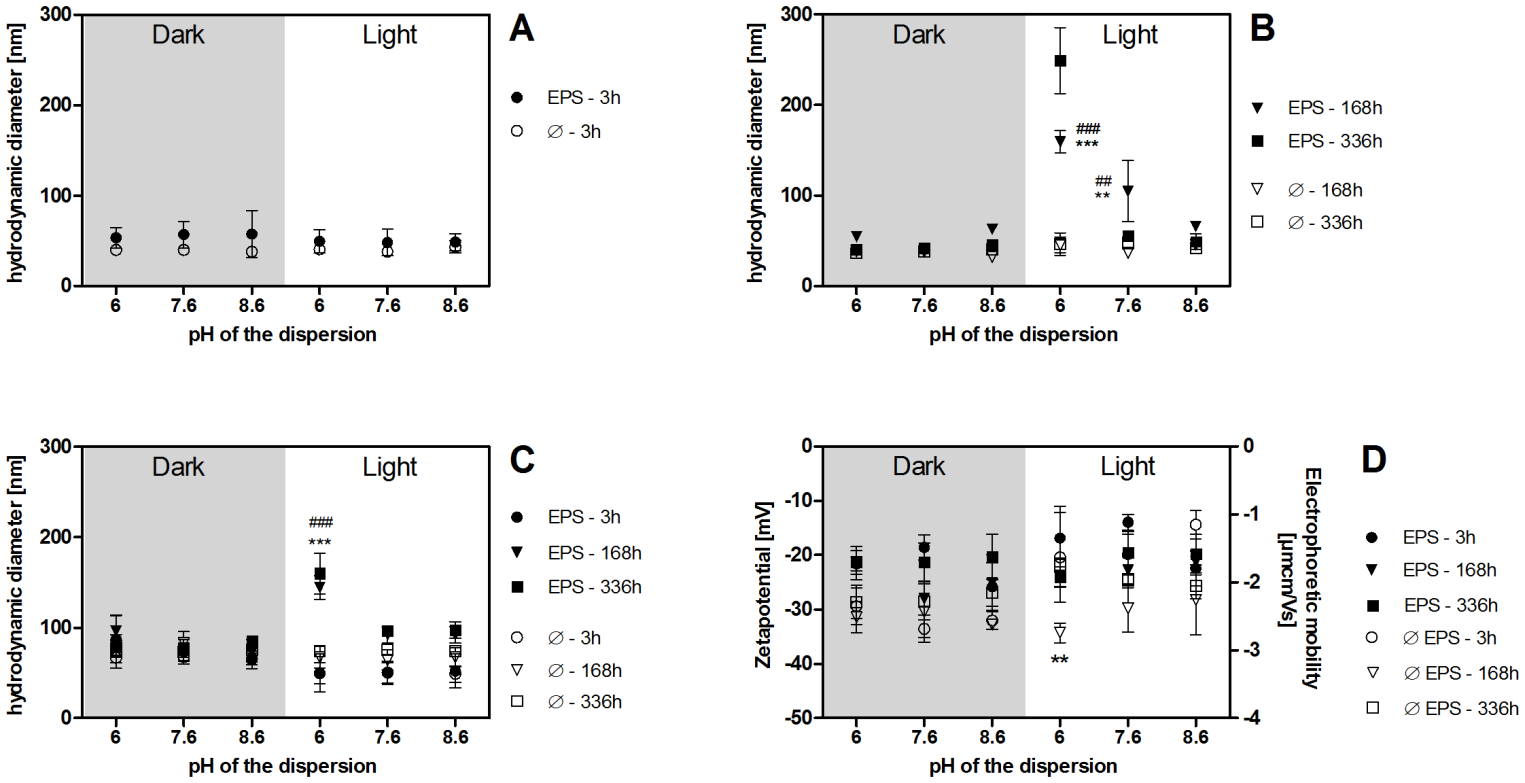
A–D: Mean hydrodynamic diameter of Ag NP with standard deviations in 5 mg Ag NP/L dispersions with and without 10 mg DOC/L EPS as determined by DLS (z-average/intensity distribution; **A, B**) and NTA (mean size, **C**) and mean EPM and zetapotential (**D**) with standard deviations determined by electrophoresis/DLS. 3 h: n = 15, 5 independent experiments (E1–5). 168 h: n = 12, 4 independent experiments (E1–3, 5). 336 h: n = 3, 1 experiment (E4). Each experiment was done with one EPS extract. Samples were incubated in the dark or in light (100 µE/m^2^s). **B** 168 h: *** and ** significantly different from light-treated samples without EPS, ^###^ and ^##^ significantly different from dark-treated samples with and without EPS, p<0.0001, df = 11 (between groups), F = 49.51. **C** 168 h: *** significantly different from light-treated samples without EPS and ^###^ significantly different from dark-treated samples with and without EPS, p<0.001, df = 11 (between groups), F = 77.83. **D** 168 h: * significantly different from light-treated samples with EPS, p<0.001, df = 11 (between groups), F = 4.241.

According to DLS measurements, the mean hydrodynamic diameter of Ag NP neither changed in EPS-free dispersions over time nor depending on light or pH conditions (Figure 4 A–B; mean diameter: 40±2 nm after 3 h, 40±5 nm after 168 h; Tables S11, S12). The mean hydrodynamic diameter was slightly higher with EPS at 3 h (53±4 nm) but remained unchanged over time in dark treated samples. Incubation in light led to an increase in mean hydrodynamic diameter with decreasing pH within 24 h in the presence of EPS extracts 1–3 and 5. Mean hydrodynamic diameters remained stable until the end of the test period (168 h: pH 6∶159±13 nm; pH 7.6∶105±34 nm; pH 8.6∶66±4 nm). Changes in the mean hydrodynamic diameter occurred later with EPS extract 4 which had the lowest concentration of biopolymers and lowest C/N ratio in the biopolymer fraction. Here, a pH- and light-dependent increase was detectable only between 168 h and 336 h of incubation (Figure 4 B, 336 h values; Tables S11, S12).

NTA data support these observations but showed smaller differences in mean hydrodynamic diameter. The mean hydrodynamic diameter of Ag NP hardly changed in EPS-free dispersions over time (Figure 4 C; 63±15 nm after 3 h, 58±9 nm after 168 h; Tables S11, S12) and slightly decreased in EPS containing dispersions in the dark (81±13 nm after 3 h, 72±8 nm after 168 h). As already described based on DLS data, incubation in light led to an increase in mean size with decreasing pH, however, pH 7.6 and pH 8.6 dispersions were not significantly different (mean sizes at 168 h: pH6∶144±13 nm; pH 7.6∶92±9 nm; pH 8.6∶92±9 nm). Again, EPS extract 4 (lowest biopolymer concentration, lowest C/N ratio in the biopolymer fraction) led to a slower change in hydrodynamic diameter with a pH- and light-dependent increase being detectable only between 168 h and 336 h of incubation (Figure 4 C, 336 h values; pH 6∶160±22 nm; pH 7.6∶96±2 nm; pH 8.6∶97±9 nm).

Results for 0.5 mg/L Ag NP dispersions are not displayed as they showed the same trends regarding mean hydrodynamic diameters and EPM as dispersions containing 5 mg/L Ag NP.

**Figure 5:**
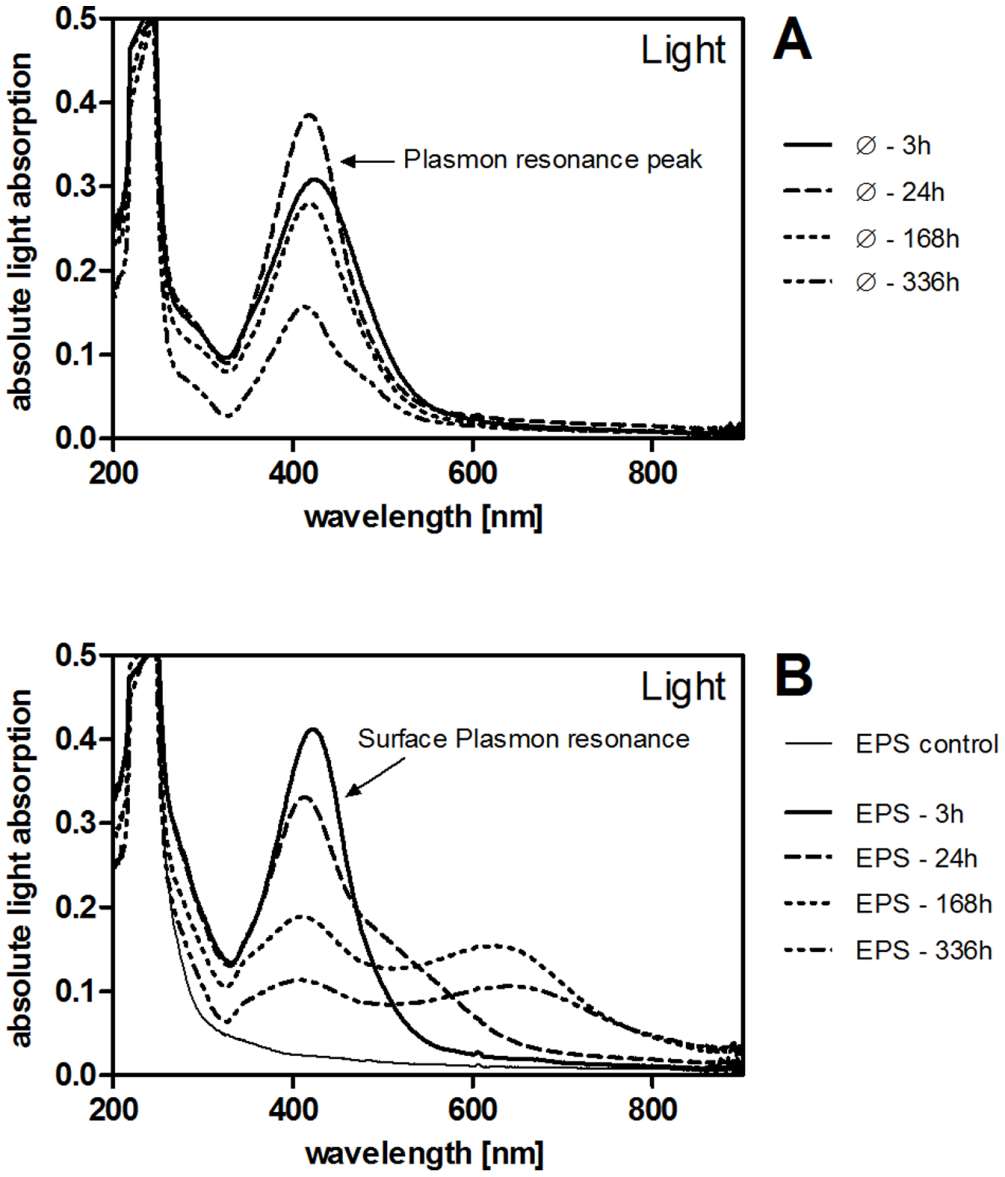
A–B: Spectra of absolute light absorption of 5 mg/L Ag NP as determined by UV-VIS spectroscopy. Samples were incubated in light (100 µE/m^2^s) at pH 6 without (**A**) or with EPS (**B**, 10 mg DOC/L; 3–168 h spectra obtained with EPS extract 1; 336 h spectrum obtained with EPS extract 4). EPS control (**B**) contained 10 mg DOC/L EPS in 2 mM NaHCO_3_. Different EPS extracts did not differ in absorption of light >300 nm.

The UV-VIS light absorption of Ag NP is indicative of changes in their size and/or surface due to SPR of surface electrons interacting with photons of defined wavelengths. Figure 5 shows light absorption spectra of Ag NP in EPS-free dispersions (A) and EPS-containing dispersions (B). All samples showed strong light absorption around 250 nm. Ag NP containing samples absorbed light around 410–420 nm corresponding to the SPR peak of the primary Ag particles. EPS alone also showed strong light absorption around 250 nm but did not have a second light absorption peak around the SPR signal of Ag NP (Figure 5 B). In the presence of EPS, a second absorption maximum appeared around 650 nm in Ag NP dispersions after 24 h. The change in the absorption spectrum occurred later in the presence of the EPS extract with the lowest biopolymer concentration and lowest C/N ratio in the biopolymer fraction (extract 4; Figure 5 B, 336 h spectrum).

TEM imaging of selected Ag NP samples treated with EPS in the light showed both aggregated NP. In addition, also the size of the primary particles seemed to have increased. However, due to the limited amount of TEM data, this increase remains on a qualitative level. No change of the original spherical shape of the NP was observed (Figure S5A, S5B).

XAS and EDX analyses of selected samples showed that Ag NP treated with EPS were dominantly elemental Ag (Figure S5C, S5D, and S6, Table S5). The LCF fits of the XANES and EXAFS spectra always returned minor fractions (typically 5–10%) of other Ag phases (Ag_2_S and Ag_2_O). However, the uncertainty of the LCF analysis is in the same range and thus, whether additional Ag phases are present cannot be assessed based on the available dataset.

### Dissolved Ag^+^ in Ag NP dispersions

**Figure 6:**
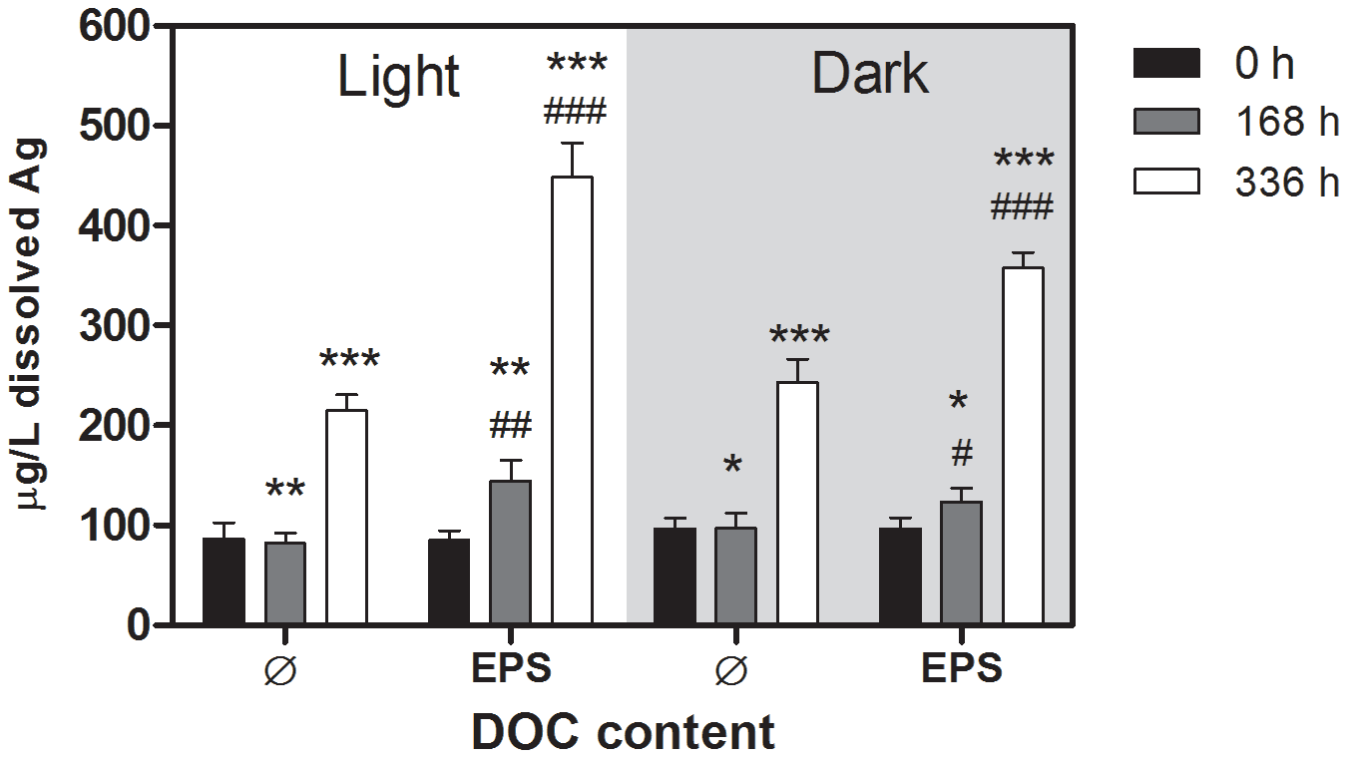
Mean mass concentrations with standard deviations of dissolved Ag in 5 mg/L Ag NP dispersions directly after addition of NP (,,0 h”) and after 168 h and 336 h of incubation as determined by ICP-MS of acid-digested samples. Samples were incubated in the dark or in light (100 µE/m^2^s). n = 9 from 3 independent experiments, each experiment was done with one EPS extract (10 mg DOC/L). * Significant difference between EPS-free and EPS containing samples; # significant difference between light and dark treated samples, 168 h: p = 0.0012, df = 3 (between groups), F = 5.774, 336 h: p<0.0001, df = 3 (between groups), F = 66.56.

The average concentration of dissolved Ag^+^ in Ag NP dispersions was ∼90 µg/L (∼2% of total Ag measured) at the start of the experiments and did not depend on the pH of the dispersion (pH 6, 7.6, or 8.6) or the presence of EPS (Figure 6). After a week of incubation, the concentration of dissolved Ag remained unchanged in EPS-free samples but had increased to 144±21 µg/L (∼3.2% of total Ag) in the light and to 123±14 µg/L (∼2.7% of total Ag) in the dark. Within the second week of incubation, the concentration of dissolved Ag also increased in EPS-free samples independent of the pH or light/dark conditions (∼230 µg/L or ∼5% of total Ag). In EPS-containing samples, the dissolved Ag concentration increased to 448±35 µg/L (10% to total Ag) in the light and to 358±16 µg/L (8% to total Ag) in the dark.

### Formation of Ag NP from AgNO_3_

**Figure 7:**
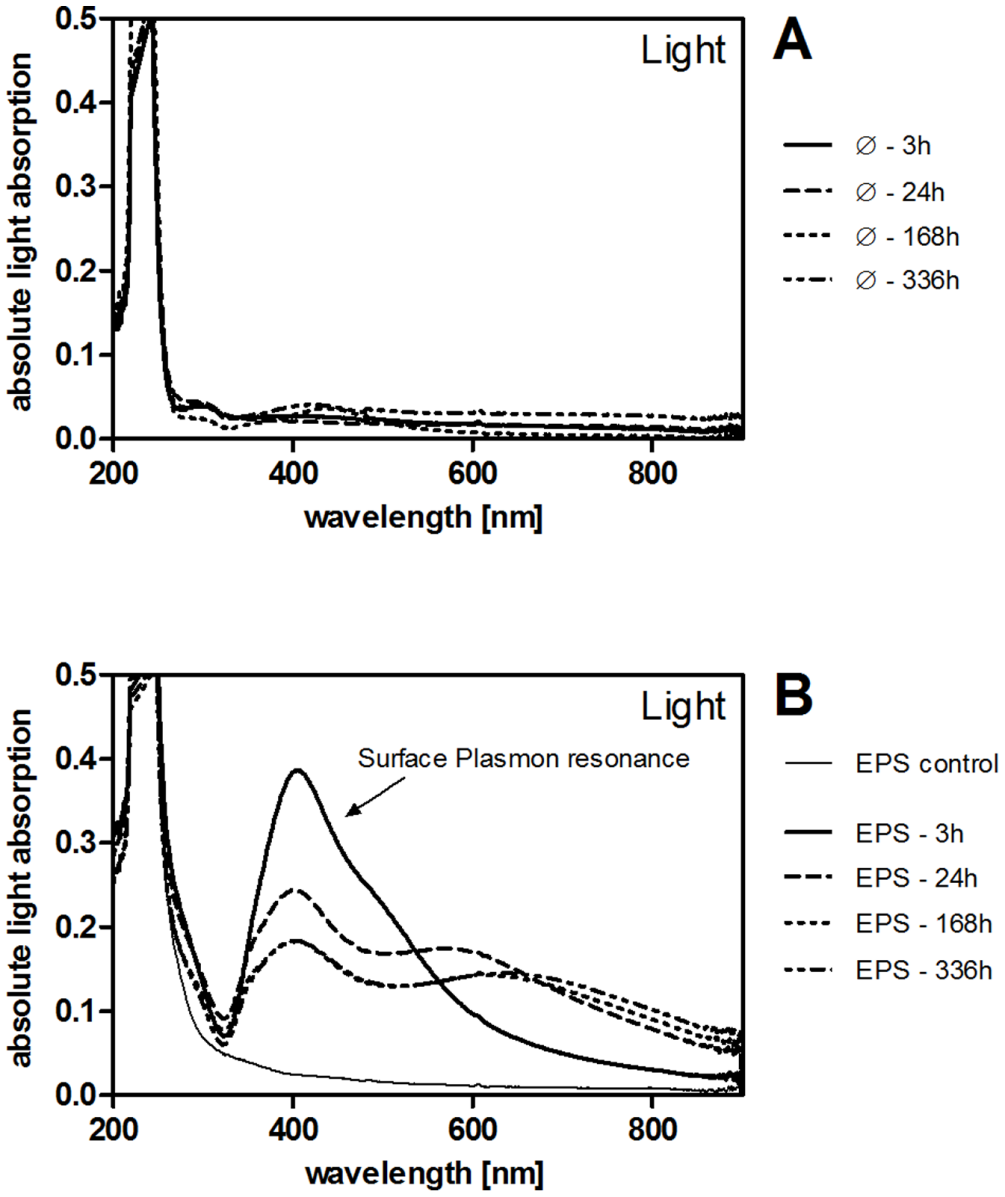
A–B: Spectra of light absorption of AgNO_3_ solutions (5 mg/L Ag) as determined by UV-VIS spectroscopy. Samples were incubated in light (100 µE/m^2^s) at pH 6 without (**A**) or with EPS (**B**, 10 mg DOC/L; 3 h –168 h spectra obtained with EPS extract 1; 336 h spectrum obtained with EPS extract 4). EPS control (**B**) contained 10 mg DOC/L EPS in 2 mM NaHCO_3_. Different EPS extracts did not differ in the absorption of light >300 nm.

AgNO_3_ solutions (5 mg/L Ag) with and without EPS (10 mg DOC/L) were analyzed by DLS, NTA, and UV-VIS. An Ag NP specific SPR signal was observed under all conditions except in the dark without EPS. In AgNO_3_ solutions with EPS extracts 1–3 and 5, a SPR signal (400–420 nm) appeared between 0 h and 3 h after the start of incubation in light (Figure 7 B). After 24 h, a shoulder around 600 nm became prominent which had shifted slightly to longer wavelengths after 168 h. The process was delayed in the presence of EPS extract 4 (low biopolymer concentration, low C/N ratio in the biopolymer fraction). It took up to 336 h for the same light absorption spectrum to develop (Figure 7 B). In EPS-free samples, a small SPR maximum around 440 nm was visible after 168 h incubation in light (Figure 7 A). A similar signal was detected in EPS-containing samples in the dark (Figure S7).

**Figure 8:**
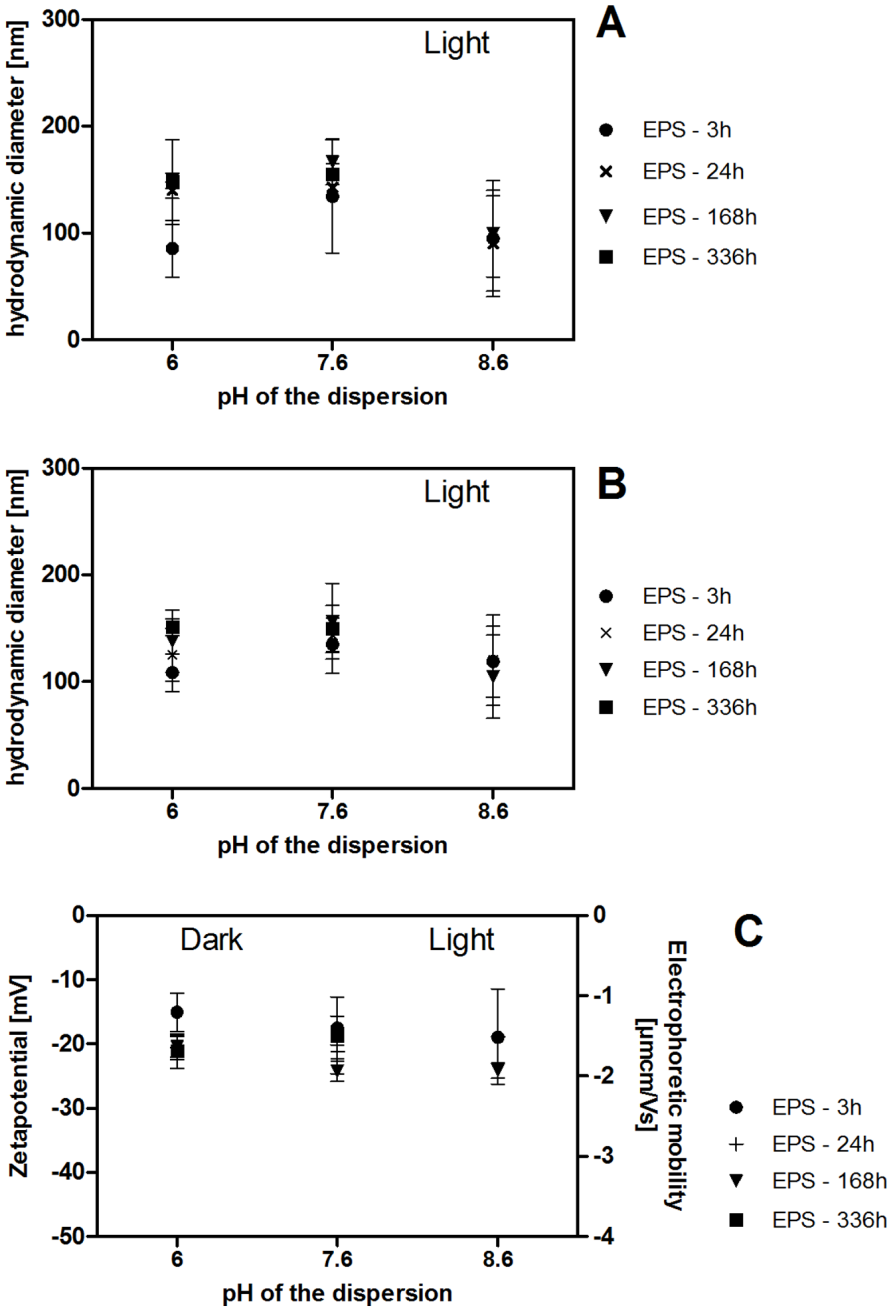
A–C: Mean hydrodynamic diameter of NP formed from AgNO_3_ (5 mg/L dispersions) with standard deviations as determined DLS (intensity distribution (A) and NTA (B). Mean EPM and zetapotential with standard deviations determined by electrophoresis/DLS (C). Samples were incubated in light (100 µE/m^2^s). 3 h: n = 15, 5 independent experiments (EPS extracts 1–5, 10 mg DOC/L). 168 h: n = 12, 4 independent experiments (EPS extracts 1–3, 5). 336 h: n = 3, 1 experiment (EPS extract 4).

Due to a very low or insufficient signal intensity, we could not acquire DLS and NTA data for AgNO_3_ solutions that were light-treated without EPS and dark-treated with EPS. The mean hydrodynamic diameter of NP detected in AgNO_3_ solutions with EPS in the light was 86–195 nm (DLS) and 105–185 nm (NTA) (Figure 8 A–B, Tables S13, S14). While there was no significant time dependence, an increase in pH to pH 8.6 resulted in smaller mean hydrodynamic diameters. EPM of the NP formed decreased over time from −1.12±0.23 µmcm/Vs (pH 6) at 3 h to −1.6±0.13 µmcm/Vs (pH 6) at 168 h (Figure 8 C, Tables S13, S14).

Results for 0.5 mg/L Ag in AgNO_3_ solutions showed the same trends regarding mean sizes and EPM as solutions containing 5 mg/L Ag NO_3_ (Tables S13, S14).

### Dissolved Ag^+^ in AgNO_3_ solutions

The mean concentration of dissolved Ag in AgNO_3_ solutions with and without EPS was 4414±201 µg/L directly after the start of the experiments and was equal to the amount of total Ag detected. The concentration of dissolved Ag remained constant over 168 h (4435±304 µg/L) except in EPS-containing samples at pH 6 and 7.6 incubated in light (Table 2). Here, the concentration of dissolved Ag decreased to 2470±280 µg/L (pH 6) and 3575±333 µg/L (pH 7.6), respectively. This corresponds to a decrease by 43% and 17%, respectively, compared to the measured total Ag concentration.

**Table 2:** Average concentrations of total and dissolved Ag with standard deviations (SD) in AgNO_3_ dispersions as determined by ICP-MS of acid-digested samples incubated in light (100 µE/m^2^s) with (+) or without (−) EPS (10 mg DOC/L) for 168 h.

		[Ag] µg/L			
EPS	pH	Total Ag	SD	Dissolved Ag	SD
−	6	4344	±279	4430	±150
−	7.6	4234	±70	4580	±162
−	8.6	4265	±113	4593	±78
+	6	4314	±173	2470	±280
+	7.6	4259	±44	3575	±333
+	8.6	4280	±42	4241	±129

n = 24 from 3 independent experiments.

XAS and EDX analyses of selected samples showed that NP formed from AgNO_3_ with EPS were dominantly metallic Ag (Figure S5E, S5F, and S6, Table S5).

## Discussion

### EPS characterization

We hypothesized that the interaction of engineered NP with EPS would among others be determined by the composition of EPS which depends on the biofilm community composition. We thus analyzed EPS extracted from periphyton with a focus on inter-community variability of EPS composition. All EPS extracts showed a similar size distribution of DOC indicating an overall constancy in the type of EPS components. Ratios of the individual groups of constituents varied within one order of magnitude indicating a certain community-dependent variability.

Previously, an overall decrease in EPS C/N ratios has been attributed to an increase of protein [42]. Here, the overall ratio of cDOC to N and the amount of measured proteins did not correlate indicating a sample specific composition of nitrogen containing organic compounds. The biopolymer fraction of EPS mostly contains polysaccharides and proteins. A decrease in the C/N ratio in the biopolymer fraction is thus indicative of the ratio of polysaccharides to proteins [30]. Consequently, the concentration of polysaccharides is higher in EPS extracts with a higher C/N ratio in the biopolymer fraction and a higher percentage of biopolymers. Of the five EPS extracts studied here, extract 4 is thus expected to contain the lowest concentration of polysaccharides. The overall similarity as well as the difference in protein and polysaccharide concentration of the five extracts corroborate the results on the influence of EPS on CeO_2_ and Ag NP and AgNO_3_ as discussed below.

None of the samples showed a UV signal typical of humic substances at 45 min retention time (Figure S3) indicating that no HS were present [41]. This fact is important to distinguish the interaction of EPS extracts with NP in the present study from reports on the interaction of HS on NP stability [43], [44].

### Comparison of DLS and NTA results

DLS and NTA analyses resulted in different mean hydrodynamic diameters which is expected due to the different signals used to derive the particle sizes. The DLS derived z-average represents an intensity based mean size distribution while the mean diameter derived from NTA is based on the number of particle tracks acquired. In polydisperse samples, DLS derived average hydrodynamic diameters are expected to be larger than those derived from NTA due to the strong size dependence of the light scattering, especially for particles <100 nm. This probably explains the smaller average diameters obtained by NTA for CeO_2_ dispersions compared to those obtained by DLS measurements.

However, mean diameters of AgNP dispersions below <100 nm determined by NTA were larger than those determined by DLS, whereas those >100 nm were larger according to DLS. Tables S9, S11, and S13 list mean and mode of the size distribution determined by NTA next to z-averages determined by DLS. The difference in mean and mode is indicative of the polydispersity and non-normal size-distribution of the samples. We used NTA mean values to compare to DLS z-averages in the manuscript. The less polydisperse the samples are, the closer are z-average (DLS) and mean diameter (NTA). Consequently, the higher the polydispersity the stronger are the results influenced by the different biases of the methods [45].

### Stability and dissolution of CeO_2_ NP

Electron microscopy investigations revealed that primary particles (25 nm, Figure 2) were mostly aggregated as typically observed for particles synthesized by flame pyrolysis.

Average hydrodynamic diameters determined by DLS (∼200–600 nm) and mean sizes measured by NTA (∼200 nm) indicated that the aggregates detected by TEM remained stable over time independent of the experimental condition (pH, light/dark). As the primary particles were strongly sintered together, EPS did not disperse the aggregates into single primary particles and the average particle size remained unchanged. This observation is supported by the fact that the total Ce recovered from CeO_2_ NP dispersions as determined by ICP-MS remained stable over time. The amount of suspended CeO_2_ NP was thus constant.

The decreasing EPM within one week of light exposure in the presence of NaHCO_3_ as with EPS also indicates a stabilization of CeO_2_ NP. The decrease was not influenced by pH. According to speciation data modeled with Visual MINTEQ software, the major carbonate species were H_2_CO_3_* (aq) and HCO_3_^−^ (68% and 32%) at pH 6 and HCO_3_^−^ (95%/97%) at pH 7.6/8 (see table S3). These calculations suggest comparable effects of different carbonate species and negatively charged EPS components on the surface charge of CeO_2_ NP. The negative EPM observed in this study are in line with the expected negative charge of EPS components at the pH conditions applied here. The negative charge is due to polysaccharide residues such as glucose, xylose, fucose, galactose, and uronic acids [29], [46] and the presence of low M_r_ acids [41]. Stabilization of CeO_2_ NP by artificial mixes of natural organic matter (NOM) has been reported previously [47]. As in our study which used a natural mix of NOM, mean zetapotentials (derived from EPM by the same model) were −21.8 mV to −55.8 mV corresponding to the overall negative charge of the organic molecules used.

We observed an increase in dissolved Ce over time. This may be explained by a redox-cycling of Ce atoms between Ce^III^ and Ce^IV^ on the surface of CeO_2_ NP [23]. In contrast to Ce^III^, Ce^IV^ is insoluble in aqueous environments [48]. Thus, the dissolved Ce was either already present as Ce^III^ and directly released from the NP surfaces or was released following reduction of Ce^IV^ to Ce^III^. The amount of dissolved Ce was significantly larger at pH 6 than at pH 7.6 and 8.6. A higher Ce^III^/Ce^IV^ ratio at lower pH is expected according to the pH-dependence of the reduction reaction [49]. An increase of Ce^III^ with a decrease in pH from 8.6 to 6 was found in perchlorate solution [49] and in natural sea water in the dark where the oxidation rate of Ce^III^ to Ce^IV^ increased between pH 7 to 7.5 but did not further increase between pH 7.5 and 8.6 [50]. Furthermore, the higher concentration of H^+^ at pH 6 may lead to a competition between H^+^ and Ce^III^ on the surface of CeO_2_ NP and thus a more efficient desorption of Ce^III^.

We found that the amount of dissolved Ce decreased in the presence of EPS. Light further reduced the concentration of dissolved Ce in the presence EPS while there was no significant difference between light/dark treated samples without EPS. The amount of free Ce^III^ at pH 6 was thus decreased in the presence of EPS as opposed to NaHCO_3_ only. According to the above discussed hypothesis, this may be explained by a lower Ce^III^/Ce^IV^ ratio following oxidation of Ce^III^ to Ce^IV^. This is possibly due to two parallel processes. On the one hand, Ce^III^ ions on CeO_2_ surfaces are potential sites for catalysis and photooxidation and -reduction [51]. Thus, Ce^III^ ions on the CeO_2_ NP studied here may have reduced EPS components enhanced by photocatalysis which reduced the amount of Ce^III^ and thus the concentration of dissolved Ce. On the other hand, EPS components may have formed ROS in the light which oxidized Ce^III^ resulting in a lower amount of dissolved Ce^III^. These processes would be in line with the observation that artificial mixes of NOM alone decreased the fraction of Ce^III^ in CeO_2_ NP at neutral pH [52], [53] and induced ROS in the light which then oxidized metal ions [54].

### Stability, dissolution and *de novo* formation of Ag NP

Ag NP were in the same size range when dispersed in NaHCO_3_ as reported previously (40–50 nm (DLS), pH 6–10 [10]) and remained stable independent of pH in the range of pH 6 to pH 8.6 and light/dark conditions. Similarly, EPS did not impact the stability of Ag NP dispersions in the dark. However, when EPS-containing Ag NP dispersions were exposed to light, Ag NP mean diameter as determined by DLS and NTA first increased in size with decreasing pH and then remained stable. In the presence of EPS, EPM were similar to those of EPS-treated CeO_2_ NP dispersions indicating an interaction of EPS components with Ag NP despite the negative charge of both Ag NP and EPS. Previously, PVP-stabilized Ag NP were found to aggregate in the presence of EPS extracted from a single bacterial strain [33], while other Ag NP (unknown surface modification) were stabilized by bacterial EPS [55]. In both cases, pH and light/dark dependency of the processes were not assessed. Nevertheless, our results and the existing reports indicate that Ag NP surface modifications will influence the effect of different types of EPS on NP stability.

SPR spectra indicate that the increase in Ag NP average diameter in our study (pH 6 with EPS in light) was both due to the formation of stable NP agglomerates and an increase in individual NP diameter. In particular, SPR spectra showed a decrease in light absorption at the initial peak position along with a second maximum at higher wavelengths. The observed change in light absorbance of Ag NP dispersions results from agglomeration as well as an increase in individual particle size [56]. This observation corresponds to the increase in mean hydrodynamic diameter detected by DLS and NTA. TEM images of selected samples qualitatively support these data.

An increase in particle diameter will change the diffusion of Ag NP in periphyton as has been shown for heterotrophic biofilms [57] and thus the exposure of periphytic organisms.

As dominantly metallic Ag was detected by XAS and EDX in NP dispersions incubated with EPS at pH 6 in the light, we hypothesized that the underlying mechanism was pH-dependent and that EPS-enhanced photoreduction of Ag^+^ on Ag NP surfaces was occurring. Previous studies have reported on interaction of Ag^+^ with NOM [58] and Ag NP formation from Ag^+^ in the presence of NOM (humic acid [44], humic and fulvic acids [43]) and whole cell extracts (marine algae [59], soy bean [60]), thus the formation of Ag NP from extracellular substances can be expected. Photoreduction of Ag^+^ to metallic Ag in particular was observed in the presence of NOM [61] and without an additional reducing agent (reviewed by [62]). Formation of Ag_2_S is not expected under these conditions, because all experiments were carried out in the presence of oxygen and there were no obvious sources of sulfide.

Experiments assessing the independent and combined effects of EPS, light and pH with AgNO_3_ as a source of Ag^+^ confirmed this hypothesis. We observed an Ag NP specific SPR signal under all conditions except in the dark without EPS and detected NP by DLS and NTA in AgNO_3_ solutions with EPS in the light. However, the efficiency of NP formation was low in the light without EPS and in the dark with EPS resulting in a NP concentration too low for analysis by DLS and NTA. These results indicate that light and EPS alone reduce Ag^+^ to metallic Ag inducing Ag NP formation and that a combination of both increases the reduction rate. This behavior is in contrast to the system CeO_2_– EPS, where EPS seem to act as oxidizing agent. Effects of EPS to Ag^+^/Ag°-NP and to Ce^IV^O_2_/Ce^III^ are in line with the different redox potential of these redox couples. Despite the size increase of individual Ag particles in the presence of EPS and light, the concentration of Ag^+^ increases over time similar to EPS-free samples in the light/dark and to EPS-containing samples in the dark. Consequently, Ag NP must be oxidized to release Ag^+^ parallel to photoreduction. Under supersaturated conditions, colloidal systems stabilize at a system-dependent size distribution steered by a process called Ostwald-ripening. The process is based on the reduction of energy in the system. Small crystals dissolve driven by their high surface tension, while large crystals grow due to supersaturation of the solution and less surface tension to counteract growth. This process has been described for Ag NP synthesis [63]. In our system, the concentration of Ag^+^ increases over time at all experimental conditions while the size-distribution only shifts to larger NP sizes in the presence of EPS and light in a pH-dependent manner. Reduction of Ag^+^ to Ag° is only possible if a reductant is present (here the natural EPS components) and is more efficient under light conditions. Under these conditions the formation of Ag° at the surface of the larger Ag NP then leads to increased size.

The difference in the rate of Ag NP size increase and Ag NP formation between EPS extract 4 and 1–3/5 must be due to the different composition of EPS as there was no difference in dissolved Ag^+^ in the presence of the different extracts. In a previous study, the formation of Ag nanoplates by algae extracts was explained by proteins reducing Ag^+^ and controlling the shape of the nanomaterial with carboxyl groups and hydroxyl groups potentially being responsible for Ag^+^ reduction [64]. However, polysaccharides which contain hydroxyl groups as well as hemiacetal ends are expected to be more reactive for reduction of Ag^+^ [65]. We found that the extract that induced a slower increase in size and formation of Ag NP had the highest protein content, less high M_r_ biopolymers and low M_r_ acids and the lowest concentration of polysaccharides in the biopolymer fraction. This suggests that acidic groups of high M_r_ polysaccharides and/or low M_r_ acids in the EPS extracts were mainly involved in the reduction of Ag^+^.

As the total Ag concentration remained stable in AgNO_3_ solutions in which Ag NP formation had occurred, the decrease in dissolved Ag can be explained by the formation of Ag NP. As DLS and NTA cannot be used to quantify particle number, we used a combination of the NP diameter range determined by DLS and NTA and the decrease in dissolved Ag to estimate the number of particles formed. Based on an average NP diameter of 100 nm –200 nm, roughly 3.6×10^11^–4.5×10^1^° NP/L were formed at pH 6 and roughly 1.8×10^11^ −2.2×10^1^° NP/L were formed at pH 7.6. As samples incubated at pH 8.6 produced a DLS signal and contained NP according to NTA, a decrease in dissolved Ag^+^ could be expected. However, the average DLS signal counts (Table S7) indicate only a small amount of Ag NP at pH 8.6. The corresponding decrease in dissolved concentration may thus fall in the range of the standard deviation of the ICP-MS measurements. In summary, the formation of Ag NP was most efficient at pH 6. Furthermore, the size increase of Ag NP was largest at pH 6. Consequently, the reduction of Ag^+^ to Ag was most efficient at the lowest pH studied.

The reactive components of EPS that probably contributed to EPS-enhanced photoreduction (M_r_ biopolymers, especially polysaccharides, and low M_r_ organic acids) are expected to be more protonated at pH 6 compared to pH 7.6/8.6 resulting into a less negative charge. The more efficient reduction of Ag^+^ and growth of Ag NP at lower pH seems counterintuitive. Less negative charge on relevant functional groups of EPS components at pH 6 may have resulted in a weaker interaction with Ag^+^, and thus a less efficient detachment of Ag^+^ ions from Ag NP surfaces, and a more efficient photoreduction and formation of Ag° at the NP surface. In the case of Ag NP formation from AgNO_3_, a weaker interaction of Ag^+^ with EPS components may also have facilitated the reduction to elemental Ag and Ag NP formation. Furthermore, the carbonate coating of the Ag NP is less negatively charged at lower pH, so that interactions with the EPS may be facilitated. At the same time, the interaction of EPS components, Ag NP and Ag^+^ is probably more dynamic at pH 6 as both reduction and oxidation of Ag^+^ will occur simultaneously, so that released Ag+ may be reduced again and lead to growth of the Ag NP. A pH-dependent process with an inverse relationship as described here was found for humic and fulvic acids which induced Ag NP formation from Ag^+^ with an increase in pH (6.1 to 9) accelerating the formation of NP [66]. On the other hand, the release of Ag^+^ from citrate-stabilized Ag NP in the presence of humic acids increased with increasing pH [67]. The processes of AgNP formation and growth are thus dependent on the pH dependence of the functional groups of EPS, of the surface coating of AgNP and of the reduction reactions.

### Environmental relevance

In this study we present results from basic research on the interaction of selected engineered NP with EPS under controlled conditions. pH, light intensity, and the buffer system were selected according to their relevance to freshwater periphyton. The chemical make-up of stream water, natural colloids, and DOC other than EPS were not included to be able to analyze the specific effects of EPS.

Despite differences in relative chemical composition, the five EPS extracts studied had the same effect on CeO_2_ NP stability and release of Ce^III^ as well as on photoreduction of Ag^+^ resulting in pH-dependent Ag NP size increase and NP formation from AgNO_3_. Merely the kinetics of Ag^+^ reduction were slower in the presence of the EPS extract that probably contained the lowest concentration of polysaccharides as part of the biopolymer fraction and the lowest concentration of low M_r_ acids. These results indicate that EPS contains reactive biomolecules that may be decisive regarding the fate of Ag NP and Ag^+^ in periphyton.

The similarity of newly formed and engineered Ag NP observed in this study demonstrates that it will be challenging to distinguish between both particle types in environmental samples. Knowing the origin of Ag NP is crucial to understanding sources of Ag species in freshwater systems, to tracking engineered Ag NP and to understanding potentially variable effects of Ag in the environment.

## Conclusions

CeO_2_ NP were stabilized by EPS extracted from periphyton and dissolved to a certain extent with decreasing pH and with time. Ag NP were likewise stabilized by EPS and increased in size with decreasing pH by EPS-enhanced photoreduction. EPS also enhanced the formation of Ag NP from AgNO_3_ with a decrease in pH increasing the efficiency of *de novo* NP formation. The composition of the EPS extracts regarding biopolymers and possibly low M_r_ acids determined the kinetics of the photoreduction. The parameters tested in this study, namely photon flux, pH, and EPS change with light irradiation, the photosynthetic and respiratory activity of periphytic organisms, and with species composition. We conclude that local conditions in periphyton will influence the properties of the tested types of nanomaterials and thus the exposure of periphytic organisms.

## Supporting Information

Figure S1 **Water temperature in the channel used for periphyton colonization measured by a HOBO Pendant Temperature/Light Data Logger.** Blue numbers indicate the EPS extract produced at the end of the respective period. Blue lines indicate the mean water temperature during the respective period.
(TIF)

Figure S2 **Organic carbon (OC) signal chromatograms of EPS extracts 1–5 as determined by LC-OCD-OND.** Samples were diluted 1∶50 with nanopure water (Ω 18, Milli-Q).
(TIF)

Figure S3 **UV signal (254 nm) chromatograms of EPS extracts 1–5 as determined by LC-OCD-OND.** Samples were diluted 1∶50 with nanopure water (Ω 18, Milli-Q).
(TIF)

Figure S4 **OC chromatograms of two extracts obtained independently by two individuals on the same day from periphyton from 32 slides from two different channels after three weeks of colonization.**
(TIF)

Figure S5 ***A*** TEM bright field image of Ag NP, ***B*** TEM bright field image of Ag NP exposed to EPS for 24 h (pH 6, light), ***C*** High angular annular dark field (HAADF) image and ***D*** Energy dispersive x-ray (EDX) spectrum of Ag NP exposed to EPS for 24 h (pH 6, light); ***E*** HAADF image and ***F*** EDX spectrum of AgNO_3_ exposed to EPS for 24 h (pH6, light).
(TIF)

Figure S6 **XANES(left) and EXAFS (right) spectra of two experimental samples (red) and the reference materials (black) used for the linear combination fitting (Table S5).**
(TIF)

Figure S7 **Spectra of light absorption of AgNO_3_ solutions (5 mg/L Ag) as determined by UV-VIS spectroscopy.** Samples were incubated in the dark at pH 6 with EPS (10 mg DOC/L; spectra obtained with EPS extract 1) EPS control contained 10 mg DOC/L EPS in 2 mM NaHCO_3_. Different EPS extracts did not differ in the absorption of light >300 nm.
(TIF)

Figure S8 **Representative DLS spectra of CeO_2_ NPs in 2 mM NaHCO_3_ without EPS after 168 h of incubation in light.** A: 5 mg Ce/L CeO_2_ NP, B: 0.5 mg Ce/L CeO_2_ NP.
(TIF)

Table S1 NP properties.
(PDF)

Table S2 Chriesbach water chemistry.
(PDF)

Table S3 Speciation of the components of Ag NP dispersions and AgNO_3_ solutions according to Visual MINTEQ modelling software. NO_3_^−^ was 99.95% dissolved as NO_3_^−^ and 0.05% NaNO_3_ (aq) in all cases.
(PDF)

Table S4 Sampling scheme for NP characterization. 1 DLS, 2 NTA, 3 UV-VIS, 4 ICP-MS^$^. L : light, D : dark; ^$^ only performed for 5 mg/L dispersions; * only for EPS extract 4 and corresponding controls
(PDF)

Table S5 Fractions of Ag(0), Ag_2_O and Ag_2_S derived from LCF results of XANES and EXAFS spectra from experimental samples. The XANES spectra were fitted from 24490 to 26000 eV and the EXAFS from 3–10k. The individual fractions were constrained to range between 0 and 1 and the sum of the fitted fractions was left unconstrained (XANES) or was constraint to 1 (EXAFS).
(PDF)

Table S6 Average derived DLS count rates (kilocounts per second, kps) with standard deviations of Ag NP dispersions in 2 mM NaHCO_3_ without EPS after 168 h of incubation in light.
(PDF)

Table S7 Average derived DLS count rates (kilocounts per second, kps) with standard deviations and estimated NP concentration in samples containing EPS and AgNO_3_ (5 mg Ag/L) incubated at pH 6, 7.6, or 8.6 in the light for 168 h.
(PDF)

Table S8 Average derived DLS count rates (kilocounts per second, kps) with standard deviations of CeO_2_ NP dispersions in 2 mM NaHCO_3_ without EPS after 168 h of incubation in light.
(PDF)

Table S9 Z-averages (DLS), polydispersity (PDI), mode and mean diameters (NTA), zetapotential, and EPM of CeO_2_ NP dispersions dependent on pH, light/dark, EPS content, and time. Each value is derived from three measurements of three replicates (3x 3n). Blue: data used to calculate mean values represented in Figures 1 A–D.
(PDF)

Table S10 Standard deviations of Z-averages (DLS), polydispersity (PDI), mode and mean diameters (NTA), zetapotential, and EPM of CeO_2_ NP dispersions dependent on pH, light/dark, EPS content, and time.
(PDF)

Table S11 Mean Z-averages (DLS), polydispersity (PDI), mode and mean diameters (NTA), zetapotential, and EPM of Ag NP dispersions dependent on pH, light/dark, EPS content, and time. Each value is derived from three measurements of three replicates (3x 3n). Blue: data used to calculate mean values represented in Figures 4 A–D.
(PDF)

Table S12 Standard deviations of Z-averages (DLS), polydispersity (PDI), mode and mean diameters (NTA), zetapotential, and EPM of Ag NP dispersions dependent on pH, light/dark, EPS content, and time.
(PDF)

Table S13 Z-averages (DLS), polydispersity (PDI), mode and mean diameters (NTA), zetapotential, and EPM of NP formed in AgNO_3_ solutions dependent on pH, light/dark, EPS content, and time. ns: no signal. Each value is derived from three measurements of three replicates (3x 3n). blue: data used to calculate mean values represented in Figures 8 A–C.
(PDF)

Table S14 Standard deviations of Z-averages (DLS), polydispersity (PDI), mode and mean diameters (NTA), zetapotential, and EPM of NP formed in AgNO_3_ solutions dependent on pH, light/dark, EPS content, and time.
(PDF)

Text S1 Settings for DLS and NTA measurements.
(PDF)
