# Supplementary figures and images for: Extracellular Polymeric Substances (EPS) of Freshwater Biofilms Stabilize and Modify CeO2 and Ag Nanoparticles
Source: PLoS One. 2014 Oct 21;9(10):e110709. doi: 10.1371/journal.pone.0110709 (PMC4204993; doi:10.1371/journal.pone.0110709)

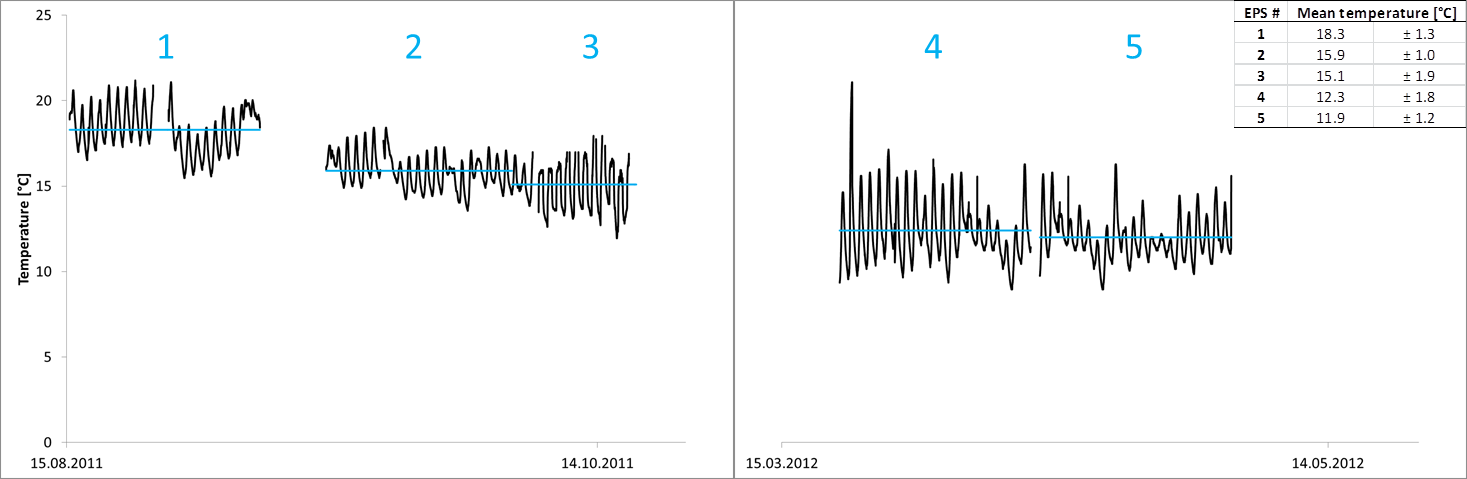

Supplement: Figure S1 — Water temperature in the channel used for periphyton colonization measured by a HOBO Pendant Temperature/Light Data Logger. Blue numbers indicate the EPS extract produced at the end of the respective period. Blue lines indicate the mean water temperature during the respective period. (TIF) [file pone.0110709.s001.tif]

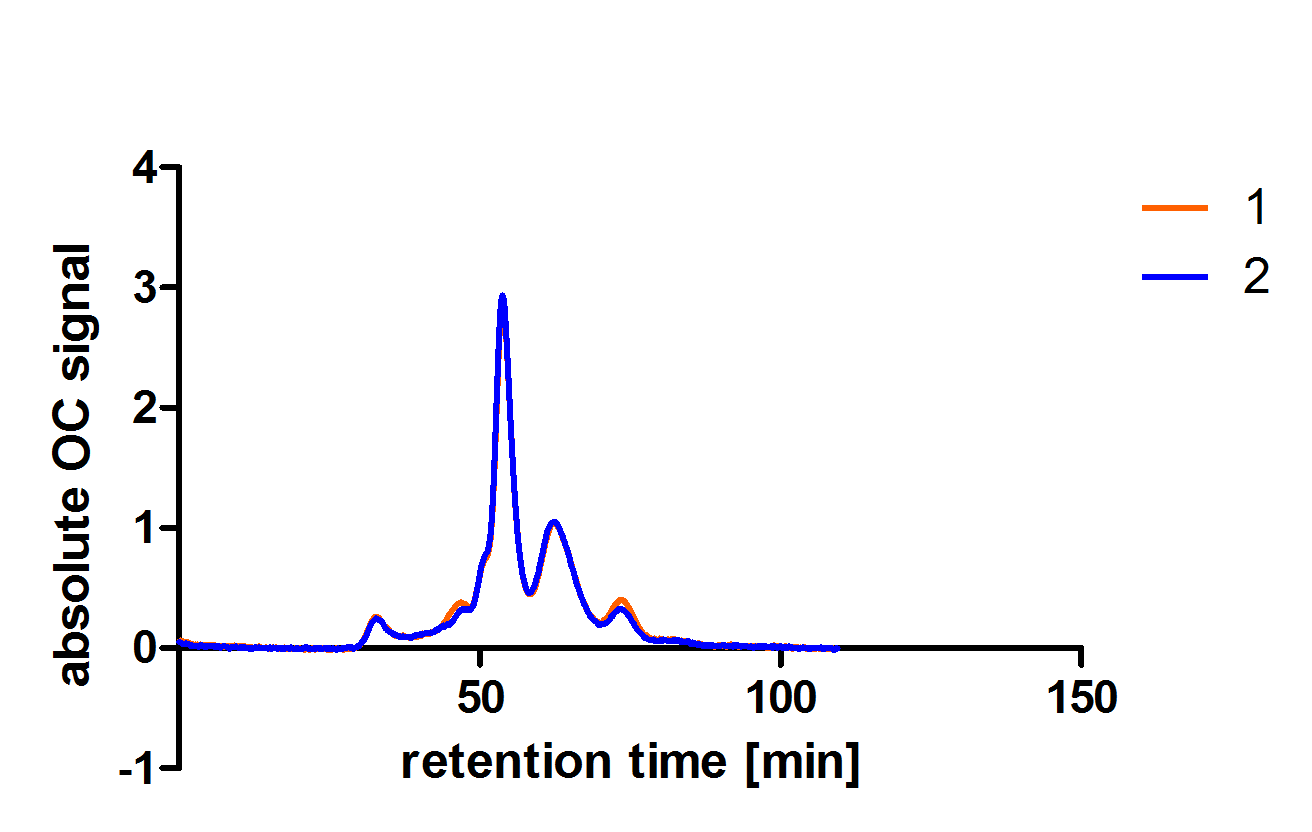

Supplement: Figure S4 — OC chromatograms of two extracts obtained independently by two individuals on the same day from periphyton from 32 slides from two different channels after three weeks of colonization. (TIF) [file pone.0110709.s004.tif]

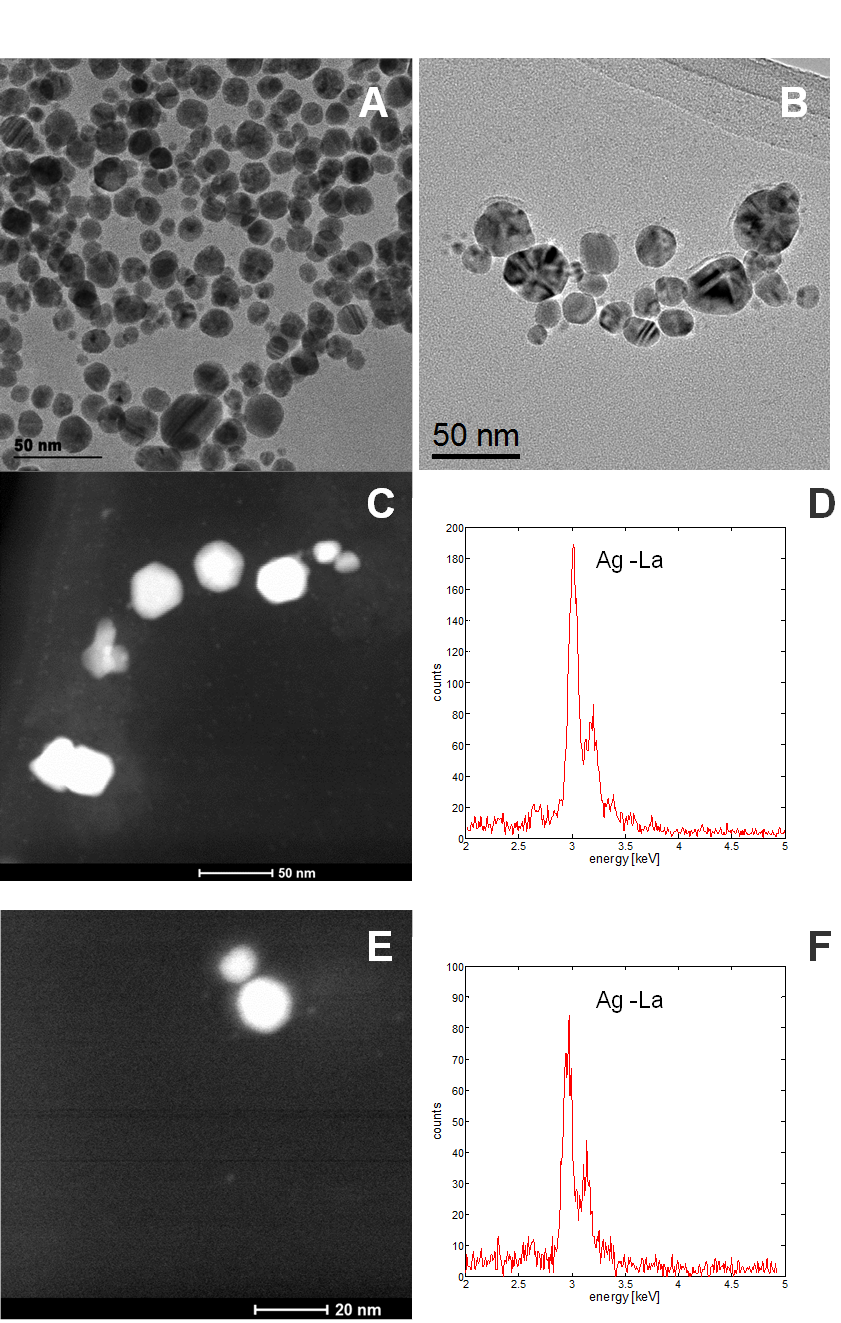

Supplement: Figure S5 — A TEM bright field image of Ag NP, B TEM bright field image of Ag NP exposed to EPS for 24 h (pH 6, light), C High angular annular dark field (HAADF) image and D Energy dispersive x-ray (EDX) spectrum of Ag NP exposed to EPS for 24 h (pH 6, light); E HAADF image and F EDX spectrum of AgNO3 exposed to EPS for 24 h (pH6, light). (TIF) [file pone.0110709.s005.tif]

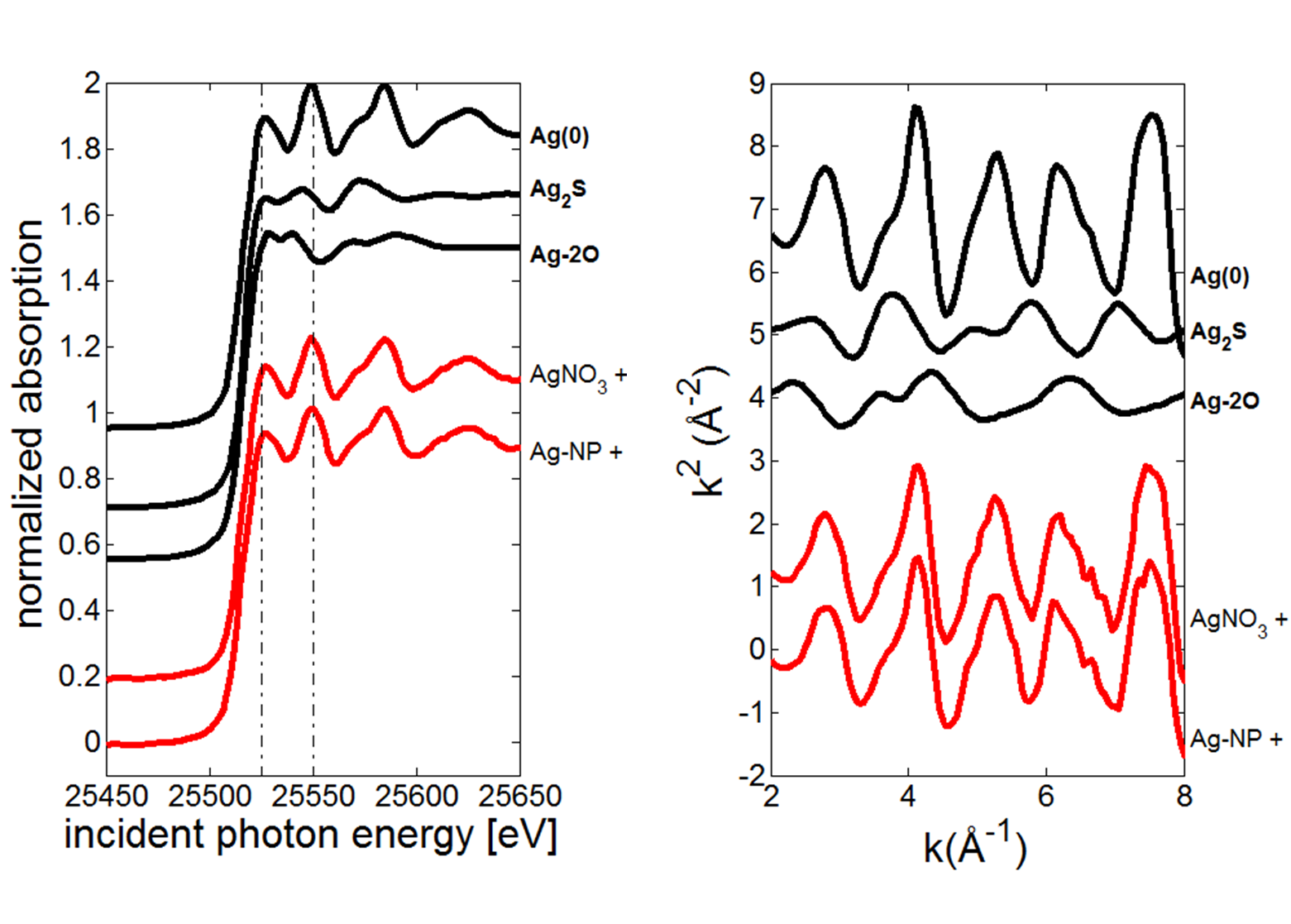

Supplement: Figure S6 — XANES(left) and EXAFS (right) spectra of two experimental samples (red) and the reference materials (black) used for the linear combination fitting (Table S5). (TIF) [file pone.0110709.s006.tif]

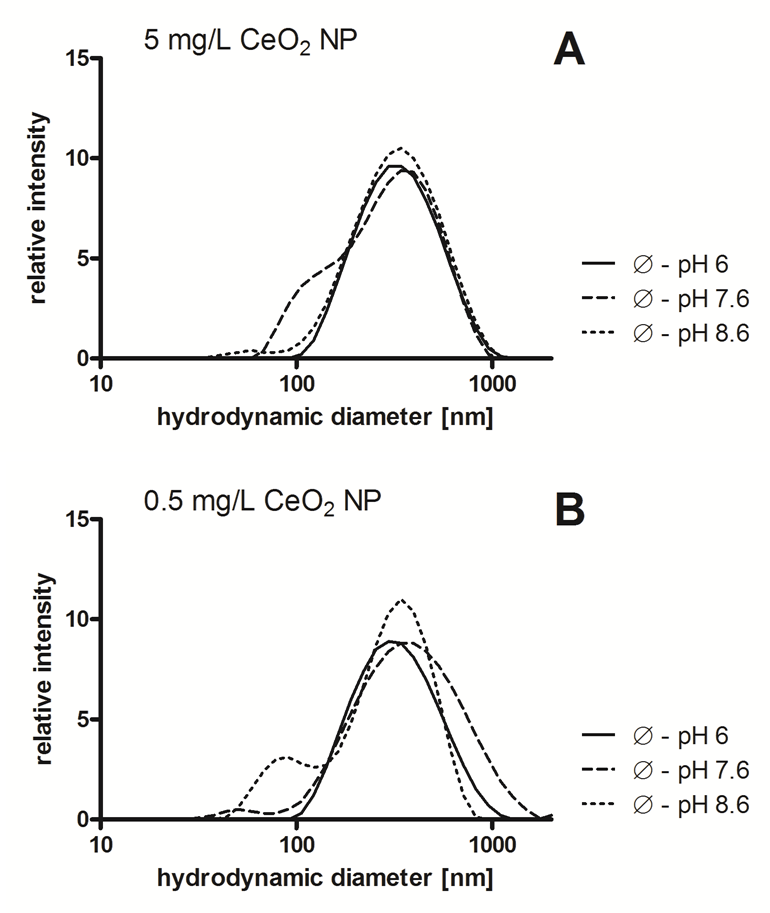

Supplement: Figure S8 — Representative DLS spectra of CeO2 NPs in 2 mM NaHCO3 without EPS after 168 h of incubation in light. A: 5 mg Ce/L CeO2 NP, B: 0.5 mg Ce/L CeO2 NP. (TIF) [file pone.0110709.s008.tif]
