# Supplementary material for: Extracellular Polymeric Substances (EPS) of Freshwater Biofilms Stabilize and Modify CeO2 and Ag Nanoparticles
Source: PLoS One. 2014 Oct 21;9(10):e110709. doi: 10.1371/journal.pone.0110709 (PMC4204993; doi:10.1371/journal.pone.0110709)
Supplement: Table S1 — NP properties. (PDF) [file pone.0110709.s009.pdf]

	Provider	Synthesis method	Stabilizer	Primary particle size (TEM)	Hydrodynamic diameter in nanopure H_2O (pH 7.2, DLS)	Zetapotential in nanopure H_2O (pH 7.2)
**AgNPs [1]**	NanoSys GmbH (Wolfhalden, Switzerland)	Wet precipitation	Carbonate	25 nm	40 nm	-42 mV
**CeO_2 NPs**	Nanograde AG (Staefa, Switzerland)	Flame-spray	-	25 nm	Not stable	Not stable

1. Navarro E, Piccapietra F, Wagner B, Marconi F, Kaegi R, et al. (2008) Toxicity of silver nanoparticles to Chlamydomonas reinhardtii. Environ Sci Technol 42: 8959-8964.
