# Supplementary material for: Extracellular Polymeric Substances (EPS) of Freshwater Biofilms Stabilize and Modify CeO2 and Ag Nanoparticles
Source: PLoS One. 2014 Oct 21;9(10):e110709. doi: 10.1371/journal.pone.0110709 (PMC4204993; doi:10.1371/journal.pone.0110709)
Supplement: Table S2 — Chriesbach water chemistry. (PDF) [file pone.0110709.s010.pdf]

	DOC [mg/L]	NH_4 [µg N/L]	NO_2 [µg N/L]	NO_3 [mg N/L]	total P [µg P/L]
**11.04.2012[+]**	4.7	202	118	7.2	355
**02.05.2012[+]**	2.3	27.9	26.6	6.4	140
**2010-2011***	3.3	50	30	6.8	130
**2012-2013***	2.7	50	28	6.3	110

[+]measured on campus (coordinates 47.404827, 8.611653)

*measured by AWEL, Amt für Abfall, Wasser, Energie und Luft (coordinates 47.402908, 8.604966)

A mean of 15.9% of Chriesbach water is treated sewage water.
