# Supplementary material for: Extracellular Polymeric Substances (EPS) of Freshwater Biofilms Stabilize and Modify CeO2 and Ag Nanoparticles
Source: PLoS One. 2014 Oct 21;9(10):e110709. doi: 10.1371/journal.pone.0110709 (PMC4204993; doi:10.1371/journal.pone.0110709)
Supplement: Table S3 — Speciation of the components of Ag NP dispersions and AgNO3 solutions according to Visual MINTEQ modelling software. NO3 − was 99.95% dissolved as NO3 − and 0.05% NaNO3 (aq) in all cases. (PDF) [file pone.0110709.s011.pdf]

Species	pH 6	pH 7.6	pH 8.6
Ag^+	100	99.996	99.962
AgOH (aq)			0.038
CO_3^{-2}		0.205	2.112
$NaCO_3^-$			0.064
$NaHCO_3$ (aq)	0.029	0.086	0.088
HCO_3^-	31.657	94.618	97.214
H_2CO_3* (aq)	68.312	5.085	0.522
Na^+	99.971	99.908	99.847
$NaCO_3^-$			0.064
$NaHCO_3$ (aq)	0.029	0.086	0.088
