# Supplementary material for: Extracellular Polymeric Substances (EPS) of Freshwater Biofilms Stabilize and Modify CeO2 and Ag Nanoparticles
Source: PLoS One. 2014 Oct 21;9(10):e110709. doi: 10.1371/journal.pone.0110709 (PMC4204993; doi:10.1371/journal.pone.0110709)
Supplement: Table S4 — Sampling scheme for NP characterization. 1 DLS, 2 NTA, 3 UV-VIS, 4 ICP-MS$. L : light, D : dark; $ only performed for 5 mg/L dispersions; * only for EPS extract 4 and corresponding controls (PDF) [file pone.0110709.s012.pdf]

			0 h	3 h	24 h	48 h	168 h	336 h
**AgNPs** **0.5 and 5 mg/L**	**L +/- EPS**	**pH 6**	4	1, 2, 3	1, 2, 3	1*, 2*, 3*	1, 2, 3, 4	1, 2, 3, 4
		**pH 7.6**	4	1, 2, 3	1, 2, 3	1*, 2*, 3*	1, 2, 3, 4	1, 2, 3, 4
		**pH 8.6**	4	1, 2, 3	1, 2, 3	1*, 2*, 3*	1, 2, 3, 4	1, 2, 3, 4
	**D +/- EPS**	**pH 6**	4	1, 2, 3	1, 2, 3	1*, 2*, 3*	1, 2, 3, 4	1, 2, 3, 4
		**pH 7.6**	4	1, 2, 3	1, 2, 3	1*, 2*, 3*	1, 2, 3, 4	1, 2, 3, 4
		**pH 8.6**	4	1, 2, 3	1, 2, 3	1*, 2*, 3*	1, 2, 3, 4	1, 2, 3, 4
**$AgNO_3$** **0.5 and 5 mg Ag/L**	**L +/- EPS**	**pH 6**	4	1, 2, 3	1, 2, 3	1*, 2*, 3*	1, 2, 3, 4	1, 2, 3, 4
		**pH 7.6**	4	1, 2, 3	1, 2, 3	1*, 2*, 3*	1, 2, 3, 4	1, 2, 3, 4
		**pH 8.6**	4	1, 2, 3	1, 2, 3	1*, 2*, 3*	1, 2, 3, 4	1, 2, 3, 4
	**D +/- EPS**	**pH 6**	4	1, 2, 3	1, 2, 3	1*, 2*, 3*	1, 2, 3, 4	1, 2, 3, 4
		**pH 7.6**	4	1, 2, 3	1, 2, 3	1*, 2*, 3*	1, 2, 3, 4	1, 2, 3, 4
		**pH 8.6**	4	1, 2, 3	1, 2, 3	1*, 2*, 3*	1, 2, 3, 4	1, 2, 3, 4
**CeO_2 NPs** **0.5 and 5 mg/L**	**L +/- EPS**	**pH 6**	4	1, 2	1, 2	1*, 2*	1, 2, 4	1, 2, 4
		**pH 7.6**	4	1, 2	1, 2	1*, 2*	1, 2, 4	1, 2, 4
		**pH 8.6**	4	1, 2	1, 2	1*, 2*	1, 2, 4	1, 2, 4
	**D +/- EPS**	**pH 6**	4	1, 2	1, 2	1*, 2*	1, 2, 4	1, 2, 4
		**pH 7.6**	4	1, 2	1, 2	1*, 2*	1, 2, 4	1, 2, 4
		**pH 8.6**	4	1, 2	1, 2	1*, 2*	1, 2, 4	1, 2, 4
