# Supplementary material for: Extracellular Polymeric Substances (EPS) of Freshwater Biofilms Stabilize and Modify CeO2 and Ag Nanoparticles
Source: PLoS One. 2014 Oct 21;9(10):e110709. doi: 10.1371/journal.pone.0110709 (PMC4204993; doi:10.1371/journal.pone.0110709)
Supplement: Table S5 — Fractions of Ag(0), Ag2O and Ag2S derived from LCF results of XANES and EXAFS spectra from experimental samples. The XANES spectra were fitted from 24490 to 26000 eV and the EXAFS from 3–10k. The individual fractions were constrained to range between 0 and 1 and the sum of the fitted fractions was left unconstrained (XANES) or was constraint to 1 (EXAFS). (PDF) [file pone.0110709.s013.pdf]

	Ag(0) (XANES)	Ag_2O (XANES)	Ag_2S (XANES)	R-factor (XANES)	Ag(0) (EXAFS)	Ag_2O (EXAFS)	Ag_2S (EXAFS)	R-factor (EXAFS)
**Ag-NP + EPS**	0.83	0.17	0.00	0.0014	0.80	0.05	0.15	0.0211
**$AgNO_3$ + EPS**	0.90	0.11	0.00	0.0005	0.82	0.06	0.11	0.0291
