# Supplementary material for: Extracellular Polymeric Substances (EPS) of Freshwater Biofilms Stabilize and Modify CeO2 and Ag Nanoparticles
Source: PLoS One. 2014 Oct 21;9(10):e110709. doi: 10.1371/journal.pone.0110709 (PMC4204993; doi:10.1371/journal.pone.0110709)
Supplement: Table S6 — Average derived DLS count rates (kilocounts per second, kps) with standard deviations of Ag NP dispersions in 2 mM NaHCO3 without EPS after 168 h of incubation in light. (PDF) [file pone.0110709.s014.pdf]

	Average derived count rate (kcps)	
**pH**	**5 mg/L**	**0.5 mg/L**
**6**	14854 ± 776	1512 ± 261
**7.6**	14443 ± 913	1524 ± 216
**8.6**	15498 ± 864	1441 ± 257
