# Supplementary material for: Extracellular Polymeric Substances (EPS) of Freshwater Biofilms Stabilize and Modify CeO2 and Ag Nanoparticles
Source: PLoS One. 2014 Oct 21;9(10):e110709. doi: 10.1371/journal.pone.0110709 (PMC4204993; doi:10.1371/journal.pone.0110709)
Supplement: Table S7 — Average derived DLS count rates (kilocounts per second, kps) with standard deviations and estimated NP concentration in samples containing EPS and AgNO3 (5 mg Ag/L) incubated at pH 6, 7.6, or 8.6 in the light for 168 h. (PDF) [file pone.0110709.s015.pdf]

	Average DLS count rates (kcps)	Total Ag^+ [µg/L]	Dissolved Ag^+ [µg/L]	Estimated NP concentration
**pH 6**	39487 ± 2213	4314 ± 173	2470 ± 280	$3.6x10^{11}$ – $4.5x10^{10}$ NP/L
**pH 7.6**	22899 ± 1786	4259 ± 44	3575 ± 333	$1.8x10^{11}$ – 2.2 x 10^{10} NP/L
**pH 8.6**	2742 ± 371	4280 ± 42	4241 ± 129	**-**
