# Supplementary material for: Extracellular Polymeric Substances (EPS) of Freshwater Biofilms Stabilize and Modify CeO2 and Ag Nanoparticles
Source: PLoS One. 2014 Oct 21;9(10):e110709. doi: 10.1371/journal.pone.0110709 (PMC4204993; doi:10.1371/journal.pone.0110709)
Supplement: Table S9 — Z-averages (DLS), polydispersity (PDI), mode and mean diameters (NTA), zetapotential, and EPM of CeO2 NP dispersions dependent on pH, light/dark, EPS content, and time. Each value is derived from three measurements of three replicates (3x 3n). Blue: data used to calculate mean values represented in Figures 1 A–D. (PDF) [file pone.0110709.s017.pdf]

				3 h						24 h						168 h						336 h					
mg/L	EPS	pH	L/D	z-average	PDI	ZP	EPM	Mode	Mean	z-average	PDI	ZP	EPM	Mode	Mean	z-average	PDI	ZP	EPM	Mode	Mean	z-average	PDI	ZP	EPM	Mode	Mean
0.5	1	6	D	274	0.451	-17.1	-1.34	199	222	380	0.425	-19.5	-1.53	198	220	328	0.427	-20.0	-1.57	203	220	327	0.39	-18.1	-1.42	186	158
0.5	2	6	D	290	0.404	-17.0	-1.33	189	256	395	0.389	-20.4	-1.60	239	233	280	0.425	-19.5	-1.53	267	244	398	0.335	-17.7	-1.39	179	170
0.5	3	6	D	277	0.411	-11.8	-0.92	209	215	468	0.424	-11.4	-0.89	248	255	395	0.389	-20.4	-1.60	189	206	386	0.45	-14.8	-1.16	164	174
0.5	4	6	D	268	0.395	-14.1	-1.11	203	237	426	0.392	-13.2	-1.04	211	207	333	0.461	-17.7	-1.39	224	221	339	0.471	-19.7	-1.54	101	131
0.5	5	6	D	288	0.361	-13.1	-1.03	185	219	431	0.393	-15.0	-1.18	188	241	349	0.341	-18.8	-1.47	286	221	266	0.383	-18.9	-1.48	117	140
0.5	1	7.6	D	302	0.378	-16.8	-1.32	203	220	390	0.347	-18.3	-1.43	179	217	340	0.447	-14.3	-1.12	219	248	259	0.337	-16.2	-1.27	120	142
0.5	2	7.6	D	300	0.326	-19.8	-1.55	249	246	352	0.329	-19.3	-1.51	190	216	322	0.329	-15.3	-1.20	198	295	240	0.331	-16.8	-1.32	110	126
0.5	3	7.6	D	314	0.37	-18.7	-1.47	225	222	343	0.32	-16.8	-1.32	238	274	347	0.425	-20.1	-1.58	179	270	245	0.352	-16.7	-1.31	103	126
0.5	4	7.6	D	307	0.329	-13.4	-1.05	187	223	357	0.46	-15.0	-1.18	194	249	407	0.407	-21.4	-1.68	199	274	262	0.417	-21.4	-1.68	122	154
0.5	5	7.6	D	300	0.348	-16.3	-1.28	219	251	385	0.442	-16.2	-1.27	189	215	455	0.448	-22.0	-1.72	257	262	340	0.478	-16.3	-1.28	124	156
0.5	1	8.6	D	418	0.444	-15.8	-1.24	329	301	436	0.461	-19.2	-1.50	192	196	436	0.461	-19.2	-1.50	188	197	293	0.398	-17.6	-1.38	125	151
0.5	2	8.6	D	304	0.308	-22.1	-1.73	264	253	438	0.468	-22.3	-1.75	186	230	429	0.322	-21.4	-1.68	122	178	391	0.361	-17.5	-1.37	166	147
0.5	3	8.6	D	350	0.336	-17.8	-1.40	165	200	315	0.341	-12.0	-0.94	174	272	338	0.468	-22.3	-1.75	139	278	213	0.487	-16.5	-1.29	176	186
0.5	4	8.6	D	344	0.393	-16.7	-1.31	209	225	382	0.37	-15.4	-1.21	218	244	302	0.463	-21.8	-1.71	178	290	210	0.414	-17.6	-1.38	145	215
0.5	5	8.6	D	387	0.432	-14.0	-1.10	169	232	378	0.344	-14.9	-1.17	283	253	395	0.388	-23.2	-1.82	339	271	244	0.484	-20.2	-1.58	146	184
0.5	-	6	D	480	0.346	-16.8	-1.32	137	197	478	0.429	-19.4	-1.52	129	223	378	0.4	-18.4	-1.44	263	193	394	0.364	-14.5	-1.14	147	150
0.5	-	6	D	574	0.489	-12.9	-1.01	233	232	445	0.375	-24.4	-1.91	131	204	345	0.39	-14.4	-1.13	154	213	300	0.321	-15.8	-1.24	223	223
0.5	-	6	D	618	0.445	-16.4	-1.29	267	256	431	0.458	-26.2	-2.05	194	206	331	0.368	-20.0	-1.57	154	170	261	0.404	-15.5	-1.21	122	218
0.5	-	6	D	642	0.479	-15.4	-1.21	119	142	328	0.342	-13.0	-1.02	212	219	303	0.488	-12.8	-1.00	139	153	372	0.37	-18.9	-1.48	144	180
0.5	-	6	D	496	0.475	-14.0	-1.10	139	199	322	0.496	-19.9	-1.56	219	232	398	0.484	-22.7	-1.78	249	264	351	0.365	-16.7	-1.31	117	156
0.5	-	7.6	D	752	0.405	-13.1	-1.03	61	138	319	0.343	-22.2	-1.74	127	159	349	0.393	-32.2	-2.52	268	197	368	0.363	-14.4	-1.13	115	145

0.5	-	7.6	D	579	0.423	-14.8	-1.16	289	248	327	0.317	-21.9	-1.72	136	127	327	0.34	-31.9	-2.50	146	175	388	0.38	-7.8	-0.61	163	155
0.5	-	7.6	D	720	0.432	-13.5	-1.06	169	168	392	0.373	-23.2	-1.82	179	198	292	0.38	-33.2	-2.60	269	208	493	0.366	-10.3	-0.81	265	248
0.5	-	7.6	D	651	0.453	-14.4	-1.13	159	153	435	0.33	-12.0	-0.94	153	180	330	0.444	-21.5	-1.69	128	159	321	0.437	-24.3	-1.90	134	216
0.5	-	7.6	D	532	0.399	-14.2	-1.11	141	161	410	0.334	-13.1	-1.03	198	214	307	0.307	-18.7	-1.46	121	139	407	0.463	-28.2	-2.21	195	210
0.5	-	8.6	D	nu	>0.5	nu	nu	51	168	490	0.35	-25.7	-2.01	193	198	456	0.45	-20.0	-1.57	122	225	498	0.39	-20.4	-1.60	94	136
0.5	-	8.6	D	nu	>0.5	nu	nu	51	179	421	0.451	-23.0	-1.80	123	212	481	0.361	-25.0	-1.96	115	136	387	0.314	-27.3	-2.14	82	145
0.5	-	8.6	D	nu	>0.5	nu	nu	92	196	415	0.419	-19.9	-1.56	167	160	463	0.491	-24.9	-1.95	140	153	472	0.452	-30.5	-2.39	125	163
0.5	-	8.6	D	nu	>0.5	nu	nu	155	165	331	0.355	-11.6	-0.91	112	159	486	0.444	-17.0	-1.33	126	150	482	0.436	-30.5	-2.39	114	148
0.5	-	8.6	D	nu	>0.5	nu	nu	159	159	283	0.375	-17.1	-1.34	209	161	412	0.333	-19.2	-1.51	114	144	461	0.467	-28.8	-2.26	131	165
5	1	6	D	534	0.44	-23.5	-1.84	225	200	441	0.399	-23.7	-1.86	219	232	348	0.396	-25.2	-1.98	221	210	277	0.467	-24.9	-1.95	139	171
5	2	6	D	519	0.428	-24.1	-1.89	213	204	413	0.332	-23.7	-1.86	179	155	319	0.383	-25.5	-2.00	216	205	302	0.418	-26.6	-2.08	137	188
5	3	6	D	504	0.469	-22.9	-1.79	234	239	502	0.497	-20.0	-1.57	269	249	429	0.458	-22.8	-1.79	194	239	410	0.436	-24.8	-1.94	178	217
5	4	6	D	526	0.467	-25.7	-2.01	253	242	439	0.481	-20.8	-1.63	198	259	464	0.457	-22.9	-1.79	239	291	455	0.469	-24.6	-1.93	149	185
5	5	6	D	592	0.49	-26.0	-2.04	219	211	404	0.496	-21.7	-1.70	124	240	405	0.494	-22.8	-1.79	208	213	474	0.366	-26.7	-2.09	165	212
5	1	7.6	D	475	0.462	-22.8	-1.79	224	224	300	0.343	-21.2	-1.66	293	418	363	0.35	-24.8	-1.94	185	245	255	0.471	-25.6	-2.01	156	169
5	2	7.6	D	414	0.463	-26.5	-2.08	256	233	413	0.467	-22.2	-1.74	255	366	330	0.458	-27.2	-2.13	219	236	261	0.454	-26.6	-2.08	163	190
5	3	7.6	D	441	0.404	-25.7	-2.01	219	222	447	0.344	-22.8	-1.79	195	177	371	0.409	-25.2	-1.98	166	235	256	0.378	-26.9	-2.11	173	212
5	4	7.6	D	453	0.409	-25.1	-1.97	179	225	457	0.485	-20.5	-1.61	166	180	359	0.41	-24.3	-1.90	159	149	397	0.436	-25.0	-1.96	159	205
5	5	7.6	D	466	0.453	-17.6	-1.38	283	253	438	0.321	-20.5	-1.61	169	165	396	0.419	-26.1	-2.05	148	150	393	0.474	-22.2	-1.74	180	240
5	1	8.6	D	420	0.414	-28.0	-2.19	217	225	368	0.48	-25.9	-2.03	157	191	414	0.427	-25.2	-1.98	145	175	343	0.39	-27.2	-2.13	166	204
5	2	8.6	D	488	0.466	-30.0	-2.35	165	240	339	0.362	-27.1	-2.12	204	219	425	0.41	-27.8	-2.18	171	177	300	0.453	-28.9	-2.27	161	219
5	3	8.6	D	425	0.429	-29.3	-2.30	246	212	365	0.401	-18.3	-1.43	219	202	421	0.499	-23.2	-1.82	135	171	289	0.494	-29.1	-2.28	170	229

5	4	8.6	D	470	0.49	-27.1	-2.12	239	255	360	0.386	-18.6	-1.46	197	285	440	0.499	-25.6	-2.01	217	248	426	0.42	-25.6	-2.01	199	219
5	5	8.6	D	452	0.414	-26.3	-2.06	244	248	368	0.465	-19.5	-1.53	214	226	371	0.428	-25.4	-1.99	176	255	442	0.356	-25.4	-1.99	228	243
5	-	6	D	523	0.421	-24.6	-1.93	167	174	473	0.343	-22.1	-1.73	209	184	307	0.433	-31.9	-2.50	219	236	471	0.341	-27.0	-2.12	129	184
5	-	6	D	512	0.355	-23.5	-1.84	150	192	415	0.49	-23.3	-1.83	275	257	297	0.448	-31.2	-2.45	165	221	297	0.36	-23.7	-1.86	138	194
5	-	6	D	542	0.402	-18.4	-1.45	236	212	488	0.457	-26.1	-2.05	239	215	271	0.399	-34.9	-2.74	154	174	364	0.46	-30.2	-2.37	113	186
5	-	6	D	559	0.468	-17.9	-1.41	158	155	423	0.387	-25.6	-2.01	196	246	451	0.449	-26.0	-2.04	151	184	422	0.408	-27.7	-2.17	211	175
5	-	6	D	476	0.399	-19.5	-1.53	109	148	483	0.422	-24.0	-1.88	142	161	460	0.397	-25.6	-2.01	159	182	446	0.405	-32.8	-2.57	143	147
5	-	7.6	D	nu	>0.5	nu	nu	203	174	457	0.452	-26.9	-2.11	296	231	479	0.231	-31.8	-2.49	212	235	451	0.434	-31.8	-2.49	124	154
5	-	7.6	D	nu	>0.5	nu	nu	198	191	479	0.462	-19.7	-1.54	326	331	487	0.279	-29.3	-2.30	158	222	398	0.475	-32.5	-2.55	147	158
5	-	7.6	D	nu	>0.5	nu	nu	158	167	382	0.454	-17.7	-1.39	147	180	339	0.504	-26.2	-2.05	141	218	492	0.315	-33.9	-2.66	126	136
5	-	7.6	D	nu	>0.5	nu	nu	170	281	460	0.466	-13.4	-1.05	159	151	364	0.43	-22.2	-1.74	213	234	457	0.479	-33.8	-2.65	126	163
5	-	7.6	D	nu	>0.5	nu	nu	238	287	450	0.393	-17.1	-1.34	159	155	282	0.397	-22.8	-1.79	217	236	408	0.37	-34.7	-2.72	136	154
5	-	8.6	D	nu	>0.5	nu	nu	162	194	473	0.476	-26.7	-2.09	121	151	495	0.338	-20.5	-1.61	142	163	490	0.364	-31.1	-2.44	110	130
5	-	8.6	D	nu	>0.5	nu	nu	208	284	538	0.451	-20.0	-1.57	147	151	487	0.289	-21.3	-1.67	170	161	472	0.412	-32.8	-2.57	109	123
5	-	8.6	D	nu	>0.5	nu	nu	208	215	338	0.429	-26.1	-2.05	164	165	491	0.297	-22.7	-1.78	157	166	464	0.327	-35.5	-2.78	121	156
5	-	8.6	D	nu	>0.5	nu	nu	183	240	389	0.457	-25.8	-2.02	269	245	383	0.45	-25.1	-1.97	247	342	417	0.351	-33.9	-2.66	129	155
5	-	8.6	D	nu	>0.5	nu	nu	178	242	354	0.484	-19.8	-1.55	220	241	321	0.428	-24.6	-1.93	123	204	330	0.384	-23.6	-1.85	118	148
0.5	1	6	L	317	0.354	-19.4	-1.52	190	230	467	0.454	-19.3	-1.51	170	223	319	0.37	-15.3	-1.20	217	229	402	0.44	-25.8	-2.02	100	170
0.5	2	6	L	305	0.382	-20.6	-1.61	192	215	348	0.37	-17.0	-1.33	162	215	361	0.485	-17.7	-1.39	226	228	450	0.489	-23.2	-1.82	121	163
0.5	3	6	L	233	0.364	-22.8	-1.79	159	221	256	0.343	-17.0	-1.33	199	195	436	0.412	-17.1	-1.34	218	230	440	0.399	-27.6	-2.16	121	126
0.5	4	6	L	266	0.431	-21.4	-1.68	219	235	271	0.432	-22.5	-1.77	153	212	480	0.453	-21.1	-1.65	167	191	349	0.426	-22.5	-1.76	122	124
0.5	5	6	L	341	0.451	-20.1	-1.57	176	220	261	0.45	-23.3	-1.82	144	229	383	0.405	-21.9	-1.72	229	243	330	0.436	-24.8	-1.94	104	111

0.5	1	7.6	L	290	0.315	-22.5	-1.77	209	220	285	0.43	-24.6	-1.93	157	213	452	0.429	-21.7	-1.70	272	253	346	0.352	-19.9	-1.56	139	154
0.5	2	7.6	L	301	0.428	-23.7	-1.86	209	245	278	0.445	-26.2	-2.05	215	200	464	0.455	-23.2	-1.82	224	244	360	0.319	-19.0	-1.49	95	150
0.5	3	7.6	L	288	0.345	-15.7	-1.23	180	222	267	0.397	-17.6	-1.38	158	200	516	0.488	-23.1	-1.81	202	295	335	0.404	-19.1	-1.50	117	162
0.5	4	7.6	L	275	0.365	-14.5	-1.13	175	254	280	0.454	-24.5	-1.92	229	254	366	0.403	-22.7	-1.78	189	259	335	0.46	-19.3	-1.51	114	133
0.5	5	7.6	L	306	0.447	-17.5	-1.37	209	226	278	0.326	-15.1	-1.18	173	229	351	0.465	-23.0	-1.80	203	260	338	0.401	-21.2	-1.66	88	126
0.5	1	8.6	L	396	0.486	-15.8	-1.24	223	233	425	0.401	-17.5	-1.37	227	209	298	0.385	-18.4	-1.44	175	228	335	0.427	-19.1	-1.50	121	172
0.5	2	8.6	L	336	0.527	-14.1	-1.11	209	211	374	0.346	-16.6	-1.30	189	229	297	0.484	-18.8	-1.47	228	322	333	0.429	-22.6	-1.77	138	170
0.5	3	8.6	L	385	0.472	-22.4	-1.76	179	215	362	0.374	-21.6	-1.69	197	216	253	0.424	-20.1	-1.58	195	236	406	0.376	-23.3	-1.83	131	176
0.5	4	8.6	L	203	0.435	-22.3	-1.74	183	220	494	0.448	-11.2	-0.88	219	209	413	0.449	-22.2	-1.74	174	200	426	0.377	-23.0	-1.80	100	136
0.5	5	8.6	L	204	0.422	-20.8	-1.63	197	223	381	0.417	-20.6	-1.62	224	237	403	0.437	-22.5	-1.76	158	205	426	0.372	-19.5	-1.53	102	123
0.5	-	6	L	544	0.483	-22.8	-1.79	227	222	373	0.378	-20.0	-1.57	155	212	424	0.382	-17.4	-1.36	138	151	324	0.39	-21.0	-1.65	149	159
0.5	-	6	L	426	0.381	-20.1	-1.58	60	136	448	0.419	-19.7	-1.55	263	236	409	0.367	-18.6	-1.45	192	192	423	0.377	-22.4	-1.76	181	203
0.5	-	6	L	427	0.383	-20.5	-1.61	148	216	504	0.158	-20.2	-1.58	111	184	449	0.339	-24.8	-1.94	298	228	407	0.408	-21.6	-1.69	186	176
0.5	-	6	L	418	0.387	-16.6	-1.30	117	192	469	0.228	-18.9	-1.48	174	227	491	0.456	-24.8	-1.94	192	337	432	0.469	-18.6	-1.46	136	168
0.5	-	6	L	522	0.452	-20.3	-1.59	175	266	352	0.308	-18.9	-1.48	93	174	343	0.485	-24.4	-1.91	92	166	322	0.479	-19.2	-1.50	138	150
0.5	-	7.6	L	nu	>0.5	nu	nu	89	143	481	0.118	-181.3	-14.21	269	195	346	0.42	-27.3	-2.14	106	125	370	0.419	-16.8	-1.32	154	167
0.5	-	7.6	L	nu	>0.5	nu	nu	87	143	427	0.247	-17.6	-1.38	153	177	347	0.338	-27.1	-2.12	107	124	304	0.429	-19.5	-1.53	107	184
0.5	-	7.6	L	nu	>0.5	nu	nu	118	168	443	0.463	-23.9	-1.87	99	152	323	0.321	-24.5	-1.92	229	209	304	0.417	-17.7	-1.39	119	212
0.5	-	7.6	L	nu	>0.5	nu	nu	132	130	450	0.383	-23.0	-1.80	143	172	231	0.429	-25.8	-2.02	257	292	310	0.419	-25.2	-1.98	188	228
0.5	-	7.6	L	nu	>0.5	nu	nu	119	181	366	0.283	-21.1	-1.65	137	206	240	0.356	-24.5	-1.92	183	196	358	0.376	-27.9	-2.19	119	150
0.5	-	8.6	L	nu	>0.5	nu	nu	103	118	412	0.423	-21.6	-1.69	98	113	313	0.325	-27.6	-2.16	110	151	355	0.392	-27.0	-2.12	125	153
0.5	-	8.6	L	nu	>0.5	nu	nu	113	137	409	0.385	-22.9	-1.79	86	130	413	0.482	-24.9	-1.95	98	151	355	0.416	-23.6	-1.85	129	137

0.5	-	8.6	L	nu	>0.5	nu	nu	98	142	254	0.416	-22.6	-1.77	96	127	360	0.399	-30.3	-2.37	127	149	319	0.43	-24.6	-1.93	171	250
0.5	-	8.6	L	nu	>0.5	nu	nu	108	109	248	0.355	-17.2	-1.35	67	152	355	0.309	-30.8	-2.41	124	135	329	0.444	-29.3	-2.30	144	238
0.5	-	8.6	L	nu	>0.5	nu	nu	132	197	248	0.369	-25.5	-2.00	68	209	337	0.33	-30.9	-2.42	163	142	320	0.482	-22.9	-1.79	259	234
5	1	6	L	307	0.496	-24.9	-1.95	193	213	358	0.326	-26.2	-2.05	206	215	470	0.441	-23.9	-1.87	269	280	457	0.413	-27.0	-2.12	187	192
5	2	6	L	248	0.435	-24.7	-1.94	197	198	347	0.304	-27.4	-2.15	262	238	460	0.409	-25.1	-1.97	211	224	448	0.468	-27.5	-2.16	191	188
5	3	6	L	269	0.368	-19.5	-1.53	196	194	325	0.39	-22.9	-1.79	244	264	425	0.498	-27.2	-2.13	217	239	396	0.446	-26.2	-2.05	132	188
5	4	6	L	220	0.373	-22.4	-1.76	186	214	316	0.326	-23.8	-1.87	162	245	391	0.603	-26.6	-2.08	182	185	353	0.397	-26.6	-2.08	185	190
5	5	6	L	254	0.405	-20.4	-1.60	167	188	295	0.339	-24.0	-1.88	156	170	469	0.443	-24.3	-1.90	159	173	328	0.408	-32.1	-2.52	166	208
5	1	7.6	L	379	0.458	-22.0	-1.72	182	146	358	0.37	-30.2	-2.37	202	229	434	0.423	-28.2	-2.21	167	220	474	0.381	-28.6	-2.24	141	192
5	2	7.6	L	378	0.437	-18.4	-1.44	237	275	347	0.326	-31.0	-2.43	180	253	417	0.436	-30.7	-2.41	161	176	478	0.415	-30.6	-2.40	175	189
5	3	7.6	L	282	0.331	-14.6	-1.14	227	231	389	0.333	-28.6	-2.24	212	244	443	0.465	-28.4	-2.23	186	211	417	0.436	-29.4	-2.30	169	196
5	4	7.6	L	322	0.438	-19.2	-1.50	252	295	396	0.304	-24.9	-1.95	230	232	424	0.451	-28.2	-2.21	193	197	341	0.388	-33.2	-2.60	124	159
5	5	7.6	L	381	0.417	-24.5	-1.92	225	260	389	0.459	-23.5	-1.84	226	239	406	0.404	-29.9	-2.34	206	227	335	0.413	-31.5	-2.47	139	157
5	1	8.6	L	nu	>0.5	nu	nu	178	248	442	0.381	-29.5	-2.31	171	172	430	0.48	-32.1	-2.52	236	232	453	0.451	-28.8	-2.26	172	198
5	2	8.6	L	nu	>0.5	nu	nu	195	272	437	0.331	-30.1	-2.36	197	199	454	0.392	-21.3	-1.67	231	230	448	0.394	-30.5	-2.39	174	194
5	3	8.6	L	nu	>0.5	nu	nu	252	255	412	0.385	-29.5	-2.31	217	199	471	0.39	-17.7	-1.39	159	205	403	0.396	-27.5	-2.16	157	190
5	4	8.6	L	nu	>0.5	nu	nu	198	252	460	0.304	-28.5	-2.23	181	230	462	0.427	-24.1	-1.89	98	164	417	0.409	-26.8	-2.10	234	205
5	5	8.6	L	nu	>0.5	nu	nu	193	232	435	0.451	-27.9	-2.19	246	250	406	0.487	-26.5	-2.08	149	148	411	0.367	-28.2	-2.21	170	218
5	-	6	L	605	0.411	-21.1	-1.65	115	132	337	0.373	-31.6	-2.48	265	216	286	0.41	-32.3	-2.53	183	223	368	0.413	-32.4	-2.54	109	169
5	-	6	L	569	0.468	-16.9	-1.33	226	248	353	0.321	-27.5	-2.16	254	222	263	0.42	-32.1	-2.52	181	277	317	0.398	-32.0	-2.51	130	155
5	-	6	L	560	0.492	-17.1	-1.34	159	199	398	0.337	-23.8	-1.87	203	237	210	0.334	-31.4	-2.46	217	250	373	0.337	-29.1	-2.28	142	161
5	-	6	L	440	0.445	-11.1	-0.87	103	138	401	0.405	-26.2	-2.05	199	166	208	0.246	-31.1	-2.44	169	191	277	0.357	-30.7	-2.41	172	164

5	-	6	L	543	0.461	-17.8	-1.40	90	193	309	0.397	-27.9	-2.19	188	187	211	0.311	-33.6	-2.63	207	202	254	0.384	-33.8	-2.65	117	169
5	-	7.6	L	378	0.497	-23.9	-1.87	203	180	389	0.355	-33.3	-2.61	113	149	298	0.362	-35.2	-2.76	162	173	241	0.442	-33.3	-2.61	133	150
5	-	7.6	L	313	0.493	-23.2	-1.82	186	205	421	0.386	-34.9	-2.74	111	148	268	0.404	-36.0	-2.82	183	155	229	0.364	-34.9	-2.74	128	149
5	-	7.6	L	537	0.464	-23.3	-1.83	169	187	364	0.397	-35.0	-2.74	152	147	238	0.459	-36.1	-2.83	294	418	234	0.351	-33.5	-2.63	145	144
5	-	7.6	L	855	0.481	-27.6	-2.16	215	210	417	0.389	-23.2	-1.82	229	228	289	0.461	-31.1	-2.44	326	444	244	0.375	-33.2	-2.60	127	150
5	-	7.6	L	890	0.419	-24.7	-1.94	170	236	450	0.471	-19.4	-1.52	224	242	306	0.472	-31.0	-2.43	162	162	237	0.404	-31.2	-2.45	127	144
5	-	8.6	L	410	0.412	-28.7	-2.25	111	150	335	0.448	-32.3	-2.53	216	218	239	0.275	-31.6	-2.48	209	206	212	0.419	-34.3	-2.69	116	137
5	-	8.6	L	409	0.441	-26.6	-2.08	219	194	331	0.411	-33.6	-2.63	323	275	243	0.311	-33.5	-2.63	149	244	217	0.35	-36.2	-2.84	123	148
5	-	8.6	L	342	0.419	-17.3	-1.36	162	178	397	0.394	-16.5	-1.29	253	234	330	0.359	-29.5	-2.31	233	232	222	0.437	-36.6	-2.87	145	166
5	-	8.6	L	556	0.413	-16.6	-1.30	179	195	395	0.458	-22.0	-1.72	179	152	344	0.345	-32.2	-2.52	177	199	388	0.479	-29.8	-2.34	121	153
5	-	8.6	L	410	0.391	-19.9	-1.56	193	206	388	0.32	-19.2	-1.50	172	141	374	0.342	-35.1	-2.75	289	269	293	0.465	-25.1	-1.97	113	155
