# Supplementary material for: Extracellular Polymeric Substances (EPS) of Freshwater Biofilms Stabilize and Modify CeO2 and Ag Nanoparticles
Source: PLoS One. 2014 Oct 21;9(10):e110709. doi: 10.1371/journal.pone.0110709 (PMC4204993; doi:10.1371/journal.pone.0110709)
Supplement: Table S10 — Standard deviations of Z-averages (DLS), polydispersity (PDI), mode and mean diameters (NTA), zetapotential, and EPM of CeO2 NP dispersions dependent on pH, light/dark, EPS content, and time. (PDF) [file pone.0110709.s018.pdf]

				3 h						24 h						168 h						336 h					
mg/L	EPS	pH	L/D	z-average	PDI	ZP	EPM	Mode	Mean	z-average	PDI	ZP	EPM	Mode	Mean	z-average	PDI	ZP	EPM	Mode	Mean	z-average	PDI	ZP	EPM	Mode	Mean
0.5	1	6	D	54.8	0.09	3.4	0.3	39.8	44.4	75.9	0.09	3.9	0.3	39.6	44.0	65.5	0.09	4.0	0.3	40.6	44.0	65.4	0.08	3.6	0.3	37.2	31.6
0.5	2	6	D	58.1	0.08	3.4	0.3	37.8	51.2	79.0	0.08	4.1	0.3	47.8	46.6	55.9	0.09	3.9	0.3	53.4	48.8	79.6	0.07	3.5	0.3	35.8	34.0
0.5	3	6	D	55.5	0.08	2.4	0.2	41.8	43.0	93.5	0.08	2.3	0.2	49.6	51.0	79.0	0.08	4.1	0.3	37.8	41.2	77.1	0.09	3.0	0.2	32.8	34.8
0.5	4	6	D	53.6	0.08	2.8	0.2	40.6	47.4	85.1	0.08	2.6	0.2	42.2	41.4	66.6	0.09	3.5	0.3	44.8	44.2	67.8	0.09	3.9	0.3	20.2	26.2
0.5	5	6	D	57.5	0.07	2.6	0.2	37.0	43.8	86.2	0.08	3.0	0.2	37.6	48.2	69.8	0.07	3.8	0.3	57.2	44.2	53.3	0.08	3.8	0.3	23.4	28.0
0.5	1	7.6	D	60.4	0.08	3.4	0.3	40.6	44.0	78.1	0.07	3.7	0.3	35.8	43.4	68.1	0.09	2.9	0.2	43.8	49.6	51.7	0.07	3.2	0.3	24.0	28.4
0.5	2	7.6	D	60.1	0.07	4.0	0.3	49.8	49.2	70.4	0.07	3.9	0.3	38.0	43.2	64.5	0.07	3.1	0.2	39.6	59.0	48.0	0.07	3.4	0.3	22.0	25.2
0.5	3	7.6	D	62.8	0.07	3.7	0.3	45.0	44.4	68.5	0.06	3.4	0.3	47.6	54.8	69.4	0.09	4.0	0.3	35.8	54.0	48.9	0.07	3.3	0.3	20.6	25.2
0.5	4	7.6	D	61.4	0.07	2.7	0.2	37.4	44.6	71.5	0.09	3.0	0.2	38.8	49.8	81.5	0.08	4.3	0.3	39.8	54.8	52.4	0.08	4.3	0.3	24.4	30.8
0.5	5	7.6	D	59.9	0.07	3.3	0.3	43.8	50.2	76.9	0.09	3.2	0.3	37.8	43.0	91.1	0.09	4.4	0.3	51.4	52.4	68.0	0.10	3.3	0.3	24.8	31.2
0.5	1	8.6	D	83.6	0.09	3.2	0.2	65.8	60.2	87.1	0.09	3.8	0.3	38.4	39.2	87.1	0.09	3.8	0.3	37.6	39.4	58.7	0.08	3.5	0.3	25.0	30.2
0.5	2	8.6	D	60.8	0.06	4.4	0.3	52.8	50.6	87.7	0.09	4.5	0.3	37.2	46.0	85.8	0.06	4.3	0.3	24.4	35.6	78.2	0.07	3.5	0.3	33.2	29.4
0.5	3	8.6	D	69.9	0.07	3.6	0.3	33.0	40.0	63.0	0.07	2.4	0.2	34.8	54.4	67.7	0.09	4.5	0.3	27.8	55.6	42.5	0.10	3.3	0.3	35.2	37.2
0.5	4	8.6	D	68.7	0.08	3.3	0.3	41.8	45.0	76.3	0.07	3.1	0.2	43.6	48.8	60.5	0.09	4.4	0.3	35.6	58.0	41.9	0.08	3.5	0.3	29.0	43.0
0.5	5	8.6	D	77.5	0.09	2.8	0.2	33.8	46.4	75.6	0.07	3.0	0.2	56.6	50.6	79.1	0.08	4.6	0.4	67.8	54.2	48.7	0.10	4.0	0.3	29.2	36.8
0.5	-	6	D	96.1	0.07	3.4	0.3	27.4	39.4	95.5	0.09	3.9	0.3	25.8	44.6	75.5	0.08	3.7	0.3	52.6	38.6	78.8	0.07	2.9	0.2	29.4	30.0
0.5	-	6	D	114.7	0.10	2.6	0.2	46.6	46.4	89.1	0.08	4.9	0.4	26.2	40.8	69.1	0.08	2.9	0.2	30.8	42.6	60.1	0.06	3.2	0.2	44.6	44.6
0.5	-	6	D	123.5	0.09	3.3	0.3	53.4	51.2	86.2	0.09	5.2	0.4	38.8	41.2	66.2	0.07	4.0	0.3	30.8	34.0	52.3	0.08	3.1	0.2	24.4	43.6
0.5	-	6	D	128.4	0.10	3.1	0.2	23.8	28.4	65.5	0.07	2.6	0.2	42.4	43.8	60.6	0.10	2.6	0.2	27.8	30.6	74.4	0.07	3.8	0.3	28.8	36.0
0.5	-	6	D	99.2	0.10	2.8	0.2	27.8	39.8	64.4	0.10	4.0	0.3	43.8	46.4	79.7	0.10	4.5	0.4	49.8	52.8	70.2	0.07	3.3	0.3	23.4	31.2
0.5	-	7.6	D	150.4	0.08	2.6	0.2	12.2	27.6	63.8	0.07	4.4	0.3	25.4	31.8	69.8	0.08	6.4	0.5	53.6	39.4	73.5	0.07	2.9	0.2	23.0	29.0

0.5	-	7.6	D	115.7	0.08	3.0	0.2	57.8	49.6	65.4	0.06	4.4	0.3	27.2	25.4	65.4	0.07	6.4	0.5	29.2	35.0	77.7	0.08	1.6	0.1	32.6	31.0
0.5	-	7.6	D	143.9	0.09	2.7	0.2	33.8	33.6	78.4	0.07	4.6	0.4	35.8	39.6	58.4	0.08	6.6	0.5	53.8	41.6	98.6	0.07	2.1	0.2	53.0	49.6
0.5	-	7.6	D	130.2	0.09	2.9	0.2	31.8	30.6	87.0	0.07	2.4	0.2	30.6	36.0	66.1	0.09	4.3	0.3	25.6	31.8	64.2	0.09	4.9	0.4	26.8	43.2
0.5	-	7.6	D	106.3	0.08	2.8	0.2	28.2	32.2	82.0	0.07	2.6	0.2	39.6	42.8	61.5	0.06	3.7	0.3	24.2	27.8	81.3	0.09	5.6	0.4	39.0	42.0
0.5	-	8.6	D					10.2	33.6	98.0	0.07	5.1	0.4	38.6	39.6	91.2	0.09	4.0	0.3	24.4	45.0	99.7	0.08	4.1	0.3	18.8	27.2
0.5	-	8.6	D					10.2	35.8	84.1	0.09	4.6	0.4	24.6	42.4	96.1	0.07	5.0	0.4	23.0	27.2	77.4	0.06	5.5	0.4	16.4	29.0
0.5	-	8.6	D					18.4	39.2	83.0	0.08	4.0	0.3	33.4	32.0	92.6	0.10	5.0	0.4	28.0	30.6	94.4	0.09	6.1	0.5	25.0	32.6
0.5	-	8.6	D					31.0	33.0	66.2	0.07	2.3	0.2	22.4	31.8	97.2	0.09	3.4	0.3	25.2	30.0	96.5	0.09	6.1	0.5	22.8	29.6
0.5	-	8.6	D					31.8	31.8	56.6	0.08	3.4	0.3	41.8	32.2	82.4	0.07	3.8	0.3	22.8	28.8	92.1	0.09	5.8	0.5	26.2	33.0
5	1	6	D	106.9	0.09	4.7	0.4	45.0	40.0	88.2	0.08	4.7	0.4	43.8	46.4	69.5	0.08	5.0	0.4	44.2	42.0	55.4	0.09	5.0	0.4	27.8	34.2
5	2	6	D	103.8	0.09	4.8	0.4	42.6	40.8	82.6	0.07	4.7	0.4	35.8	31.0	63.7	0.08	5.1	0.4	43.2	41.0	60.4	0.08	5.3	0.4	27.4	37.6
5	3	6	D	100.8	0.09	4.6	0.4	46.8	47.8	100.3	0.10	4.0	0.3	53.8	49.8	85.8	0.09	4.6	0.4	38.8	47.8	82.1	0.09	5.0	0.4	35.6	43.4
5	4	6	D	105.3	0.09	5.1	0.4	50.6	48.4	87.7	0.10	4.2	0.3	39.6	51.8	92.7	0.09	4.6	0.4	47.8	58.2	91.0	0.09	4.9	0.4	29.8	37.0
5	5	6	D	118.5	0.10	5.2	0.4	43.8	42.2	80.7	0.10	4.3	0.3	24.8	48.0	80.9	0.10	4.6	0.4	41.6	42.5	94.8	0.07	5.3	0.4	33.0	42.5
5	1	7.6	D	95.1	0.09	4.6	0.4	44.8	44.8	60.0	0.07	4.2	0.3	58.6	83.6	72.7	0.07	5.0	0.4	37.0	49.0	51.0	0.09	5.1	0.4	31.2	33.9
5	2	7.6	D	82.8	0.09	5.3	0.4	51.2	46.6	82.6	0.09	4.4	0.3	51.0	73.2	65.9	0.09	5.4	0.4	43.8	47.2	52.2	0.09	5.3	0.4	32.6	38.0
5	3	7.6	D	88.3	0.08	5.1	0.4	43.8	44.4	89.5	0.07	4.6	0.4	39.0	35.4	74.2	0.08	5.0	0.4	33.2	47.0	51.2	0.08	5.4	0.4	34.6	42.4
5	4	7.6	D	90.6	0.08	5.0	0.4	35.8	45.0	91.4	0.10	4.1	0.3	33.2	36.0	71.8	0.08	4.9	0.4	31.8	29.8	79.4	0.09	5.0	0.4	31.8	41.0
5	5	7.6	D	93.1	0.09	3.5	0.3	56.6	50.6	87.6	0.06	4.1	0.3	33.8	33.0	79.2	0.08	5.2	0.4	29.6	30.0	78.6	0.09	4.4	0.3	36.0	48.0
5	1	8.6	D	84.0	0.08	5.6	0.4	43.4	45.0	73.5	0.10	5.2	0.4	31.4	38.2	82.8	0.09	5.0	0.4	29.0	35.0	68.5	0.08	5.4	0.4	33.2	40.8
5	2	8.6	D	97.6	0.09	6.0	0.5	33.0	48.0	67.9	0.07	5.4	0.4	40.8	43.8	85.1	0.08	5.6	0.4	34.2	35.4	60.0	0.09	5.8	0.5	32.2	43.8
5	3	8.6	D	85.0	0.09	5.9	0.5	49.2	42.4	72.9	0.08	3.7	0.3	43.8	40.4	84.1	0.10	4.6	0.4	27.0	34.2	57.7	0.10	5.8	0.5	34.0	45.8

5	4	8.6	D	94.1	0.10	5.4	0.4	47.8	51.0	71.9	0.08	3.7	0.3	39.4	57.0	88.0	0.10	5.1	0.4	43.4	49.6	85.2	0.08	5.1	0.4	39.8	43.8
5	5	8.6	D	90.4	0.08	5.3	0.4	48.8	49.6	73.7	0.09	3.9	0.3	42.8	45.2	74.2	0.09	5.1	0.4	35.2	51.0	88.3	0.07	5.1	0.4	45.6	48.7
5	-	6	D	104.5	0.08	4.9	0.4	33.4	34.8	94.7	0.07	4.4	0.3	41.8	36.8	61.5	0.09	6.4	0.5	43.8	47.2	94.2	0.07	5.4	0.4	25.8	36.7
5	-	6	D	102.3	0.07	4.7	0.4	30.0	38.4	82.9	0.10	4.7	0.4	55.0	51.4	59.4	0.09	6.2	0.5	33.0	44.3	59.4	0.07	4.7	0.4	27.6	38.8
5	-	6	D	108.4	0.08	3.7	0.3	47.2	42.4	97.7	0.09	5.2	0.4	47.8	43.0	54.3	0.08	7.0	0.5	30.8	34.9	72.9	0.09	6.0	0.5	22.6	37.3
5	-	6	D	111.8	0.09	3.6	0.3	31.6	31.0	84.6	0.08	5.1	0.4	39.2	49.2	90.1	0.09	5.2	0.4	30.2	36.8	84.4	0.08	5.5	0.4	42.2	35.1
5	-	6	D	95.3	0.08	3.9	0.3	21.8	29.6	96.7	0.08	4.8	0.4	28.4	32.3	91.9	0.08	5.1	0.4	31.8	36.4	89.2	0.08	6.6	0.5	28.6	29.3
5	-	7.6	D					40.6	34.8	91.4	0.09	5.4	0.4	59.2	46.2	95.7	0.05	6.4	0.5	42.4	47.0	90.2	0.09	6.4	0.5	24.8	30.7
5	-	7.6	D					39.6	38.2	95.8	0.09	3.9	0.3	65.2	66.2	97.4	0.06	5.9	0.5	31.6	44.4	79.6	0.10	6.5	0.5	29.4	31.6
5	-	7.6	D					31.6	33.4	76.3	0.09	3.5	0.3	29.4	36.0	67.9	0.10	5.2	0.4	28.2	43.6	98.4	0.06	6.8	0.5	25.2	27.2
5	-	7.6	D					34.0	56.1	92.0	0.09	2.7	0.2	31.8	30.2	72.8	0.09	4.4	0.3	42.6	46.8	91.4	0.10	6.8	0.5	25.2	32.6
5	-	7.6	D					47.6	57.3	90.0	0.08	3.4	0.3	31.8	31.0	56.4	0.08	4.6	0.4	43.4	47.2	81.6	0.07	6.9	0.5	27.2	30.8
5	-	8.6	D					32.4	38.8	94.7	0.10	5.3	0.4	24.2	30.2	99.0	0.07	4.1	0.3	28.4	32.6	97.9	0.07	6.2	0.5	22.0	26.0
5	-	8.6	D					41.6	56.8	107.5	0.09	4.0	0.3	29.4	30.2	97.4	0.06	4.3	0.3	34.0	32.2	94.4	0.08	6.6	0.5	21.8	24.7
5	-	8.6	D					41.6	42.9	67.5	0.09	5.2	0.4	32.8	33.0	98.2	0.06	4.5	0.4	31.4	33.2	92.8	0.07	7.1	0.6	24.2	31.2
5	-	8.6	D					36.6	48.1	77.7	0.09	5.2	0.4	53.8	49.0	76.6	0.09	5.0	0.4	49.4	68.4	83.3	0.07	6.8	0.5	25.8	31.0
5	-	8.6	D					35.6	48.4	70.8	0.10	4.0	0.3	44.0	48.2	64.3	0.09	4.9	0.4	24.6	40.8	65.9	0.08	4.7	0.4	23.6	29.7
0.5	1	6	L	63.3	0.07	3.9	0.3	38.0	46.0	93.3	0.09	3.9	0.3	34.0	44.6	63.8	0.07	3.1	0.2	43.4	45.8	80.4	0.09	5.2	0.4	20.0	34.0
0.5	2	6	L	61.0	0.08	4.1	0.3	38.4	43.0	69.6	0.07	3.4	0.3	32.4	43.0	72.1	0.10	3.5	0.3	45.2	45.6	89.9	0.10	4.6	0.4	24.2	32.6
0.5	3	6	L	46.5	0.07	4.6	0.4	31.8	44.2	51.1	0.07	3.4	0.3	39.8	39.0	87.3	0.08	3.4	0.3	43.6	46.0	87.9	0.08	5.5	0.4	24.2	25.2
0.5	4	6	L	53.1	0.09	4.3	0.3	43.8	47.0	54.1	0.09	4.5	0.4	30.6	42.4	96.0	0.09	4.2	0.3	33.4	38.2	69.9	0.09	4.5	0.4	24.4	24.8
0.5	5	6	L	68.2	0.09	4.0	0.3	35.2	44.0	52.2	0.09	4.7	0.4	28.8	45.8	76.7	0.08	4.4	0.3	45.8	48.6	66.0	0.09	5.0	0.4	20.8	22.2

0.5	1	7.6	L	58.1	0.06	4.5	0.4	41.8	44.0	57.0	0.09	4.9	0.4	31.4	42.6	90.4	0.09	4.3	0.3	54.4	50.6	69.2	0.07	4.0	0.3	27.8	30.8
0.5	2	7.6	L	60.1	0.09	4.7	0.4	41.8	49.0	55.6	0.09	5.2	0.4	43.0	40.0	92.8	0.09	4.6	0.4	44.8	48.8	71.9	0.06	3.8	0.3	19.0	30.0
0.5	3	7.6	L	57.6	0.07	3.1	0.2	36.0	44.4	53.4	0.08	3.5	0.3	31.6	40.0	103.2	0.10	4.6	0.4	40.4	59.0	67.1	0.08	3.8	0.3	23.4	32.4
0.5	4	7.6	L	54.9	0.07	2.9	0.2	35.0	50.8	56.1	0.09	4.9	0.4	45.8	50.8	73.2	0.08	4.5	0.4	37.8	51.8	67.0	0.09	3.9	0.3	22.8	26.6
0.5	5	7.6	L	61.1	0.09	3.5	0.3	41.8	45.2	55.7	0.07	3.0	0.2	34.6	45.8	70.2	0.09	4.6	0.4	40.6	52.0	67.6	0.08	4.2	0.3	17.6	25.2
0.5	1	8.6	L	79.3	0.10	3.2	0.2	44.6	46.6	85.0	0.08	3.5	0.3	45.4	41.8	59.6	0.08	3.7	0.3	35.0	45.6	67.0	0.09	3.8	0.3	24.2	34.4
0.5	2	8.6	L	67.1	0.11	2.8	0.2	41.8	42.2	74.8	0.07	3.3	0.3	37.8	45.8	59.5	0.10	3.8	0.3	45.6	64.4	66.7	0.09	4.5	0.4	27.6	34.0
0.5	3	8.6	L	77.1	0.09	4.5	0.4	35.8	43.0	72.5	0.07	4.3	0.3	39.4	43.2	50.6	0.08	4.0	0.3	39.0	47.2	81.2	0.08	4.7	0.4	26.2	35.2
0.5	4	8.6	L	40.5	0.09	4.5	0.3	36.6	44.0	98.8	0.09	2.2	0.2	43.8	41.8	82.6	0.09	4.4	0.3	34.8	40.0	85.2	0.08	4.6	0.4	20.0	27.2
0.5	5	8.6	L	40.7	0.08	4.2	0.3	39.4	44.6	76.2	0.08	4.1	0.3	44.8	47.4	80.6	0.09	4.5	0.4	31.6	41.0	85.2	0.07	3.9	0.3	20.4	24.6
0.5	-	6	L	108.8	0.10	4.6	0.4	45.4	44.4	74.5	0.08	4.0	0.3	31.0	42.4	84.8	0.08	3.5	0.3	27.6	30.2	64.8	0.08	4.2	0.3	29.8	31.8
0.5	-	6	L	85.3	0.08	4.0	0.3	12.0	27.2	89.5	0.08	3.9	0.3	52.6	47.2	81.8	0.07	3.7	0.3	38.4	38.4	84.6	0.08	4.5	0.4	36.2	40.6
0.5	-	6	L	85.5	0.08	4.1	0.3	29.6	43.2	100.9	0.03	4.0	0.3	22.2	36.8	89.7	0.07	5.0	0.4	59.6	45.6	81.5	0.08	4.3	0.3	37.2	35.2
0.5	-	6	L	83.5	0.08	3.3	0.3	23.4	38.4	93.7	0.05	3.8	0.3	34.8	45.4	98.2	0.09	5.0	0.4	38.4	67.4	86.4	0.09	3.7	0.3	27.2	33.6
0.5	-	6	L	104.5	0.09	4.1	0.3	35.0	53.2	70.3	0.06	3.8	0.3	18.6	34.8	68.6	0.10	4.9	0.4	18.4	33.2	64.3	0.10	3.8	0.3	27.6	30.0
0.5	-	7.6	L					17.8	28.6	96.3	0.02	36.3	2.8	53.8	39.0	69.2	0.08	5.5	0.4	21.2	25.0	74.1	0.08	3.4	0.3	30.8	33.4
0.5	-	7.6	L					17.4	28.6	85.3	0.05	3.5	0.3	30.6	35.4	69.4	0.07	5.4	0.4	21.4	24.8	60.7	0.09	3.9	0.3	21.4	36.8
0.5	-	7.6	L					23.6	33.6	88.5	0.09	4.8	0.4	19.8	30.4	64.7	0.06	4.9	0.4	45.8	41.8	60.8	0.08	3.5	0.3	23.8	42.4
0.5	-	7.6	L					26.4	26.0	90.1	0.08	4.6	0.4	28.6	34.4	46.2	0.09	5.2	0.4	51.4	58.4	62.0	0.08	5.0	0.4	37.6	45.6
0.5	-	7.6	L					23.8	36.2	73.2	0.06	4.2	0.3	27.4	41.2	48.0	0.07	4.9	0.4	36.6	39.2	71.5	0.08	5.6	0.4	23.8	30.0
0.5	-	8.6	L					20.6	23.6	82.3	0.08	4.3	0.3	19.6	22.6	62.5	0.07	5.5	0.4	22.0	30.2	70.9	0.08	5.4	0.4	25.0	30.6
0.5	-	8.6	L					22.6	27.4	81.8	0.08	4.6	0.4	17.2	26.0	82.7	0.10	5.0	0.4	19.6	30.2	70.9	0.08	4.7	0.4	25.8	27.4

0.5	-	8.6	L					19.6	28.4	50.8	0.08	4.5	0.4	19.2	25.4	71.9	0.08	6.1	0.5	25.4	29.8	63.9	0.09	4.9	0.4	34.2	50.0
0.5	-	8.6	L					21.6	21.8	49.6	0.07	3.4	0.3	13.4	30.4	70.9	0.06	6.2	0.5	24.8	27.0	65.8	0.09	5.9	0.5	28.8	47.6
0.5	-	8.6	L					26.4	39.4	49.6	0.07	5.1	0.4	13.6	41.8	67.3	0.07	6.2	0.5	32.6	28.4	64.0	0.10	4.6	0.4	51.8	46.8
5	1	6	L	61.4	0.10	5.0	0.4	38.6	42.6	71.7	0.07	5.2	0.4	41.2	43.0	94.0	0.09	4.8	0.4	53.8	56.0	91.5	0.08	5.4	0.4	37.4	38.4
5	2	6	L	49.6	0.09	4.9	0.4	39.4	39.6	69.4	0.06	5.5	0.4	52.4	47.6	91.9	0.08	5.0	0.4	42.2	44.8	89.5	0.09	5.5	0.4	38.2	37.6
5	3	6	L	53.7	0.07	3.9	0.3	39.2	38.8	65.0	0.08	4.6	0.4	48.8	52.8	85.1	0.10	5.4	0.4	43.4	47.8	79.3	0.09	5.2	0.4	26.4	37.6
5	4	6	L	43.9	0.07	4.5	0.4	37.2	42.8	63.3	0.07	4.8	0.4	32.4	49.0	78.1	0.12	5.3	0.4	36.4	37.0	70.6	0.08	5.3	0.4	37.0	38.0
5	5	6	L	50.8	0.08	4.1	0.3	33.4	37.6	59.0	0.07	4.8	0.4	31.2	34.0	93.9	0.09	4.9	0.4	31.8	34.6	65.6	0.08	6.4	0.5	33.2	41.7
5	1	7.6	L	75.9	0.09	4.4	0.3	36.4	29.2	71.6	0.07	6.0	0.5	40.4	45.8	86.7	0.08	5.6	0.4	33.4	44.0	94.7	0.08	5.7	0.4	28.2	38.4
5	2	7.6	L	75.5	0.09	3.7	0.3	47.4	55.0	69.4	0.07	6.2	0.5	36.0	50.6	83.4	0.09	6.1	0.5	32.2	35.2	95.5	0.08	6.1	0.5	35.0	37.7
5	3	7.6	L	56.5	0.07	2.9	0.2	45.4	46.2	77.8	0.07	5.7	0.4	42.4	48.7	88.7	0.09	5.7	0.4	37.2	42.1	83.5	0.09	5.9	0.5	33.8	39.2
5	4	7.6	L	64.4	0.09	3.8	0.3	50.4	59.0	79.2	0.06	5.0	0.4	46.0	46.3	84.7	0.09	5.6	0.4	38.6	39.4	68.1	0.08	6.6	0.5	24.8	31.8
5	5	7.6	L	76.2	0.08	4.9	0.4	45.0	52.0	77.7	0.09	4.7	0.4	45.2	47.8	81.2	0.08	6.0	0.5	41.2	45.4	67.0	0.08	6.3	0.5	27.8	31.3
5	1	8.6	L					35.6	49.6	88.4	0.08	5.9	0.5	34.2	34.4	86.0	0.10	6.4	0.5	47.2	46.4	90.5	0.09	5.8	0.5	34.4	39.6
5	2	8.6	L					39.0	54.4	87.5	0.07	6.0	0.5	39.4	39.8	90.7	0.08	4.3	0.3	46.2	46.0	89.7	0.08	6.1	0.5	34.8	38.8
5	3	8.6	L					50.4	51.0	82.4	0.08	5.9	0.5	43.4	39.8	94.3	0.08	3.5	0.3	31.8	41.0	80.6	0.08	5.5	0.4	31.4	38.0
5	4	8.6	L					39.6	50.4	91.9	0.06	5.7	0.4	36.2	46.0	92.4	0.09	4.8	0.4	19.6	32.8	83.4	0.08	5.4	0.4	46.8	41.0
5	5	8.6	L					38.6	46.4	86.9	0.09	5.6	0.4	49.2	50.1	81.3	0.10	5.3	0.4	29.8	29.6	82.2	0.07	5.6	0.4	34.0	43.6
5	-	6	L	120.9	0.08	4.2	0.3	23.0	26.4	67.3	0.07	6.3	0.5	53.0	43.2	57.1	0.08	6.5	0.5	36.6	44.6	73.6	0.08	6.5	0.5	21.8	33.7
5	-	6	L	113.7	0.09	3.4	0.3	45.2	49.6	70.6	0.06	5.5	0.4	50.8	44.4	52.5	0.08	6.4	0.5	36.2	55.4	63.4	0.08	6.4	0.5	26.0	30.9
5	-	6	L	112.0	0.10	3.4	0.3	31.8	39.8	79.6	0.07	4.8	0.4	40.6	47.4	42.0	0.07	6.3	0.5	43.4	50.0	74.5	0.07	5.8	0.5	28.4	32.1
5	-	6	L	87.9	0.09	2.2	0.2	20.6	27.7	80.2	0.08	5.2	0.4	39.8	33.2	41.7	0.05	6.2	0.5	33.8	38.2	55.5	0.07	6.1	0.5	34.4	32.9

5	-	6	L	108.6	0.09	3.6	0.3	18.0	38.5	61.7	0.08	5.6	0.4	37.6	37.4	42.2	0.06	6.7	0.5	41.4	40.4	50.9	0.08	6.8	0.5	23.4	33.8
5	-	7.6	L	75.5	0.10	4.8	0.4	40.6	36.0	77.8	0.07	6.7	0.5	22.6	29.8	59.5	0.07	7.0	0.6	32.4	34.6	48.3	0.09	6.7	0.5	26.6	30.0
5	-	7.6	L	62.6	0.10	4.6	0.4	37.2	41.0	84.2	0.08	7.0	0.5	22.2	29.6	53.6	0.08	7.2	0.6	36.6	31.0	45.8	0.07	7.0	0.5	25.6	29.7
5	-	7.6	L	107.4	0.09	4.7	0.4	33.8	37.4	72.8	0.08	7.0	0.5	30.4	29.4	47.7	0.09	7.2	0.6	58.8	83.6	46.8	0.07	6.7	0.5	29.0	28.8
5	-	7.6	L	171.1	0.10	5.5	0.4	43.0	42.1	83.4	0.08	4.6	0.4	45.8	45.6	57.8	0.09	6.2	0.5	65.2	88.8	48.7	0.08	6.6	0.5	25.4	30.0
5	-	7.6	L	178.0	0.08	4.9	0.4	34.0	47.2	90.0	0.09	3.9	0.3	44.8	48.4	61.1	0.09	6.2	0.5	32.4	32.5	47.5	0.08	6.2	0.5	25.4	28.7
5	-	8.6	L	81.9	0.08	5.7	0.4	22.2	30.0	67.0	0.09	6.5	0.5	43.2	43.6	47.9	0.06	6.3	0.5	41.8	41.2	42.3	0.08	6.9	0.5	23.2	27.4
5	-	8.6	L	81.7	0.09	5.3	0.4	43.8	38.8	66.3	0.08	6.7	0.5	64.6	55.0	48.7	0.06	6.7	0.5	29.8	48.8	43.3	0.07	7.2	0.6	24.6	29.5
5	-	8.6	L	68.5	0.08	3.5	0.3	32.4	35.6	79.4	0.08	3.3	0.3	50.6	46.8	66.0	0.07	5.9	0.5	46.6	46.4	44.5	0.09	7.3	0.6	29.0	33.1
5	-	8.6	L	111.1	0.08	3.3	0.3	35.8	39.0	79.0	0.09	4.4	0.3	35.8	30.4	68.7	0.07	6.4	0.5	35.4	39.8	77.6	0.10	6.0	0.5	24.2	30.6
5	-	8.6	L	81.9	0.08	4.0	0.3	38.6	41.2	77.6	0.06	3.8	0.3	34.4	28.2	74.8	0.07	7.0	0.6	57.8	53.8	58.7	0.09	5.0	0.4	22.6	31.1
