# Supplementary material for: Extracellular Polymeric Substances (EPS) of Freshwater Biofilms Stabilize and Modify CeO2 and Ag Nanoparticles
Source: PLoS One. 2014 Oct 21;9(10):e110709. doi: 10.1371/journal.pone.0110709 (PMC4204993; doi:10.1371/journal.pone.0110709)
Supplement: Table S11 — Mean Z-averages (DLS), polydispersity (PDI), mode and mean diameters (NTA), zetapotential, and EPM of Ag NP dispersions dependent on pH, light/dark, EPS content, and time. Each value is derived from three measurements of three replicates (3x 3n). Blue: data used to calculate mean values represented in Figures 4 A–D. (PDF) [file pone.0110709.s019.pdf]

				3 h						24 h						168 h						336 h					
mg/L	EPS	pH	L/D	z-average	PDI	ZP	EPM	Mode	Mean	z-average	PDI	ZP	EPM	Mode	Mean	z-average	PDI	ZP	EPM	Mode	Mean	z-average	PDI	ZP	EPM	Mode	Mean
0.5	1	6	D	57.2	0.251	-19.3	-1.51	41	53	57.3	0.311	-21.1	-1.65	36	49	55.9	0.322	-18.1	-1.42	41	56	51.1	0.462	-22.7	-1.78	60	61
0.5	2	6	D	56.8	0.448	-19.2	-1.50	43	54	53.1	0.487	-20.4	-1.60	41	50	58.4	0.325	-20.4	-1.60	33	64	51.7	0.468	-22.6	-1.77	54	58
0.5	3	6	D	59.6	0.368	-21.0	-1.65	44	52	60.2	0.332	-17.8	-1.40	40	51	52.0	0.28	-16.8	-1.32	50	65	50.3	0.456	-18.0	-1.41	60	60
0.5	4	6	D	57.7	0.245	-17.8	-1.40	42	57	56.8	0.222	-17.9	-1.40	43	52	57.2	0.37	-15.6	-1.22	57	60	53.4	0.49	-18.1	-1.42	64	70
0.5	5	6	D	55.2	0.394	-19.8	-1.55	42	47	53.7	0.409	-23.5	-1.84	48	55	52.0	0.335	-18.2	-1.43	36	57	54.0	0.401	-21.3	-1.67	59	63
0.5	1	7.6	D	55.3	0.193	-17.8	-1.40	40	46	55.9	0.269	-22.6	-1.77	40	51	52.4	0.248	-17.2	-1.35	40	57	57.8	0.46	-22.2	-1.74	60	57
0.5	2	7.6	D	53.6	0.323	-20.4	-1.60	41	48	55.0	0.336	-25.2	-1.98	40	53	53.8	0.377	-16.7	-1.31	42	51	57.2	0.452	-22.2	-1.74	56	57
0.5	3	7.6	D	51.1	0.48	-18.5	-1.45	42	51	54.0	0.243	-26.2	-2.05	40	49	50.0	0.339	-19.1	-1.50	41	51	50.7	0.461	-17.2	-1.35	53	78
0.5	4	7.6	D	56.7	0.318	-18.9	-1.48	40	50	59.1	0.215	-17.7	-1.39	42	52	48.4	0.317	-19.0	-1.49	41	51	52.8	0.489	-18.6	-1.46	59	60
0.5	5	7.6	D	53.4	0.242	-20.0	-1.57	42	47	51.9	0.288	-23.4	-1.83	41	51	50.6	0.381	-18.7	-1.47	40	49	51.1	0.452	-18.0	-1.41	59	64
0.5	1	8.6	D	53.6	0.41	-20.4	-1.60	42	51	56.9	0.371	-20.5	-1.61	38	57	48.4	0.211	-20.2	-1.58	40	51	51.7	0.469	-23.7	-1.86	65	63
0.5	2	8.6	D	53.7	0.351	-20.9	-1.64	42	53	55.3	0.338	-21.2	-1.66	36	57	50.8	0.216	-21.7	-1.70	42	50	53.2	0.474	-26.0	-2.04	70	62
0.5	3	8.6	D	54.9	0.454	-18.6	-1.46	40	46	49.3	0.286	-20.6	-1.61	42	57	57.5	0.364	-18.5	-1.45	35	47	51.6	0.451	-16.0	-1.25	58	61
0.5	4	8.6	D	56.4	0.306	-20.0	-1.57	40	51	53.1	0.385	-17.9	-1.40	41	52	50.1	0.213	-18.6	-1.46	40	53	53.6	0.46	-18.1	-1.42	58	63
0.5	5	8.6	D	59.3	0.25	-21.2	-1.66	42	52	53.8	0.26	-23.3	-1.83	41	50	50.6	0.285	-19.6	-1.54	40	37	51.4	0.491	-17.5	-1.37	61	69
0.5	-	6	D	53.5	0.276	-19.4	-1.52	40	67	55.8	0.229	-23.8	-1.87	40	56	58.4	0.278	-19.7	-1.55	40	58	56.1	0.471	-29.9	-2.34	54	57
0.5	-	6	D	51.0	0.156	-20.1	-1.58	40	60	58.0	0.199	-18.8	-1.47	40	62	56.6	0.477	-20.6	-1.61	35	59	55.5	0.454	-29.3	-2.30	60	58
0.5	-	6	D	55.4	0.478	-20.6	-1.61	41	63	55.6	0.247	-14.0	-1.10	41	54	54.9	0.328	-17.6	-1.38	41	59	59.6	0.407	-25.5	-2.00	60	53
0.5	-	6	D	59.3	0.237	-19.3	-1.52	40	55	59.0	0.304	-17.3	-1.36	44	48	56.4	0.429	-19.9	-1.56	40	66	53.6	0.331	-22.0	-1.72	50	73
0.5	-	6	D	56.5	0.414	-13.3	-1.04	40	65	58.3	0.313	-15.2	-1.19	42	54	52.3	0.31	-20.0	-1.57	38	55	55.7	0.345	-22.3	-1.75	51	65
0.5	-	7.6	D	54.8	0.225	-19.2	-1.50	41	57	55.9	0.456	-18.8	-1.47	40	47	56.7	0.276	-16.6	-1.30	40	65	58.1	0.481	-25.5	-2.00	50	60
0.5	-	7.6	D	52.8	0.212	-21.5	-1.69	43	64	58.4	0.474	-19.8	-1.55	40	52	58.4	0.309	-17.4	-1.36	41	50	59.1	0.484	-27.4	-2.15	60	50
0.5	-	7.6	D	55.8	0.22	-22.8	-1.79	40	51	53.2	0.363	-23.9	-1.87	41	50	53.5	0.315	-18.8	-1.47	41	52	54.7	0.426	-25.0	-1.96	55	53
0.5	-	7.6	D	54.4	0.436	-20.6	-1.61	41	50	55.9	0.306	-15.6	-1.22	40	51	54.3	0.314	-19.5	-1.53	43	53	50.8	0.442	-25.8	-2.02	52	60
0.5	-	7.6	D	52.5	0.464	-22.6	-1.77	41	48	55.4	0.472	-16.1	-1.26	42	53	56.9	0.272	-20.0	-1.57	41	56	51.7	0.494	-28.2	-2.21	50	57
0.5	-	8.6	D	52.7	0.318	-18.3	-1.43	42	61	58.7	0.444	-18.8	-1.47	42	60	51.0	0.491	-24.7	-1.94	42	66	56.5	0.466	-26.8	-2.10	56	56
0.5	-	8.6	D	50.1	0.216	-19.4	-1.52	45	62	55.7	0.276	-23.4	-1.83	41	62	51.9	0.354	-28.8	-2.26	42	50	56.7	0.448	-26.5	-2.08	59	53

0.5	-	8.6	D	51.3	0.219	-20.3	-1.59	42	60	59.3	0.275	-16.4	-1.29	41	49	54.0	0.303	-26.5	-2.08	41	50	56.0	0.412	-21.8	-1.71	64	55
0.5	-	8.6	D	42.7	0.384	-23.3	-1.83	41	51	46.7	0.436	-15.6	-1.22	41	51	51.3	0.356	-19.2	-1.50	42	55	57.8	0.439	-27.4	-2.15	52	61
0.5	-	8.6	D	50.3	0.413	-17.2	-1.35	41	48	54.0	0.54	-15.6	-1.22	42	53	47.4	0.324	-23.0	-1.80	37	48	53.7	0.42	-19.6	-1.54	54	63
5	1	6	D	44.3	0.466	-20.5	-1.61	43	62	56.0	0.425	-17.5	-1.37	51	59	55.4	0.453	-23.4	-1.83	40	52	48.4	0.484	-23.5	-1.84	53	61
5	2	6	D	43.7	0.46	-20.0	-1.57	50	65	44.8	0.449	-18.2	-1.43	51	56	56.0	0.304	-25.0	-1.96	40	50	49.7	0.456	-23.5	-1.84	56	62
5	3	6	D	45.1	0.472	-21.2	-1.66	46	64	45.0	0.45	-19.1	-1.50	51	50	53.1	0.488	-19.6	-1.54	130	58	43.7	0.494	-18.5	-1.45	48	60
5	4	6	D	46.9	0.446	-22.2	-1.74	55	72	47.8	0.476	-21.4	-1.68	50	50	54.7	0.489	-21.3	-1.67	41	53	43.0	0.492	-21.6	-1.69	54	59
5	5	6	D	49.9	0.468	-22.3	-1.75	53	75	46.6	0.389	-19.5	-1.53	50	56	47.2	0.345	-21.9	-1.72	59	56	42.8	0.482	-19.2	-1.50	60	67
5	1	7.6	D	42.4	0.473	-19.4	-1.52	43	61	50.8	0.374	-18.9	-1.48	51	56	49.1	0.469	-18.8	-1.47	53	59	41.6	0.459	-21.9	-1.72	56	67
5	2	7.6	D	44.2	0.492	-20.4	-1.60	42	57	52.2	0.32	-20.6	-1.61	47	43	48.1	0.454	-19.1	-1.50	59	57	40.2	0.487	-22.1	-1.73	52	61
5	3	7.6	D	47.1	0.454	-21.9	-1.72	50	70	49.2	0.426	-18.1	-1.42	51	59	55.0	0.319	-19.4	-1.52	55	53	45.6	0.462	-16.6	-1.30	43	58
5	4	7.6	D	45.7	0.428	-17.0	-1.33	55	71	51.4	0.445	-18.0	-1.41	53	56	47.4	0.465	-20.7	-1.62	55	54	42.4	0.453	-17.6	-1.38	59	65
5	5	7.6	D	50.0	0.421	-17.4	-1.36	58	71	52.3	0.453	-16.7	-1.31	51	53	51.0	0.465	-22.1	-1.73	53	54	44.4	0.405	-24.7	-1.94	57	59
5	1	8.6	D	41.6	0.46	-21.2	-1.66	41	60	43.9	0.191	-22.6	-1.77	50	51	42.6	0.417	-23.7	-1.86	50	59	46.3	0.498	-23.9	-1.87	54	69
5	2	8.6	D	42.5	0.47	-21.9	-1.72	44	66	47.2	0.282	-20.0	-1.57	40	51	43.0	0.423	-20.5	-1.61	40	49	47.4	0.492	-23.7	-1.86	64	70
5	3	8.6	D	41.5	0.461	-22.3	-1.75	40	56	50.3	0.318	-20.9	-1.64	46	48	41.4	0.41	-24.6	-1.93	56	53	46.9	0.49	-24.3	-1.90	64	70
5	4	8.6	D	50.0	0.457	-23.3	-1.83	40	55	47.4	0.272	-14.2	-1.11	50	55	49.4	0.485	-18.1	-1.42	54	54	47.0	0.457	-15.7	-1.23	59	66
5	5	8.6	D	40.5	0.449	-18.8	-1.47	41	52	46.4	0.333	-17.7	-1.39	54	41	48.2	0.428	-18.3	-1.43	50	58	43.0	0.424	-16.0	-1.25	59	72
5	-	6	D	46.1	0.423	-25.1	-1.97	42	51	48.8	0.229	-22.4	-1.76	42	51	50.4	0.445	-28.3	-2.22	55	58	46.6	0.49	-30.9	-2.42	51	54
5	-	6	D	45.0	0.409	-29.7	-2.33	40	47	47.6	0.24	-23.7	-1.86	41	60	52.5	0.386	-23.6	-1.85	58	56	46.6	0.485	-31.8	-2.49	57	56
5	-	6	D	43.1	0.491	-25.5	-2.00	41	58	47.1	0.334	-27.6	-2.16	41	52	46.1	0.493	-23.9	-1.87	64	59	46.9	0.491	-25.2	-1.98	62	62
5	-	6	D	43.2	0.488	-32.2	-2.52	57	72	43.0	0.329	-23.0	-1.80	60	55	44.0	0.341	-27.4	-2.15	57	60	46.6	0.498	-25.2	-1.98	59	59
5	-	6	D	42.3	0.47	-31.1	-2.44	54	76	50.4	0.434	-22.9	-1.79	57	50	47.3	0.362	-26.0	-2.04	68	50	46.6	0.413	-27.5	-2.16	59	60
5	-	7.6	D	42.6	0.484	-32.7	-2.56	44	59	54.3	0.482	-29.6	-2.32	41	63	42.5	0.218	-23.5	-1.84	55	52	45.4	0.478	-30.4	-2.38	55	55
5	-	7.6	D	43.6	0.403	-33.5	-2.63	43	61	47.1	0.488	-32.9	-2.58	42	62	42.5	0.214	-25.4	-1.99	61	53	46.7	0.481	-28.5	-2.23	56	56
5	-	7.6	D	42.4	0.482	-33.4	-2.62	50	77	47.2	0.481	-29.3	-2.30	43	50	40.7	0.456	-27.9	-2.19	56	56	41.3	0.405	-23.3	-1.83	52	55
5	-	7.6	D	42.4	0.476	-33.8	-2.65	50	74	51.0	0.346	-19.2	-1.50	50	59	43.1	0.453	-28.2	-2.21	56	50	40.4	0.4	-27.8	-2.18	52	55
5	-	7.6	D	44.2	0.495	-36.4	-2.85	40	59	47.2	0.462	-26.5	-2.08	52	65	40.5	0.48	-23.5	-1.84	40	56	40.8	0.498	-33.4	-2.62	58	70
5	-	8.6	D	43.1	0.494	-34.3	-2.69	45	57	50.0	0.311	-21.3	-1.67	48	54	49.5	0.479	-23.1	-1.81	40	55	48.5	0.408	-27.3	-2.14	60	59

5	-	8.6	D	42.1	0.489	-31.2	-2.45	50	65	46.2	0.34	-21.4	-1.68	43	54	40.3	0.481	-24.3	-1.90	43	52	40.8	0.477	-28.6	-2.24	57	63
5	-	8.6	D	39.4	0.421	-30.9	-2.42	47	61	45.9	0.468	-13.5	-1.06	40	49	39.7	0.47	-24.9	-1.95	60	56	40.1	0.468	-29.9	-2.34	63	62
5	-	8.6	D	39.1	0.426	-33.3	-2.61	45	65	43.6	0.326	-15.2	-1.19	40	55	38.0	0.476	-17.5	-1.37	58	55	44.4	0.459	-25.6	-2.01	60	56
5	-	8.6	D	37.7	0.481	-19.3	-1.51	50	60	41.2	0.439	-15.3	-1.20	54	53	49.2	0.495	-18.5	-1.45	58	59	40.9	0.441	-27.3	-2.14	54	58
0.5	1	6	L	54.9	0.215	-16.0	-1.26	42	60	50.6	0.476	-18.5	-1.45	57	59	188.2	0.46	-16.9	-1.32	87	138	189.0	0.311	-18.6	-1.46	128	178
0.5	2	6	L	56.9	0.362	-16.5	-1.29	41	52	54.7	0.48	-17.9	-1.40	42	68	175.4	0.436	-16.4	-1.29	89	142	189.6	0.322	-19.3	-1.51	128	190
0.5	3	6	L	51.7	0.252	-20.9	-1.64	40	59	57.0	0.426	-16.6	-1.30	60	52	184.2	0.446	-16.3	-1.28	90	134	177.6	0.395	-19.9	-1.56	112	176
0.5	4	6	L	53.4	0.47	-19.7	-1.54	38	53	54.1	0.352	-14.7	-1.15	75	53	58.0	0.423	-19.7	-1.54	47	50	226.2	0.421	-15.5	-1.21	104	173
0.5	5	6	L	51.1	0.493	-19.0	-1.49	40	55	53.1	0.307	-29.6	-2.32	63	60	187.5	0.458	-17.1	-1.34	67	166	185.8	0.419	-17.2	-1.35	118	175
0.5	1	7.6	L	55.3	0.404	-15.2	-1.19	41	60	51.7	0.457	-15.0	-1.18	45	51	92.9	0.453	-15.3	-1.20	62	117	85.4	0.437	-22.3	-1.75	64	93
0.5	2	7.6	L	54.4	0.369	-16.8	-1.32	45	61	56.8	0.365	-16.9	-1.32	44	58	92.4	0.363	-16.3	-1.28	51	127	86.2	0.446	-24.2	-1.90	62	115
0.5	3	7.6	L	59.3	0.317	-22.9	-1.79	40	53	50.7	0.347	-17.0	-1.33	46	57	99.4	0.475	-18.0	-1.41	61	113	99.4	0.468	-16.4	-1.29	63	119
0.5	4	7.6	L	53.8	0.223	-15.1	-1.18	40	62	50.3	0.469	-19.2	-1.51	42	51	58.0	0.31	-19.4	-1.52	50	56	88.0	0.494	-17.4	-1.36	56	90
0.5	5	7.6	L	54.5	0.341	-18.8	-1.48	40	55	57.2	0.446	-23.8	-1.86	43	52	97.4	0.409	-19.7	-1.54	60	125	88.8	0.45	-19.8	-1.55	50	72
0.5	1	8.6	L	57.0	0.398	-16.2	-1.27	46	51	58.2	0.328	-15.4	-1.21	42	60	59.2	0.406	-17.0	-1.33	50	58	56.4	0.44	-23.0	-1.80	61	76
0.5	2	8.6	L	53.3	0.303	-20.4	-1.60	47	55	58.0	0.359	-12.8	-1.00	45	55	62.6	0.459	-21.6	-1.69	52	52	56.8	0.452	-25.5	-2.00	52	78
0.5	3	8.6	L	56.9	0.357	-18.0	-1.41	34	57	45.8	0.457	-20.4	-1.60	42	53	50.2	0.454	-21.4	-1.68	54	51	56.9	0.446	-25.0	-1.96	58	84
0.5	4	8.6	L	59.4	0.344	-19.8	-1.55	43	52	52.9	0.32	-18.9	-1.48	40	52	57.8	0.422	-22.6	-1.77	56	50	52.3	0.356	-17.0	-1.33	49	72
0.5	5	8.6	L	52.2	0.226	-15.6	-1.22	40	52	53.3	0.354	-18.5	-1.45	41	52	58.2	0.475	-24.2	-1.90	43	54	52.7	0.337	-18.8	-1.47	52	78
0.5	-	6	L	55.9	0.275	-19.6	-1.54	69	53	50.4	0.395	-18.3	-1.43	46	51	58.3	0.33	-22.1	-1.73	48	57	57.5	0.471	-24.7	-1.94	60	63
0.5	-	6	L	57.1	0.407	-18.1	-1.41	53	56	51.1	0.461	-19.4	-1.52	45	54	58.4	0.476	-20.7	-1.62	37	47	58.8	0.475	-25.7	-2.01	66	65
0.5	-	6	L	53.4	0.257	-18.5	-1.45	36	50	54.8	0.407	-15.1	-1.18	40	61	58.8	0.451	-18.1	-1.42	34	59	56.3	0.468	-20.6	-1.61	54	70
0.5	-	6	L	55.2	0.49	-19.3	-1.52	40	56	49.3	0.397	-15.5	-1.22	40	51	53.9	0.465	-21.5	-1.69	38	49	49.4	0.452	-22.8	-1.79	57	57
0.5	-	6	L	55.6	0.427	-19.5	-1.53	41	61	49.6	0.217	-15.0	-1.18	40	63	50.8	0.278	-25.7	-2.01	36	56	52.0	0.488	-24.4	-1.91	55	58
0.5	-	7.6	L	55.3	0.166	-18.2	-1.43	40	46	56.0	0.446	-18.3	-1.43	40	45	49.4	0.152	-22.4	-1.76	40	55	51.2	0.48	-24.3	-1.90	56	60
0.5	-	7.6	L	56.6	0.29	-17.7	-1.39	40	51	57.5	0.462	-16.2	-1.27	40	62	52.6	0.384	-22.4	-1.76	41	48	58.7	0.31	-26.3	-2.06	55	57
0.5	-	7.6	L	55.8	0.427	-17.5	-1.37	45	56	51.5	0.376	-18.4	-1.44	40	52	52.2	0.418	-22.9	-1.79	42	57	54.9	0.4	-22.3	-1.75	60	61
0.5	-	7.6	L	49.7	0.432	-19.1	-1.49	41	46	46.5	0.423	-18.2	-1.42	37	46	57.9	0.383	-20.6	-1.61	41	52	52.2	0.323	-25.1	-1.97	51	60
0.5	-	7.6	L	50.4	0.314	-19.6	-1.54	42	50	53.6	0.292	-18.0	-1.41	40	49	48.5	0.359	-24.3	-1.90	35	45	48.1	0.358	-22.2	-1.74	45	50

0.5	-	8.6	L	54.0	0.183	-19.4	-1.52	40	48	51.9	0.454	-18.8	-1.47	42	51	52.8	0.177	-20.8	-1.63	40	45	56.3	0.456	-27.8	-2.18	53	61
0.5	-	8.6	L	53.2	0.184	-19.9	-1.56	43	51	55.4	0.311	-18.7	-1.47	40	46	55.4	0.386	-24.8	-1.94	40	47	56.1	0.449	-25.7	-2.01	56	63
0.5	-	8.6	L	56.5	0.176	-19.5	-1.53	43	48	56.3	0.456	-16.8	-1.31	42	52	50.4	0.452	-26.7	-2.09	40	47	49.6	0.2	-27.3	-2.14	65	69
0.5	-	8.6	L	48.1	0.246	-19.0	-1.49	41	48	53.5	0.397	-20.5	-1.61	41	48	58.0	0.258	-23.6	-1.85	40	49	54.1	0.46	-25.9	-2.03	45	56
0.5	-	8.6	L	53.9	0.477	-19.3	-1.51	42	50	55.5	0.377	-16.4	-1.28	41	46	50.6	0.273	-25.1	-1.97	40	52	59.5	0.403	-29.5	-2.31	51	53
5	1	6	L	45.0	0.411	-20.6	-1.61	43	63	38.2	0.437	-20.6	-1.61	51	50	169.0	0.391	-23.6	-1.85	72	151	185.1	0.446	-25.5	-2.00	154	183
5	2	6	L	43.7	0.455	-22.6	-1.77	50	64	38.6	0.444	-20.2	-1.58	54	54	171.7	0.398	-25.1	-1.97	70	147	181.0	0.392	-26.1	-2.05	122	184
5	3	6	L	46.1	0.382	-23.1	-1.81	52	56	38.7	0.457	-22.8	-1.78	42	64	171.4	0.386	-26.0	-2.04	61	150	190.7	0.368	-21.9	-1.72	206	187
5	4	6	L	45.3	0.497	-19.1	-1.50	44	53	41.5	0.432	-23.3	-1.83	44	52	59.5	0.215	-23.5	-1.84	62	150	216.3	0.41	-21.9	-1.72	152	189
5	5	6	L	44.6	0.492	-21.2	-1.66	50	54	39.1	0.469	-24.0	-1.88	41	59	164.2	0.26	-21.9	-1.72	67	147	181.9	0.378	-23.5	-1.84	156	185
5	1	7.6	L	41.6	0.439	-19.3	-1.51	43	56	41.5	0.448	-21.3	-1.67	50	50	76.9	0.434	-25.0	-1.96	51	102	81.2	0.446	-22.3	-1.75	67	99
5	2	7.6	L	42.5	0.444	-18.9	-1.48	40	61	42.3	0.458	-22.2	-1.74	43	55	81.0	0.446	-25.4	-1.99	54	104	80.3	0.484	-23.0	-1.80	76	96
5	3	7.6	L	41.6	0.432	-19.5	-1.53	43	66	40.7	0.432	-22.1	-1.73	43	57	78.6	0.429	-24.7	-1.94	63	91	80.2	0.433	-21.8	-1.71	74	95
5	4	7.6	L	42.0	0.478	-16.6	-1.30	51	52	42.8	0.468	-21.9	-1.72	50	55	47.0	0.25	-20.4	-1.60	60	100	88.9	0.491	-14.8	-1.16	60	90
5	5	7.6	L	42.7	0.475	-17.0	-1.33	47	53	43.2	0.47	-14.2	-1.11	41	50	91.9	0.327	-20.2	-1.58	61	100	81.6	0.454	-14.7	-1.15	70	85
5	1	8.6	L	40.5	0.314	-21.4	-1.68	41	53	37.5	0.454	-22.9	-1.79	40	56	59.4	0.435	-22.5	-1.76	51	79	42.2	0.464	-22.3	-1.75	73	70
5	2	8.6	L	47.6	0.466	-28.6	-2.24	45	57	37.8	0.445	-22.6	-1.77	43	52	67.2	0.511	-22.7	-1.78	46	88	41.9	0.457	-24.3	-1.90	66	75
5	3	8.6	L	52.1	0.452	-21.6	-1.69	44	59	41.3	0.416	-18.0	-1.41	45	53	67.9	0.467	-22.8	-1.79	61	77	56.0	0.482	-18.3	-1.43	63	96
5	4	8.6	L	48.0	0.476	-21.3	-1.67	51	59	41.2	0.422	-22.1	-1.73	40	58	44.3	0.295	-20.5	-1.61	52	87	58.0	0.489	-17.6	-1.38	69	79
5	5	8.6	L	50.1	0.358	-19.6	-1.54	56	50	40.6	0.434	-18.4	-1.44	45	57	68.7	0.495	-22.3	-1.75	59	86	57.5	0.482	-17.8	-1.40	68	87
5	-	6	L	43.5	0.355	-21.4	-1.68	44	66	43.4	0.404	-17.2	-1.35	43	51	57.4	0.246	-25.5	-2.00	40	49	35.8	0.459	-20.2	-1.58	59	58
5	-	6	L	44.7	0.354	-22.0	-1.72	44	65	41.7	0.446	-19.9	-1.56	41	66	51.4	0.347	-24.2	-1.90	40	54	33.7	0.499	-22.2	-1.74	56	59
5	-	6	L	46.1	0.403	-24.8	-1.94	41	57	44.0	0.413	-18.7	-1.47	42	59	49.8	0.308	-25.1	-1.97	38	62	36.2	0.469	-22.1	-1.73	51	59
5	-	6	L	40.5	0.459	-29.1	-2.28	52	68	53.4	0.446	-19.5	-1.53	42	54	40.6	0.419	-25.4	-1.99	42	56	56.9	0.454	-20.9	-1.64	57	62
5	-	6	L	41.9	0.47	-24.9	-1.95	54	54	51.0	0.485	-24.1	-1.89	40	53	45.8	0.228	-21.2	-1.66	40	50	58.1	0.449	-23.9	-1.87	59	54
5	-	7.6	L	39.4	0.428	-14.6	-1.14	43	52	42.9	0.465	-21.6	-1.69	42	60	39.2	0.451	-21.7	-1.70	40	52	41.6	0.498	-25.1	-1.97	62	60
5	-	7.6	L	36.9	0.455	-20.5	-1.61	41	52	44.0	0.435	-20.4	-1.60	44	63	39.2	0.459	-23.7	-1.86	40	53	42.7	0.452	-22.6	-1.77	66	63
5	-	7.6	L	39.9	0.471	-20.6	-1.61	41	49	43.0	0.471	-21.6	-1.69	45	60	40.2	0.312	-25.1	-1.97	43	57	43.6	0.463	-24.1	-1.89	59	58
5	-	7.6	L	38.8	0.46	-25.1	-1.97	49	56	44.0	0.462	-20.9	-1.64	43	54	43.7	0.471	-20.9	-1.64	44	55	49.2	0.368	-25.9	-2.03	54	57

5	-	7.6	L	39.8	0.468	-26.0	-2.04	55	58	40.2	0.34	-18.7	-1.46	40	63	45.2	0.33	-24.9	-1.95	41	74	55.7	0.495	-23.5	-1.84	58	54
5	-	8.6	L	41.0	0.463	-26.1	-2.05	40	51	41.7	0.381	-24.9	-1.95	46	61	47.0	0.267	-23.1	-1.81	44	56	40.4	0.48	-24.0	-1.88	62	59
5	-	8.6	L	39.2	0.432	-27.0	-2.12	40	53	49.7	0.363	-19.9	-1.56	43	63	43.7	0.447	-25.8	-2.02	50	64	38.9	0.406	-26.6	-2.08	71	63
5	-	8.6	L	39.9	0.43	-22.1	-1.73	41	56	35.4	0.473	-24.2	-1.90	45	60	42.2	0.432	-24.9	-1.95	42	60	44.6	0.432	-23.0	-1.80	55	58
5	-	8.6	L	41.4	0.466	-21.0	-1.65	45	64	36.4	0.487	-24.6	-1.93	42	57	42.1	0.488	-24.3	-1.90	43	51	45.8	0.477	-27.3	-2.14	57	54
5	-	8.6	L	41.4	0.474	-21.4	-1.68	41	60	35.8	0.4	-24.8	-1.94	36	54	35.5	0.46	-27.1	-2.12	40	52	43.5	0.438	-29.6	-2.32	61	54
