# Supplementary material for: Extracellular Polymeric Substances (EPS) of Freshwater Biofilms Stabilize and Modify CeO2 and Ag Nanoparticles
Source: PLoS One. 2014 Oct 21;9(10):e110709. doi: 10.1371/journal.pone.0110709 (PMC4204993; doi:10.1371/journal.pone.0110709)
Supplement: Table S12 — Standard deviations of Z-averages (DLS), polydispersity (PDI), mode and mean diameters (NTA), zetapotential, and EPM of Ag NP dispersions dependent on pH, light/dark, EPS content, and time. (PDF) [file pone.0110709.s020.pdf]

				3 h						24 h						168 h						336 h					
mg/L	EPS	pH	L/D	z-average	PDI	ZP	EPM	Mode	Mean	z-average	PDI	ZP	EPM	Mode	Mean	z-average	PDI	ZP	EPM	Mode	Mean	z-average	PDI	ZP	EPM	Mode	Mean
0.5	1	6	D	8.6	0.04	2.9	0.23	6.2	7.9	8.6	0.05	3.2	0.25	5.4	7.3	8.4	0.05	2.7	0.21	6.2	8.4	7.7	0.07	3.4	0.27	9.0	9.1
0.5	2	6	D	8.5	0.07	2.9	0.23	6.5	8.1	8.0	0.07	3.1	0.24	6.2	7.5	8.8	0.05	3.1	0.24	5.0	9.5	7.8	0.07	3.4	0.27	8.1	8.7
0.5	3	6	D	8.9	0.06	3.2	0.25	6.6	7.7	9.0	0.05	2.7	0.21	6.0	7.7	7.8	0.04	2.5	0.20	7.5	9.8	7.5	0.07	2.7	0.21	9.0	9.0
0.5	4	6	D	8.7	0.04	2.7	0.21	6.3	8.6	8.5	0.03	2.7	0.21	6.5	7.7	8.6	0.06	2.3	0.18	8.6	9.0	8.0	0.07	2.7	0.21	9.6	10.5
0.5	5	6	D	8.3	0.06	3.0	0.23	6.3	7.1	8.1	0.06	3.5	0.28	7.2	8.3	7.8	0.05	2.7	0.21	5.4	8.5	8.1	0.06	3.2	0.25	8.9	9.4
0.5	1	7.6	D	8.3	0.03	2.7	0.21	6.0	6.9	8.4	0.04	3.4	0.27	6.0	7.6	7.9	0.04	2.6	0.20	6.0	8.6	8.7	0.07	3.3	0.26	9.0	8.5
0.5	2	7.6	D	8.0	0.05	3.1	0.24	6.2	7.1	8.2	0.05	3.8	0.30	6.0	7.9	8.1	0.06	2.5	0.20	6.3	7.7	8.6	0.07	3.3	0.26	8.4	8.5
0.5	3	7.6	D	7.7	0.07	2.8	0.22	6.3	7.7	8.1	0.04	3.9	0.31	6.0	7.3	7.5	0.05	2.9	0.22	6.2	7.6	7.6	0.07	2.6	0.20	8.0	11.7
0.5	4	7.6	D	8.5	0.05	2.8	0.22	6.0	7.4	8.9	0.03	2.7	0.21	6.3	7.7	7.3	0.05	2.9	0.22	6.2	7.7	7.9	0.07	2.8	0.22	8.9	9.0
0.5	5	7.6	D	8.0	0.04	3.0	0.23	6.3	7.1	7.8	0.04	3.5	0.27	6.2	7.6	7.6	0.06	2.8	0.22	6.0	7.4	7.7	0.07	2.7	0.21	8.9	9.6
0.5	1	8.6	D	8.0	0.06	3.1	0.24	6.3	7.7	8.5	0.06	3.1	0.24	5.7	8.6	7.3	0.03	3.0	0.24	6.0	7.6	7.8	0.07	3.6	0.28	9.8	9.4
0.5	2	8.6	D	8.1	0.05	3.1	0.25	6.3	8.0	8.3	0.05	3.2	0.25	5.4	8.5	7.6	0.03	3.3	0.26	6.3	7.5	8.0	0.07	3.9	0.31	10.5	9.4
0.5	3	8.6	D	8.2	0.07	2.8	0.22	6.0	6.9	7.4	0.04	3.1	0.24	6.3	8.5	8.6	0.05	2.8	0.22	5.3	7.1	7.7	0.07	2.4	0.19	8.7	9.2
0.5	4	8.6	D	8.5	0.05	3.0	0.24	6.0	7.7	8.0	0.06	2.7	0.21	6.2	7.8	7.5	0.03	2.8	0.22	6.0	7.9	8.0	0.07	2.7	0.21	8.7	9.4
0.5	5	8.6	D	8.9	0.04	3.2	0.25	6.3	7.7	8.1	0.04	3.5	0.27	6.2	7.4	7.6	0.04	2.9	0.23	6.0	5.6	7.7	0.07	2.6	0.21	9.2	10.3
0.5	-	6	D	8.0	0.04	2.9	0.23	6.0	10.1	8.4	0.03	3.6	0.28	6.0	8.4	8.8	0.04	3.0	0.23	6.0	8.7	8.4	0.07	4.5	0.35	8.1	8.5
0.5	-	6	D	7.7	0.02	3.0	0.24	6.0	9.0	8.7	0.03	2.8	0.22	6.0	9.3	8.5	0.07	3.1	0.24	5.3	8.9	8.3	0.07	4.4	0.34	9.0	8.8
0.5	-	6	D	8.3	0.07	3.1	0.24	6.2	9.5	8.3	0.04	2.1	0.16	6.2	8.1	8.2	0.05	2.6	0.21	6.2	8.9	8.9	0.06	3.8	0.30	9.0	8.0
0.5	-	6	D	8.9	0.04	2.9	0.23	6.0	8.3	8.8	0.05	2.6	0.20	6.6	7.2	8.5	0.06	3.0	0.23	6.0	9.9	8.0	0.05	3.3	0.26	7.5	11.0
0.5	-	6	D	8.5	0.06	2.0	0.16	6.0	9.8	8.7	0.05	2.3	0.18	6.3	8.1	7.8	0.05	3.0	0.24	5.7	8.3	8.3	0.05	3.3	0.26	7.7	9.7
0.5	-	7.6	D	8.2	0.03	2.9	0.23	6.2	8.6	8.4	0.07	2.8	0.22	6.0	7.1	8.5	0.04	2.5	0.20	6.0	9.8	8.7	0.07	3.8	0.30	7.5	9.0

0.5	-	7.6	D	7.9	0.03	3.2	0.25	6.5	9.6	8.8	0.07	3.0	0.23	6.0	7.8	8.8	0.05	2.6	0.20	6.2	7.5	8.9	0.07	4.1	0.32	9.0	7.5
0.5	-	7.6	D	8.4	0.03	3.4	0.27	6.0	7.7	8.0	0.05	3.6	0.28	6.2	7.5	8.0	0.05	2.8	0.22	6.2	7.8	8.2	0.06	3.8	0.29	8.3	8.0
0.5	-	7.6	D	8.2	0.07	3.1	0.24	6.2	7.5	8.4	0.05	2.3	0.18	6.0	7.7	8.1	0.05	2.9	0.23	6.5	8.0	7.6	0.07	3.9	0.30	7.8	9.0
0.5	-	7.6	D	7.9	0.07	3.4	0.27	6.2	7.2	8.3	0.07	2.4	0.19	6.3	8.0	8.5	0.04	3.0	0.24	6.2	8.4	7.8	0.07	4.2	0.33	7.5	8.6
0.5	-	8.6	D	7.9	0.05	2.7	0.22	6.3	9.2	8.8	0.07	2.8	0.22	6.3	9.0	7.7	0.07	3.7	0.29	6.3	9.9	8.5	0.07	4.0	0.32	8.4	8.4
0.5	-	8.6	D	7.5	0.03	2.9	0.23	6.8	9.3	8.4	0.04	3.5	0.28	6.2	9.3	7.8	0.05	4.3	0.34	6.3	7.5	8.5	0.07	4.0	0.31	8.9	8.0
0.5	-	8.6	D	7.7	0.03	3.0	0.24	6.3	9.0	8.9	0.04	2.5	0.19	6.2	7.4	8.1	0.05	4.0	0.31	6.2	7.5	8.4	0.06	3.3	0.26	9.6	8.3
0.5	-	8.6	D	6.4	0.06	3.5	0.27	6.2	7.7	7.0	0.07	2.3	0.18	6.2	7.7	7.7	0.05	2.9	0.23	6.3	8.3	8.7	0.07	4.1	0.32	7.8	9.1
0.5	-	8.6	D	7.5	0.06	2.6	0.20	6.2	7.2	8.1	0.08	2.3	0.18	6.3	8.0	7.1	0.05	3.5	0.27	5.6	7.2	8.0	0.06	2.9	0.23	8.1	9.4
5	1	6	D	6.6	0.07	3.1	0.24	6.5	9.3	8.4	0.06	2.6	0.21	7.7	8.9	8.3	0.07	3.5	0.28	6.0	7.8	7.3	0.07	3.5	0.28	8.0	9.2
5	2	6	D	6.6	0.07	3.0	0.24	7.5	9.8	6.7	0.07	2.7	0.21	7.7	8.4	8.4	0.05	3.8	0.29	6.0	7.5	7.5	0.07	3.5	0.28	8.4	9.3
5	3	6	D	6.8	0.07	3.2	0.25	6.9	9.6	6.7	0.07	2.9	0.22	7.7	7.5	8.0	0.07	2.9	0.23	19.5	8.7	6.6	0.07	2.8	0.22	7.2	8.9
5	4	6	D	7.0	0.07	3.3	0.26	8.3	10.8	7.2	0.07	3.2	0.25	7.5	7.5	8.2	0.07	3.2	0.25	6.2	8.0	6.4	0.07	3.2	0.25	8.1	8.9
5	5	6	D	7.5	0.07	3.3	0.26	8.0	11.2	7.0	0.06	2.9	0.23	7.5	8.4	7.1	0.05	3.3	0.26	8.9	8.4	6.4	0.07	2.9	0.23	9.0	10.0
5	1	7.6	D	6.4	0.07	2.9	0.23	6.5	9.2	7.6	0.06	2.8	0.22	7.7	8.4	7.4	0.07	2.8	0.22	8.0	8.9	6.2	0.07	3.3	0.26	8.4	10.1
5	2	7.6	D	6.6	0.07	3.1	0.24	6.3	8.6	7.8	0.05	3.1	0.24	7.1	6.5	7.2	0.07	2.9	0.22	8.9	8.5	6.0	0.07	3.3	0.26	7.8	9.1
5	3	7.6	D	7.1	0.07	3.3	0.26	7.5	10.5	7.4	0.06	2.7	0.21	7.7	8.9	8.2	0.05	2.9	0.23	8.3	8.0	6.8	0.07	2.5	0.20	6.5	8.7
5	4	7.6	D	6.9	0.06	2.6	0.20	8.3	10.6	7.7	0.07	2.7	0.21	8.0	8.4	7.1	0.07	3.1	0.24	8.3	8.1	6.4	0.07	2.6	0.21	8.9	9.8
5	5	7.6	D	7.5	0.06	2.6	0.20	8.7	10.6	7.8	0.07	2.5	0.20	7.7	8.0	7.6	0.07	3.3	0.26	8.0	8.1	6.7	0.06	3.7	0.29	8.6	8.9
5	1	8.6	D	6.2	0.07	3.2	0.25	6.2	9.0	6.6	0.03	3.4	0.27	7.5	7.7	6.4	0.06	3.6	0.28	7.5	8.9	6.9	0.07	3.6	0.28	8.1	10.4
5	2	8.6	D	6.4	0.07	3.3	0.26	6.6	9.9	7.1	0.04	3.0	0.23	6.0	7.7	6.5	0.06	3.1	0.24	6.0	7.4	7.1	0.07	3.6	0.28	9.6	10.6
5	3	8.6	D	6.2	0.07	3.3	0.26	6.0	8.4	7.5	0.05	3.1	0.25	6.9	7.2	6.2	0.06	3.7	0.29	8.4	8.0	7.0	0.07	3.6	0.29	9.6	10.5

5	4	8.6	D	7.5	0.07	3.5	0.27	6.0	8.3	7.1	0.04	2.1	0.17	7.5	8.3	7.4	0.07	2.7	0.21	8.1	8.2	7.1	0.07	2.4	0.18	8.9	9.9
5	5	8.6	D	6.1	0.07	2.8	0.22	6.2	7.8	7.0	0.05	2.7	0.21	8.1	6.2	7.2	0.06	2.7	0.22	7.5	8.7	6.5	0.06	2.4	0.19	8.9	10.8
5	-	6	D	6.9	0.06	3.8	0.30	6.3	7.7	7.3	0.03	3.4	0.26	6.3	7.7	7.6	0.07	4.2	0.33	8.3	8.6	7.0	0.07	4.6	0.36	7.7	8.1
5	-	6	D	6.7	0.06	4.5	0.35	6.0	7.1	7.1	0.04	3.6	0.28	6.2	9.0	7.9	0.06	3.5	0.28	8.7	8.4	7.0	0.07	4.8	0.37	8.6	8.4
5	-	6	D	6.5	0.07	3.8	0.30	6.2	8.7	7.1	0.05	4.1	0.32	6.2	7.8	6.9	0.07	3.6	0.28	9.6	8.8	7.0	0.07	3.8	0.30	9.3	9.2
5	-	6	D	6.5	0.07	4.8	0.38	8.6	10.8	6.5	0.05	3.5	0.27	9.0	8.3	6.6	0.05	4.1	0.32	8.6	9.0	7.0	0.07	3.8	0.30	8.9	8.9
5	-	6	D	6.4	0.07	4.7	0.37	8.1	11.4	7.6	0.07	3.4	0.27	8.6	7.5	7.1	0.05	3.9	0.31	10.2	7.5	7.0	0.06	4.1	0.32	8.9	9.0
5	-	7.6	D	6.4	0.07	4.9	0.38	6.6	8.9	8.1	0.07	4.4	0.35	6.2	9.5	6.4	0.03	3.5	0.28	8.3	7.8	6.8	0.07	4.6	0.36	8.3	8.3
5	-	7.6	D	6.5	0.06	5.0	0.39	6.5	9.2	7.1	0.07	4.9	0.39	6.3	9.3	6.4	0.03	3.8	0.30	9.2	8.0	7.0	0.07	4.3	0.34	8.4	8.3
5	-	7.6	D	6.4	0.07	5.0	0.39	7.5	11.6	7.1	0.07	4.4	0.34	6.5	7.5	6.1	0.07	4.2	0.33	8.4	8.5	6.2	0.06	3.5	0.27	7.8	8.2
5	-	7.6	D	6.4	0.07	5.1	0.40	7.5	11.1	7.7	0.05	2.9	0.23	7.5	8.9	6.5	0.07	4.2	0.33	8.4	7.5	6.1	0.06	4.2	0.33	7.8	8.2
5	-	7.6	D	6.6	0.07	5.5	0.43	6.0	8.9	7.1	0.07	4.0	0.31	7.8	9.8	6.1	0.07	3.5	0.28	6.0	8.4	6.1	0.07	5.0	0.39	8.7	10.5
5	-	8.6	D	6.5	0.07	5.1	0.40	6.8	8.6	7.5	0.05	3.2	0.25	7.2	8.1	7.4	0.07	3.5	0.27	6.0	8.3	7.3	0.06	4.1	0.32	9.0	8.9
5	-	8.6	D	6.3	0.07	4.7	0.37	7.5	9.8	6.9	0.05	3.2	0.25	6.5	8.1	6.1	0.07	3.6	0.29	6.5	7.8	6.1	0.07	4.3	0.34	8.6	9.5
5	-	8.6	D	5.9	0.06	4.6	0.36	7.1	9.2	6.9	0.07	2.0	0.16	6.0	7.4	6.0	0.07	3.7	0.29	9.0	8.5	6.0	0.07	4.5	0.35	9.5	9.4
5	-	8.6	D	5.9	0.06	5.0	0.39	6.8	9.8	6.5	0.05	2.3	0.18	6.0	8.3	5.7	0.07	2.6	0.21	8.7	8.3	6.7	0.07	3.8	0.30	9.0	8.4
5	-	8.6	D	5.7	0.07	2.9	0.23	7.5	9.0	6.2	0.07	2.3	0.18	8.1	8.0	7.4	0.07	2.8	0.22	8.7	8.9	6.1	0.07	4.1	0.32	8.1	8.7
0.5	1	6	L	8.2	0.03	2.4	0.19	6.3	9.0	7.6	0.07	2.8	0.22	8.6	8.9	28.2	0.07	2.5	0.20	13.1	20.7	28.4	0.05	2.8	0.22	19.2	26.6
0.5	2	6	L	8.5	0.05	2.5	0.19	6.2	7.8	8.2	0.07	2.7	0.21	6.3	10.2	26.3	0.07	2.5	0.19	13.4	21.3	28.4	0.05	2.9	0.23	19.2	28.5
0.5	3	6	L	7.8	0.04	3.1	0.25	6.0	8.9	8.6	0.06	2.5	0.20	9.0	7.8	27.6	0.07	2.4	0.19	13.5	20.1	26.6	0.06	3.0	0.23	16.8	26.4
0.5	4	6	L	8.0	0.07	3.0	0.23	5.7	8.0	8.1	0.05	2.2	0.17	11.3	8.0	8.7	0.06	3.0	0.23	7.1	7.5	33.9	0.06	2.3	0.18	15.6	26.0
0.5	5	6	L	7.7	0.07	2.9	0.22	6.0	8.3	8.0	0.05	4.4	0.35	9.5	9.0	28.1	0.07	2.6	0.20	10.1	24.9	27.9	0.06	2.6	0.20	17.7	26.3

0.5	1	7.6	L	8.3	0.06	2.3	0.18	6.2	9.0	7.8	0.07	2.3	0.18	6.8	7.7	13.9	0.07	2.3	0.18	9.3	17.6	12.8	0.07	3.3	0.26	9.6	14.0
0.5	2	7.6	L	8.2	0.06	2.5	0.20	6.8	9.2	8.5	0.05	2.5	0.20	6.6	8.7	13.9	0.05	2.4	0.19	7.7	19.1	12.9	0.07	3.6	0.28	9.3	17.2
0.5	3	7.6	L	8.9	0.05	3.4	0.27	6.0	8.0	7.6	0.05	2.6	0.20	6.9	8.6	14.9	0.07	2.7	0.21	9.2	17.0	14.9	0.07	2.5	0.19	9.5	17.9
0.5	4	7.6	L	8.1	0.03	2.3	0.18	6.0	9.3	7.5	0.07	2.9	0.23	6.3	7.7	8.7	0.05	2.9	0.23	7.5	8.4	13.2	0.07	2.6	0.20	8.4	13.5
0.5	5	7.6	L	8.2	0.05	2.8	0.22	6.0	8.3	8.6	0.07	3.6	0.28	6.5	7.8	14.6	0.06	3.0	0.23	9.0	18.8	13.3	0.07	3.0	0.23	7.5	10.7
0.5	1	8.6	L	8.5	0.06	2.4	0.19	6.9	7.7	8.7	0.05	2.3	0.18	6.3	9.0	8.9	0.06	2.6	0.20	7.5	8.7	8.5	0.07	3.5	0.27	9.2	11.4
0.5	2	8.6	L	8.0	0.05	3.1	0.24	7.1	8.3	8.7	0.05	1.9	0.15	6.8	8.3	9.4	0.07	3.2	0.25	7.8	7.8	8.5	0.07	3.8	0.30	7.8	11.7
0.5	3	8.6	L	8.5	0.05	2.7	0.21	5.1	8.6	6.9	0.07	3.1	0.24	6.3	8.0	7.5	0.07	3.2	0.25	8.1	7.7	8.5	0.07	3.8	0.29	8.7	12.6
0.5	4	8.6	L	8.9	0.05	3.0	0.23	6.5	7.8	7.9	0.05	2.8	0.22	6.0	7.8	8.7	0.06	3.4	0.27	8.4	7.5	7.8	0.05	2.6	0.20	7.4	10.9
0.5	5	8.6	L	7.8	0.03	2.3	0.18	6.0	7.8	8.0	0.05	2.8	0.22	6.2	7.8	8.7	0.07	3.6	0.28	6.5	8.1	7.9	0.05	2.8	0.22	7.8	11.7
0.5	-	6	L	8.4	0.04	2.9	0.23	10.4	8.0	7.6	0.06	2.7	0.22	6.9	7.7	8.7	0.05	3.3	0.26	7.2	8.6	8.6	0.07	3.7	0.29	9.0	9.5
0.5	-	6	L	8.6	0.06	2.7	0.21	8.0	8.4	7.7	0.07	2.9	0.23	6.8	8.1	8.8	0.07	3.1	0.24	5.6	7.1	8.8	0.07	3.9	0.30	9.9	9.7
0.5	-	6	L	8.0	0.04	2.8	0.22	5.4	7.5	8.2	0.06	2.3	0.18	6.0	9.2	8.8	0.07	2.7	0.21	5.1	8.9	8.4	0.07	3.1	0.24	8.1	10.6
0.5	-	6	L	8.3	0.07	2.9	0.23	6.0	8.4	7.4	0.06	2.3	0.18	6.0	7.7	8.1	0.07	3.2	0.25	5.7	7.4	7.4	0.07	3.4	0.27	8.6	8.5
0.5	-	6	L	8.3	0.06	2.9	0.23	6.2	9.2	7.4	0.03	2.3	0.18	6.0	9.5	7.6	0.04	3.9	0.30	5.4	8.4	7.8	0.07	3.7	0.29	8.3	8.8
0.5	-	7.6	L	8.3	0.02	2.7	0.21	6.0	6.9	8.4	0.07	2.7	0.22	6.0	6.8	7.4	0.02	3.4	0.26	6.0	8.3	7.7	0.07	3.6	0.29	8.4	9.0
0.5	-	7.6	L	8.5	0.04	2.7	0.21	6.0	7.7	8.6	0.07	2.4	0.19	6.0	9.3	7.9	0.06	3.4	0.26	6.2	7.2	8.8	0.05	3.9	0.31	8.3	8.6
0.5	-	7.6	L	8.4	0.06	2.6	0.21	6.8	8.4	7.7	0.06	2.8	0.22	6.0	7.8	7.8	0.06	3.4	0.27	6.3	8.6	8.2	0.06	3.3	0.26	9.0	9.2
0.5	-	7.6	L	7.5	0.06	2.9	0.22	6.2	6.9	7.0	0.06	2.7	0.21	5.6	6.9	8.7	0.06	3.1	0.24	6.2	7.8	7.8	0.05	3.8	0.30	7.7	9.0
0.5	-	7.6	L	7.6	0.05	2.9	0.23	6.3	7.5	8.0	0.04	2.7	0.21	6.0	7.4	7.3	0.05	3.6	0.29	5.3	6.8	7.2	0.05	3.3	0.26	6.8	7.5
0.5	-	8.6	L	8.1	0.03	2.9	0.23	6.0	7.2	7.8	0.07	2.8	0.22	6.3	7.7	7.9	0.03	3.1	0.24	6.0	6.8	8.4	0.07	4.2	0.33	8.0	9.1
0.5	-	8.6	L	8.0	0.03	3.0	0.23	6.5	7.7	8.3	0.05	2.8	0.22	6.0	6.9	8.3	0.06	3.7	0.29	6.0	7.1	8.4	0.07	3.9	0.30	8.4	9.4

0.5	-	8.6	L	8.5	0.03	2.9	0.23	6.5	7.2	8.4	0.07	2.5	0.20	6.3	7.8	7.6	0.07	4.0	0.31	6.0	7.1	7.4	0.03	4.1	0.32	9.8	10.4
0.5	-	8.6	L	7.2	0.04	2.9	0.22	6.2	7.2	8.0	0.06	3.1	0.24	6.2	7.2	8.7	0.04	3.5	0.28	6.0	7.4	8.1	0.07	3.9	0.30	6.8	8.4
0.5	-	8.6	L	8.1	0.07	2.9	0.23	6.3	7.5	8.3	0.06	2.5	0.19	6.2	6.9	7.6	0.04	3.8	0.30	6.0	7.8	8.9	0.06	4.4	0.35	7.7	8.0
5	1	6	L	6.8	0.06	3.1	0.24	6.5	9.5	5.7	0.07	3.1	0.24	7.7	7.5	25.4	0.06	3.5	0.28	10.8	22.7	27.8	0.07	3.8	0.30	23.1	27.5
5	2	6	L	6.6	0.07	3.4	0.27	7.5	9.6	5.8	0.07	3.0	0.24	8.1	8.1	25.8	0.06	3.8	0.30	10.5	22.1	27.2	0.06	3.9	0.31	18.3	27.6
5	3	6	L	6.9	0.06	3.5	0.27	7.8	8.4	5.8	0.07	3.4	0.27	6.3	9.6	25.7	0.06	3.9	0.31	9.2	22.5	28.6	0.06	3.3	0.26	30.9	28.1
5	4	6	L	6.8	0.07	2.9	0.22	6.6	8.0	6.2	0.06	3.5	0.27	6.6	7.8	8.9	0.03	3.5	0.28	9.3	22.5	32.4	0.06	3.3	0.26	22.8	28.4
5	5	6	L	6.7	0.07	3.2	0.25	7.5	8.1	5.9	0.07	3.6	0.28	6.2	8.9	24.6	0.04	3.3	0.26	10.1	22.1	27.3	0.06	3.5	0.28	23.4	27.8
5	1	7.6	L	6.2	0.07	2.9	0.23	6.5	8.4	6.2	0.07	3.2	0.25	7.5	7.5	11.5	0.07	3.8	0.29	7.7	15.3	12.2	0.07	3.3	0.26	10.1	14.8
5	2	7.6	L	6.4	0.07	2.8	0.22	6.0	9.2	6.3	0.07	3.3	0.26	6.5	8.3	12.2	0.07	3.8	0.30	8.1	15.7	12.0	0.07	3.5	0.27	11.4	14.4
5	3	7.6	L	6.2	0.06	2.9	0.23	6.5	9.9	6.1	0.06	3.3	0.26	6.5	8.6	11.8	0.06	3.7	0.29	9.5	13.7	12.0	0.06	3.3	0.26	11.1	14.2
5	4	7.6	L	6.3	0.07	2.5	0.20	7.7	7.8	6.4	0.07	3.3	0.26	7.5	8.3	7.1	0.04	3.1	0.24	9.0	15.0	13.3	0.07	2.2	0.17	9.0	13.5
5	5	7.6	L	6.4	0.07	2.6	0.20	7.1	8.0	6.5	0.07	2.1	0.17	6.2	7.5	13.8	0.05	3.0	0.24	9.2	14.9	12.2	0.07	2.2	0.17	10.5	12.7
5	1	8.6	L	6.1	0.05	3.2	0.25	6.2	8.0	5.6	0.07	3.4	0.27	6.0	8.4	8.9	0.07	3.4	0.26	7.7	11.9	6.3	0.07	3.3	0.26	11.0	10.5
5	2	8.6	L	7.1	0.07	4.3	0.34	6.8	8.6	5.7	0.07	3.4	0.27	6.5	7.8	10.1	0.08	3.4	0.27	6.9	13.2	6.3	0.07	3.6	0.29	9.9	11.3
5	3	8.6	L	7.8	0.07	3.2	0.25	6.6	8.9	6.2	0.06	2.7	0.21	6.8	8.0	10.2	0.07	3.4	0.27	9.2	11.6	8.4	0.07	2.7	0.22	9.5	14.4
5	4	8.6	L	7.2	0.07	3.2	0.25	7.7	8.9	6.2	0.06	3.3	0.26	6.0	8.7	6.6	0.04	3.1	0.24	7.8	13.0	8.7	0.07	2.6	0.21	10.4	11.8
5	5	8.6	L	7.5	0.05	2.9	0.23	8.4	7.5	6.1	0.07	2.8	0.22	6.8	8.6	10.3	0.07	3.3	0.26	8.9	12.9	8.6	0.07	2.7	0.21	10.2	13.1
5	-	6	L	6.5	0.05	3.2	0.25	6.6	9.9	6.5	0.06	2.6	0.20	6.5	7.7	8.6	0.04	3.8	0.30	6.0	7.4	5.4	0.07	3.0	0.24	8.9	8.7
5	-	6	L	6.7	0.05	3.3	0.26	6.6	9.8	6.3	0.07	3.0	0.23	6.2	9.9	7.7	0.05	3.6	0.28	6.0	8.1	5.1	0.07	3.3	0.26	8.4	8.8
5	-	6	L	6.9	0.06	3.7	0.29	6.2	8.6	6.6	0.06	2.8	0.22	6.3	8.9	7.5	0.05	3.8	0.30	5.7	9.3	5.4	0.07	3.3	0.26	7.7	8.9
5	-	6	L	6.1	0.07	4.4	0.34	7.8	10.2	8.0	0.07	2.9	0.23	6.3	8.1	6.1	0.06	3.8	0.30	6.3	8.4	8.5	0.07	3.1	0.25	8.6	9.3

5	-	6	L	6.3	0.07	3.7	0.29	8.1	8.1	7.7	0.07	3.6	0.28	6.0	8.0	6.9	0.03	3.2	0.25	6.0	7.5	8.7	0.07	3.6	0.28	8.9	8.2
5	-	7.6	L	5.9	0.06	2.2	0.17	6.5	7.8	6.4	0.07	3.2	0.25	6.3	9.0	5.9	0.07	3.3	0.26	6.0	7.8	6.2	0.07	3.8	0.30	9.3	9.0
5	-	7.6	L	5.5	0.07	3.1	0.24	6.2	7.8	6.6	0.07	3.1	0.24	6.6	9.5	5.9	0.07	3.6	0.28	6.0	8.0	6.4	0.07	3.4	0.27	9.9	9.4
5	-	7.6	L	6.0	0.07	3.1	0.24	6.2	7.4	6.4	0.07	3.2	0.25	6.8	9.0	6.0	0.05	3.8	0.30	6.5	8.6	6.5	0.07	3.6	0.28	8.9	8.7
5	-	7.6	L	5.8	0.07	3.8	0.30	7.4	8.4	6.6	0.07	3.1	0.25	6.5	8.1	6.6	0.07	3.1	0.25	6.6	8.3	7.4	0.06	3.9	0.30	8.1	8.5
5	-	7.6	L	6.0	0.07	3.9	0.31	8.3	8.7	6.0	0.05	2.8	0.22	6.0	9.5	6.8	0.05	3.7	0.29	6.2	11.1	8.3	0.07	3.5	0.28	8.7	8.0
5	-	8.6	L	6.1	0.07	3.9	0.31	6.0	7.7	6.3	0.06	3.7	0.29	6.9	9.2	7.0	0.04	3.5	0.27	6.6	8.4	6.1	0.07	3.6	0.28	9.3	8.9
5	-	8.6	L	5.9	0.06	4.1	0.32	6.0	8.0	7.5	0.05	3.0	0.23	6.5	9.5	6.6	0.07	3.9	0.30	7.5	9.6	5.8	0.06	4.0	0.31	10.7	9.4
5	-	8.6	L	6.0	0.06	3.3	0.26	6.2	8.4	5.3	0.07	3.6	0.28	6.8	9.0	6.3	0.06	3.7	0.29	6.3	9.0	6.7	0.06	3.5	0.27	8.3	8.7
5	-	8.6	L	6.2	0.07	3.2	0.25	6.8	9.6	5.5	0.07	3.7	0.29	6.3	8.6	6.3	0.07	3.6	0.29	6.5	7.7	6.9	0.07	4.1	0.32	8.6	8.1
5	-	8.6	L	6.2	0.07	3.2	0.25	6.2	9.0	5.4	0.06	3.7	0.29	5.4	8.1	5.3	0.07	4.1	0.32	6.0	7.8	6.5	0.07	4.4	0.35	9.2	8.0
