# Supplementary material for: Extracellular Polymeric Substances (EPS) of Freshwater Biofilms Stabilize and Modify CeO2 and Ag Nanoparticles
Source: PLoS One. 2014 Oct 21;9(10):e110709. doi: 10.1371/journal.pone.0110709 (PMC4204993; doi:10.1371/journal.pone.0110709)
Supplement: Table S13 — Z-averages (DLS), polydispersity (PDI), mode and mean diameters (NTA), zetapotential, and EPM of NP formed in AgNO3 solutions dependent on pH, light/dark, EPS content, and time. ns: no signal. Each value is derived from three measurements of three replicates (3x 3n). blue: data used to calculate mean values represented in Figures 8 A–C. (PDF) [file pone.0110709.s021.pdf]

				3 h						24 h						168 h						336 h					
mg/L	EPS	pH	L/D	z-average	PDI	ZP	EPM	Mode	Mean	z-average	PDI	ZP	EPM	Mode	Mean	z-average	PDI	ZP	EPM	Mode	Mean	z-average	PDI	ZP	EPM	Mode	Mean
0.5	1	6	D	ns	ns	ns	ns	ns	ns	ns	ns	ns	ns	ns	ns	ns	ns	ns	ns	ns	ns	ns	ns	ns	ns	ns	ns
0.5	2	6	D	ns	ns	ns	ns	ns	ns	ns	ns	ns	ns	ns	ns	ns	ns	ns	ns	ns	ns	ns	ns	ns	ns	ns	ns
0.5	3	6	D	ns	ns	ns	ns	ns	ns	ns	ns	ns	ns	ns	ns	ns	ns	ns	ns	ns	ns	ns	ns	ns	ns	ns	ns
0.5	4	6	D	ns	ns	ns	ns	ns	ns	ns	ns	ns	ns	ns	ns	ns	ns	ns	ns	ns	ns	ns	ns	ns	ns	ns	ns
0.5	5	6	D	ns	ns	ns	ns	ns	ns	ns	ns	ns	ns	ns	ns	ns	ns	ns	ns	ns	ns	ns	ns	ns	ns	ns	ns
0.5	1	7.6	D	ns	ns	ns	ns	ns	ns	ns	ns	ns	ns	ns	ns	ns	ns	ns	ns	ns	ns	ns	ns	ns	ns	ns	ns
0.5	2	7.6	D	ns	ns	ns	ns	ns	ns	ns	ns	ns	ns	ns	ns	ns	ns	ns	ns	ns	ns	ns	ns	ns	ns	ns	ns
0.5	3	7.6	D	ns	ns	ns	ns	ns	ns	ns	ns	ns	ns	ns	ns	ns	ns	ns	ns	ns	ns	ns	ns	ns	ns	ns	ns
0.5	4	7.6	D	ns	ns	ns	ns	ns	ns	ns	ns	ns	ns	ns	ns	ns	ns	ns	ns	ns	ns	ns	ns	ns	ns	ns	ns
0.5	5	7.6	D	ns	ns	ns	ns	ns	ns	ns	ns	ns	ns	ns	ns	ns	ns	ns	ns	ns	ns	ns	ns	ns	ns	ns	ns
0.5	1	8.6	D	ns	ns	ns	ns	ns	ns	ns	ns	ns	ns	ns	ns	ns	ns	ns	ns	ns	ns	ns	ns	ns	ns	ns	ns
0.5	2	8.6	D	ns	ns	ns	ns	ns	ns	ns	ns	ns	ns	ns	ns	ns	ns	ns	ns	ns	ns	ns	ns	ns	ns	ns	ns
0.5	3	8.6	D	ns	ns	ns	ns	ns	ns	ns	ns	ns	ns	ns	ns	ns	ns	ns	ns	ns	ns	ns	ns	ns	ns	ns	ns
0.5	4	8.6	D	ns	ns	ns	ns	ns	ns	ns	ns	ns	ns	ns	ns	ns	ns	ns	ns	ns	ns	ns	ns	ns	ns	ns	ns
0.5	5	8.6	D	ns	ns	ns	ns	ns	ns	ns	ns	ns	ns	ns	ns	ns	ns	ns	ns	ns	ns	ns	ns	ns	ns	ns	ns
0.5	-	6	D	ns	ns	ns	ns	ns	ns	ns	ns	ns	ns	ns	ns	ns	ns	ns	ns	ns	ns	ns	ns	ns	ns	ns	ns
0.5	-	6	D	ns	ns	ns	ns	ns	ns	ns	ns	ns	ns	ns	ns	ns	ns	ns	ns	ns	ns	ns	ns	ns	ns	ns	ns
0.5	-	6	D	ns	ns	ns	ns	ns	ns	ns	ns	ns	ns	ns	ns	ns	ns	ns	ns	ns	ns	ns	ns	ns	ns	ns	ns
0.5	-	6	D	ns	ns	ns	ns	ns	ns	ns	ns	ns	ns	ns	ns	ns	ns	ns	ns	ns	ns	ns	ns	ns	ns	ns	ns
0.5	-	6	D	ns	ns	ns	ns	ns	ns	ns	ns	ns	ns	ns	ns	ns	ns	ns	ns	ns	ns	ns	ns	ns	ns	ns	ns
0.5	-	7.6	D	ns	ns	ns	ns	ns	ns	ns	ns	ns	ns	ns	ns	ns	ns	ns	ns	ns	ns	ns	ns	ns	ns	ns	ns

0.5	-	7.6	D	ns	ns	ns	ns	ns	ns	ns	ns	ns	ns	ns	ns	ns	ns	ns	ns	ns	ns	ns	ns	ns	ns	ns	ns
0.5	-	7.6	D	ns	ns	ns	ns	ns	ns	ns	ns	ns	ns	ns	ns	ns	ns	ns	ns	ns	ns	ns	ns	ns	ns	ns	ns
0.5	-	7.6	D	ns	ns	ns	ns	ns	ns	ns	ns	ns	ns	ns	ns	ns	ns	ns	ns	ns	ns	ns	ns	ns	ns	ns	ns
0.5	-	7.6	D	ns	ns	ns	ns	ns	ns	ns	ns	ns	ns	ns	ns	ns	ns	ns	ns	ns	ns	ns	ns	ns	ns	ns	ns
0.5	-	8.6	D	ns	ns	ns	ns	ns	ns	ns	ns	ns	ns	ns	ns	ns	ns	ns	ns	ns	ns	ns	ns	ns	ns	ns	ns
0.5	-	8.6	D	ns	ns	ns	ns	ns	ns	ns	ns	ns	ns	ns	ns	ns	ns	ns	ns	ns	ns	ns	ns	ns	ns	ns	ns
0.5	-	8.6	D	ns	ns	ns	ns	ns	ns	ns	ns	ns	ns	ns	ns	ns	ns	ns	ns	ns	ns	ns	ns	ns	ns	ns	ns
0.5	-	8.6	D	ns	ns	ns	ns	ns	ns	ns	ns	ns	ns	ns	ns	ns	ns	ns	ns	ns	ns	ns	ns	ns	ns	ns	ns
0.5	-	8.6	D	ns	ns	ns	ns	ns	ns	ns	ns	ns	ns	ns	ns	ns	ns	ns	ns	ns	ns	ns	ns	ns	ns	ns	ns
5	1	6	D	ns	ns	ns	ns	ns	ns	ns	ns	ns	ns	ns	ns	ns	ns	ns	ns	ns	ns	ns	ns	ns	ns	ns	ns
5	2	6	D	ns	ns	ns	ns	ns	ns	ns	ns	ns	ns	ns	ns	ns	ns	ns	ns	ns	ns	ns	ns	ns	ns	ns	ns
5	3	6	D	ns	ns	ns	ns	ns	ns	ns	ns	ns	ns	ns	ns	ns	ns	ns	ns	ns	ns	ns	ns	ns	ns	ns	ns
5	4	6	D	ns	ns	ns	ns	ns	ns	ns	ns	ns	ns	ns	ns	ns	ns	ns	ns	ns	ns	ns	ns	ns	ns	ns	ns
5	5	6	D	ns	ns	ns	ns	ns	ns	ns	ns	ns	ns	ns	ns	ns	ns	ns	ns	ns	ns	ns	ns	ns	ns	ns	ns
5	1	7.6	D	ns	ns	ns	ns	ns	ns	ns	ns	ns	ns	ns	ns	ns	ns	ns	ns	ns	ns	ns	ns	ns	ns	ns	ns
5	2	7.6	D	ns	ns	ns	ns	ns	ns	ns	ns	ns	ns	ns	ns	ns	ns	ns	ns	ns	ns	ns	ns	ns	ns	ns	ns
5	3	7.6	D	ns	ns	ns	ns	ns	ns	ns	ns	ns	ns	ns	ns	ns	ns	ns	ns	ns	ns	ns	ns	ns	ns	ns	ns
5	4	7.6	D	ns	ns	ns	ns	ns	ns	ns	ns	ns	ns	ns	ns	ns	ns	ns	ns	ns	ns	ns	ns	ns	ns	ns	ns
5	5	7.6	D	ns	ns	ns	ns	ns	ns	ns	ns	ns	ns	ns	ns	ns	ns	ns	ns	ns	ns	ns	ns	ns	ns	ns	ns
5	1	8.6	D	ns	ns	ns	ns	ns	ns	ns	ns	ns	ns	ns	ns	ns	ns	ns	ns	ns	ns	ns	ns	ns	ns	ns	ns
5	2	8.6	D	ns	ns	ns	ns	ns	ns	ns	ns	ns	ns	ns	ns	ns	ns	ns	ns	ns	ns	ns	ns	ns	ns	ns	ns
5	3	8.6	D	ns	ns	ns	ns	ns	ns	ns	ns	ns	ns	ns	ns	ns	ns	ns	ns	ns	ns	ns	ns	ns	ns	ns	ns

5	4	8.6	D	ns	ns	ns	ns	ns	ns	ns	ns	ns	ns	ns	ns	ns	ns	ns	ns	ns	ns	ns	ns	ns	ns	ns	ns
5	5	8.6	D	ns	ns	ns	ns	ns	ns	ns	ns	ns	ns	ns	ns	ns	ns	ns	ns	ns	ns	ns	ns	ns	ns	ns	ns
5	-	6	D	ns	ns	ns	ns	ns	ns	ns	ns	ns	ns	ns	ns	ns	ns	ns	ns	ns	ns	ns	ns	ns	ns	ns	ns
5	-	6	D	ns	ns	ns	ns	ns	ns	ns	ns	ns	ns	ns	ns	ns	ns	ns	ns	ns	ns	ns	ns	ns	ns	ns	ns
5	-	6	D	ns	ns	ns	ns	ns	ns	ns	ns	ns	ns	ns	ns	ns	ns	ns	ns	ns	ns	ns	ns	ns	ns	ns	ns
5	-	6	D	ns	ns	ns	ns	ns	ns	ns	ns	ns	ns	ns	ns	ns	ns	ns	ns	ns	ns	ns	ns	ns	ns	ns	ns
5	-	6	D	ns	ns	ns	ns	ns	ns	ns	ns	ns	ns	ns	ns	ns	ns	ns	ns	ns	ns	ns	ns	ns	ns	ns	ns
5	-	7.6	D	ns	ns	ns	ns	ns	ns	ns	ns	ns	ns	ns	ns	ns	ns	ns	ns	ns	ns	ns	ns	ns	ns	ns	ns
5	-	7.6	D	ns	ns	ns	ns	ns	ns	ns	ns	ns	ns	ns	ns	ns	ns	ns	ns	ns	ns	ns	ns	ns	ns	ns	ns
5	-	7.6	D	ns	ns	ns	ns	ns	ns	ns	ns	ns	ns	ns	ns	ns	ns	ns	ns	ns	ns	ns	ns	ns	ns	ns	ns
5	-	7.6	D	ns	ns	ns	ns	ns	ns	ns	ns	ns	ns	ns	ns	ns	ns	ns	ns	ns	ns	ns	ns	ns	ns	ns	ns
5	-	7.6	D	ns	ns	ns	ns	ns	ns	ns	ns	ns	ns	ns	ns	ns	ns	ns	ns	ns	ns	ns	ns	ns	ns	ns	ns
5	-	8.6	D	ns	ns	ns	ns	ns	ns	ns	ns	ns	ns	ns	ns	ns	ns	ns	ns	ns	ns	ns	ns	ns	ns	ns	ns
5	-	8.6	D	ns	ns	ns	ns	ns	ns	ns	ns	ns	ns	ns	ns	ns	ns	ns	ns	ns	ns	ns	ns	ns	ns	ns	ns
5	-	8.6	D	ns	ns	ns	ns	ns	ns	ns	ns	ns	ns	ns	ns	ns	ns	ns	ns	ns	ns	ns	ns	ns	ns	ns	ns
5	-	8.6	D	ns	ns	ns	ns	ns	ns	ns	ns	ns	ns	ns	ns	ns	ns	ns	ns	ns	ns	ns	ns	ns	ns	ns	ns
5	-	8.6	D	ns	ns	ns	ns	ns	ns	ns	ns	ns	ns	ns	ns	ns	ns	ns	ns	ns	ns	ns	ns	ns	ns	ns	ns
0.5	1	6	L	120	0.466	-17.5	-1.37	115	163	159	0.388	-19.2	-1.51	106	142	176	0.36	-19.1	-1.50	86	138	160	0.619	-19.9	-1.56	90	142
0.5	2	6	L	112	0.312	-21.1	-1.66	82	162	166	0.436	-22.6	-1.77	127	154	187	0.315	-20.7	-1.62	109	150	135	0.604	-19.0	-1.49	121	163
0.5	3	6	L	97	0.397	-17.5	-1.37	93	166	155	0.445	-19.3	-1.51	168	154	190	0.405	-19.6	-1.54	131	149	135	0.56	-19.1	-1.50	121	126
0.5	4	6	L	ns	ns	ns	ns	ns	ns	89	0.464	-17.8	-1.40	107	133	161	0.455	-20.9	-1.64	173	183	138	0.401	-19.3	-1.51	122	124
0.5	5	6	L	106	0.354	-18.4	-1.44	115	139	159	0.403	-20.9	-1.64	127	151	145	0.298	-22.5	-1.77	116	150	135	0.427	-21.2	-1.66	104	111

0.5	1	7.6	L	155	0.491	-20.0	-1.57	126	146	102	0.46	-15.0	-1.18	113	139	145	0.385	-21.5	-1.69	115	149	133	0.429	-19.1	-1.49	139	154
0.5	2	7.6	L	136	0.383	-19.4	-1.52	132	147	99	0.41	-20.5	-1.61	125	136	152	0.345	-22.5	-1.76	133	164	124	0.59	-19.5	-1.53	95	150
0.5	3	7.6	L	165	0.404	-19.9	-1.56	127	151	118	0.484	-19.1	-1.50	111	150	141	0.357	-24.3	-1.90	92	149	123	0.577	-21.0	-1.65	82	137
0.5	4	7.6	L	ns	ns	ns	ns	ns	ns	168	0.416	-19.3	-1.51	102	151	128	0.396	-18.6	-1.46	104	143	107	0.608	-22.4	-1.76	114	133
0.5	5	7.6	L	172	0.423	-19.3	-1.51	90	134	134	0.508	-22.1	-1.73	115	153	141	0.343	-25.3	-1.98	114	147	132	0.569	-21.6	-1.69	88	126
0.5	1	8.6	L	112	0.449	-18.5	-1.45	101	154	133	0.512	-19.6	-1.54	92	160	142	0.341	-26.2	-2.06	88	144	104	0.529	-16.8	-1.31	121	172
0.5	2	8.6	L	115	0.452	-18.5	-1.45	151	168	136	0.465	-18.0	-1.41	116	161	118	0.416	-20.7	-1.63	82	125	104	0.417	-19.5	-1.53	138	170
0.5	3	8.6	L	115	0.446	-18.8	-1.48	124	167	175	0.438	-17.8	-1.39	136	157	131	0.593	-26.8	-2.10	119	162	110	0.519	-17.7	-1.39	131	176
0.5	4	8.6	L	ns	ns	ns	ns	ns	ns	173	0.473	-15.7	-1.23	124	168	126	0.741	-21.7	-1.70	105	162	131	0.593	-26.8	-2.10	100	136
0.5	5	8.6	L	183	0.437	-16.7	-1.31	172	181	172	0.536	-19.4	-1.52	163	146	133	0.576	-24.6	-1.93	155	165	126	0.741	-21.7	-1.70	102	123
0.5	-	6	L	ns	ns	ns	ns	ns	ns	ns	ns	ns	ns	ns	ns	ns	ns	ns	ns	ns	ns	ns	ns	ns	ns	ns	ns
0.5	-	6	L	ns	ns	ns	ns	ns	ns	ns	ns	ns	ns	ns	ns	ns	ns	ns	ns	ns	ns	ns	ns	ns	ns	ns	ns
0.5	-	6	L	ns	ns	ns	ns	ns	ns	ns	ns	ns	ns	ns	ns	ns	ns	ns	ns	ns	ns	ns	ns	ns	ns	ns	ns
0.5	-	6	L	ns	ns	ns	ns	ns	ns	ns	ns	ns	ns	ns	ns	ns	ns	ns	ns	ns	ns	ns	ns	ns	ns	ns	ns
0.5	-	6	L	ns	ns	ns	ns	ns	ns	ns	ns	ns	ns	ns	ns	ns	ns	ns	ns	ns	ns	ns	ns	ns	ns	ns	ns
0.5	-	7.6	L	ns	ns	ns	ns	ns	ns	ns	ns	ns	ns	ns	ns	ns	ns	ns	ns	ns	ns	ns	ns	ns	ns	ns	ns
0.5	-	7.6	L	ns	ns	ns	ns	ns	ns	ns	ns	ns	ns	ns	ns	ns	ns	ns	ns	ns	ns	ns	ns	ns	ns	ns	ns
0.5	-	7.6	L	ns	ns	ns	ns	ns	ns	ns	ns	ns	ns	ns	ns	ns	ns	ns	ns	ns	ns	ns	ns	ns	ns	ns	ns
0.5	-	7.6	L	ns	ns	ns	ns	ns	ns	ns	ns	ns	ns	ns	ns	ns	ns	ns	ns	ns	ns	ns	ns	ns	ns	ns	ns
0.5	-	7.6	L	ns	ns	ns	ns	ns	ns	ns	ns	ns	ns	ns	ns	ns	ns	ns	ns	ns	ns	ns	ns	ns	ns	ns	ns
0.5	-	8.6	L	ns	ns	ns	ns	ns	ns	ns	ns	ns	ns	ns	ns	ns	ns	ns	ns	ns	ns	ns	ns	ns	ns	ns	ns
0.5	-	8.6	L	ns	ns	ns	ns	ns	ns	ns	ns	ns	ns	ns	ns	ns	ns	ns	ns	ns	ns	ns	ns	ns	ns	ns	ns

0.5	-	8.6	L	ns	ns	ns	ns	ns	ns	ns	ns	ns	ns	ns	ns	ns	ns	ns	ns	ns	ns	ns	ns	ns	ns	ns	ns
0.5	-	8.6	L	ns	ns	ns	ns	ns	ns	ns	ns	ns	ns	ns	ns	ns	ns	ns	ns	ns	ns	ns	ns	ns	ns	ns	ns
0.5	-	8.6	L	ns	ns	ns	ns	ns	ns	ns	ns	ns	ns	ns	ns	ns	ns	ns	ns	ns	ns	ns	ns	ns	ns	ns	ns
5	1	6	L	89	0.421	-16.9	-1.33	101	119	131	0.44	-18.6	-1.46	106	141	157	0.485	-18.8	-1.48	104	127	152	0.273	-23.0	-1.81	89	138
5	2	6	L	90	0.422	-17.6	-1.38	90	113	131	0.444	-18.0	-1.41	97	130	161	0.427	-21.4	-1.68	94	132	142	0.27	-23.7	-1.86	97	151
5	3	6	L	91	0.426	-18.7	-1.47	75	116	130	0.444	-20.3	-1.59	103	154	155	0.442	-20.0	-1.57	85	128	152	0.26	-23.9	-1.88	111	159
5	4	6	L	ns	ns	ns	ns	ns	ns	110	0.403	-14.1	-1.10	90	153	149	0.475	-20.6	-1.62	80	126	178	0.429	-18.4	-1.44	86	148
5	5	6	L	114	0.434	-15.7	-1.23	101	131	147	0.455	-20.0	-1.57	111	140	146	0.379	-21.1	-1.66	77	154	150	0.419	-18.6	-1.46	92	159
5	1	7.6	L	87	0.375	-19.8	-1.55	103	113	144	0.462	-19.6	-1.53	115	125	147	0.379	-23.0	-1.80	147	166	164	0.445	-19.1	-1.50	90	153
5	2	7.6	L	86	0.379	-17.8	-1.40	98	117	146	0.442	-22.6	-1.77	92	133	148	0.352	-22.2	-1.74	97	128	146	0.264	-23.5	-1.84	76	150
5	3	7.6	L	88	0.376	-14.6	-1.14	104	105	145	0.444	-22.4	-1.75	137	139	143	0.452	-18.3	-1.43	97	123	151	0.269	-24.3	-1.91	96	172
5	4	7.6	L	ns	ns	ns	ns	ns	ns	174	0.393	-17.1	-1.34	108	153	140	0.419	-23.3	-1.83	90	124	146	0.261	-24.4	-1.91	93	129
5	5	7.6	L	172	0.426	-18.5	-1.45	107	173	136	0.458	-21.7	-1.70	112	171	141	0.449	-20.5	-1.61	82	128	160	0.411	-11.9	-0.93	86	120
5	1	8.6	L	94	0.37	-20.2	-1.58	97	130	142	0.467	-22.6	-1.77	111	140	142	0.454	-18.2	-1.42	103	132	159	0.42	-13.0	-1.02	86	118
5	2	8.6	L	95	0.387	-19.0	-1.49	80	127	138	0.449	-20.9	-1.64	112	128	122	0.255	-21.1	-1.65	74	126	170	0.537	-14.8	-1.16	84	125
5	3	8.6	L	161	0.46	-19.0	-1.49	159	150	150	0.457	-19.4	-1.52	93	118	123	0.252	-22.7	-1.78	128	154	131	0.645	-35.9	-2.82	92	167
5	4	8.6	L	ns	ns	ns	ns	ns	ns	160	0.42	-19.4	-1.52	118	159	146	0.442	-22.0	-1.72	184	208	139	0.327	-36.0	-2.82	87	128
5	5	8.6	L	152	0.404	-19.8	-1.55	102	158	146	0.442	-20.9	-1.64	116	180	123	0.257	-23.0	-1.80	120	192	139	0.209	-35.7	-2.80	91	128
5	-	6	L	ns	ns	ns	ns	ns	ns	ns	ns	ns	ns	ns	ns	ns	ns	ns	ns	ns	ns	ns	ns	ns	ns	ns	ns
5	-	6	L	ns	ns	ns	ns	ns	ns	ns	ns	ns	ns	ns	ns	ns	ns	ns	ns	ns	ns	ns	ns	ns	ns	ns	ns
5	-	6	L	ns	ns	ns	ns	ns	ns	ns	ns	ns	ns	ns	ns	ns	ns	ns	ns	ns	ns	ns	ns	ns	ns	ns	ns
5	-	6	L	ns	ns	ns	ns	ns	ns	ns	ns	ns	ns	ns	ns	ns	ns	ns	ns	ns	ns	ns	ns	ns	ns	ns	ns

5	-	6	L	ns	ns	ns	ns	ns	ns	ns	ns	ns	ns	ns	ns	ns	ns	ns	ns	ns	ns	ns	ns	ns	ns	ns	ns
5	-	7.6	L	ns	ns	ns	ns	ns	ns	ns	ns	ns	ns	ns	ns	ns	ns	ns	ns	ns	ns	ns	ns	ns	ns	ns	ns
5	-	7.6	L	ns	ns	ns	ns	ns	ns	ns	ns	ns	ns	ns	ns	ns	ns	ns	ns	ns	ns	ns	ns	ns	ns	ns	ns
5	-	7.6	L	ns	ns	ns	ns	ns	ns	ns	ns	ns	ns	ns	ns	ns	ns	ns	ns	ns	ns	ns	ns	ns	ns	ns	ns
5	-	7.6	L	ns	ns	ns	ns	ns	ns	ns	ns	ns	ns	ns	ns	ns	ns	ns	ns	ns	ns	ns	ns	ns	ns	ns	ns
5	-	7.6	L	ns	ns	ns	ns	ns	ns	ns	ns	ns	ns	ns	ns	ns	ns	ns	ns	ns	ns	ns	ns	ns	ns	ns	ns
5	-	8.6	L	ns	ns	ns	ns	ns	ns	ns	ns	ns	ns	ns	ns	ns	ns	ns	ns	ns	ns	ns	ns	ns	ns	ns	ns
5	-	8.6	L	ns	ns	ns	ns	ns	ns	ns	ns	ns	ns	ns	ns	ns	ns	ns	ns	ns	ns	ns	ns	ns	ns	ns	ns
5	-	8.6	L	ns	ns	ns	ns	ns	ns	ns	ns	ns	ns	ns	ns	ns	ns	ns	ns	ns	ns	ns	ns	ns	ns	ns	ns
5	-	8.6	L	ns	ns	ns	ns	ns	ns	ns	ns	ns	ns	ns	ns	ns	ns	ns	ns	ns	ns	ns	ns	ns	ns	ns	ns
5	-	8.6	L	ns	ns	ns	ns	ns	ns	ns	ns	ns	ns	ns	ns	ns	ns	ns	ns	ns	ns	ns	ns	ns	ns	ns	ns
