# Supplementary material for: Extracellular Polymeric Substances (EPS) of Freshwater Biofilms Stabilize and Modify CeO2 and Ag Nanoparticles
Source: PLoS One. 2014 Oct 21;9(10):e110709. doi: 10.1371/journal.pone.0110709 (PMC4204993; doi:10.1371/journal.pone.0110709)
Supplement: Table S14 — Standard deviations of Z-averages (DLS), polydispersity (PDI), mode and mean diameters (NTA), zetapotential, and EPM of NP formed in AgNO3 solutions dependent on pH, light/dark, EPS content, and time. (PDF) [file pone.0110709.s022.pdf]

				3 h						24 h						168 h						336 h					
mg/L	EPS	pH	L/D	z-average	PDI	ZP	EPM	Mode	Mean	z-average	PDI	ZP	EPM	Mode	Mean	z-average	PDI	ZP	EPM	Mode	Mean	z-average	PDI	ZP	EPM	Mode	Mean
0.5	1	6	L	18.0	0.07	2.6	0.21	17.3	24.5	23.8	0.06	2.9	0.23	15.9	21.3	26.4	0.05	2.9	0.22	12.9	20.7	23.9	0.09	3.0	0.23	13.5	21.3
0.5	2	6	L	16.8	0.05	3.2	0.25	12.3	24.3	24.9	0.07	3.4	0.27	19.1	23.1	28.1	0.05	3.1	0.24	16.4	22.5	20.3	0.09	2.9	0.22	18.2	24.5
0.5	3	6	L	14.6	0.06	2.6	0.21	14.0	24.9	23.3	0.07	2.9	0.23	25.2	23.1	28.5	0.06	2.9	0.23	19.7	22.4	20.2	0.08	2.9	0.22	18.2	18.9
0.5	4	6	L							13.4	0.07	2.7	0.21	16.1	20.0	24.2	0.07	3.1	0.25	26.0	27.5	20.7	0.06	2.9	0.23	18.3	18.6
0.5	5	6	L	15.9	0.05	2.8	0.22	17.3	20.9	23.8	0.06	3.1	0.25	19.1	22.7	21.8	0.04	3.4	0.26	17.4	22.5	20.2	0.06	3.2	0.25	15.6	16.7
0.5	1	7.6	L	23.3	0.07	3.0	0.23	18.9	21.9	15.3	0.07	2.3	0.18	17.0	20.9	21.7	0.06	3.2	0.25	17.3	22.4	20.0	0.06	2.9	0.22	20.9	23.1
0.5	2	7.6	L	20.4	0.06	2.9	0.23	19.8	22.1	14.9	0.06	3.1	0.24	18.8	20.4	22.7	0.05	3.4	0.26	20.0	24.6	18.6	0.09	2.9	0.23	14.3	22.5
0.5	3	7.6	L	24.8	0.06	3.0	0.23	19.1	22.7	17.6	0.07	2.9	0.22	16.7	22.5	21.1	0.05	3.6	0.29	13.8	22.4	18.5	0.09	3.2	0.25	12.3	20.6
0.5	4	7.6	L							25.2	0.06	2.9	0.23	15.3	22.7	19.2	0.06	2.8	0.22	15.6	21.5	16.1	0.09	3.4	0.26	17.1	20.0
0.5	5	7.6	L	25.8	0.06	2.9	0.23	13.5	20.1	20.1	0.08	3.3	0.26	17.3	23.0	21.2	0.05	3.8	0.30	17.1	22.1	19.8	0.09	3.2	0.25	13.2	18.9
0.5	1	8.6	L	16.8	0.07	2.8	0.22	15.2	23.1	19.9	0.08	2.9	0.23	13.8	24.0	21.3	0.05	3.9	0.31	13.2	21.6	15.5	0.08	2.5	0.20	18.2	25.8
0.5	2	8.6	L	17.3	0.07	2.8	0.22	22.7	25.2	20.3	0.07	2.7	0.21	17.4	24.2	17.7	0.06	3.1	0.24	12.3	18.8	15.6	0.06	2.9	0.23	20.7	25.5
0.5	3	8.6	L	17.2	0.07	2.8	0.22	18.6	25.1	26.2	0.07	2.7	0.21	20.4	23.6	19.6	0.09	4.0	0.31	17.9	24.3	16.5	0.08	2.7	0.21	19.7	26.4
0.5	4	8.6	L							25.9	0.07	2.4	0.18	18.6	25.2	18.9	0.11	3.3	0.26	15.8	24.3	19.6	0.09	4.0	0.31	15.0	20.4
0.5	5	8.6	L	27.4	0.07	2.5	0.20	25.8	27.2	25.7	0.08	2.9	0.23	24.5	21.9	19.9	0.09	3.7	0.29	23.3	24.8	18.9	0.11	3.3	0.26	15.3	18.5
5	1	6	L	13.4	0.06	2.5	0.20	15.2	17.9	19.6	0.07	2.8	0.22	15.9	21.2	23.5	0.07	2.8	0.22	15.6	19.1	16.8	0.04	3.5	0.27	13.4	20.7
5	2	6	L	13.5	0.06	2.6	0.21	13.5	17.0	19.7	0.07	2.7	0.21	14.6	19.5	24.1	0.06	3.2	0.25	14.1	19.8	16.8	0.04	3.6	0.28	14.6	22.6
5	3	6	L	13.7	0.06	2.8	0.22	11.3	17.4	19.5	0.07	3.0	0.24	15.5	23.1	23.2	0.07	3.0	0.24	12.8	19.2	16.8	0.04	3.6	0.28	16.7	23.8
5	4	6	L							16.5	0.06	2.1	0.17	13.5	23.0	22.4	0.07	3.1	0.24	12.0	18.9	26.6	0.06	2.8	0.22	12.9	22.2
5	5	6	L	17.0	0.07	2.4	0.18	15.2	19.7	22.0	0.07	3.0	0.24	16.7	21.0	21.9	0.06	3.2	0.25	11.6	23.1	28.5	0.06	2.8	0.22	13.8	23.9
5	1	7.6	L	13.1	0.06	3.0	0.23	15.5	17.0	21.5	0.07	2.9	0.23	17.3	18.8	22.1	0.06	3.5	0.27	22.1	24.9	27.5	0.07	2.9	0.22	13.5	22.9

5	2	7.6	L	12.9	0.06	2.7	0.21	14.7	17.6	21.8	0.07	3.4	0.27	13.8	20.0	22.1	0.05	3.3	0.26	14.6	19.2	21.9	0.04	3.5	0.28	11.4	22.5
5	3	7.6	L	13.2	0.06	2.2	0.17	15.6	15.8	21.8	0.07	3.4	0.26	20.6	20.9	21.4	0.07	2.7	0.21	14.6	18.5	22.7	0.04	3.6	0.29	14.4	25.7
5	4	7.6	L							26.1	0.06	2.6	0.20	16.2	23.0	21.0	0.06	3.5	0.27	13.5	18.6	21.8	0.04	3.7	0.29	14.0	19.3
5	5	7.6	L	25.8	0.06	2.8	0.22	16.1	26.0	20.4	0.07	3.3	0.26	16.8	25.7	21.2	0.07	3.1	0.24	12.3	19.2	24.0	0.06	1.8	0.14	12.9	18.0
5	1	8.6	L	14.1	0.06	3.0	0.24	14.6	19.5	21.2	0.07	3.4	0.27	16.7	21.0	21.3	0.07	2.7	0.21	15.5	19.8	23.9	0.06	2.0	0.15	12.9	17.7
5	2	8.6	L	14.3	0.06	2.9	0.22	12.0	19.1	20.7	0.07	3.1	0.25	16.8	19.2	18.2	0.04	3.2	0.25	11.1	18.9	25.6	0.08	2.2	0.17	12.6	18.8
5	3	8.6	L	24.1	0.07	2.9	0.22	23.9	22.5	22.4	0.07	2.9	0.23	14.0	17.7	18.5	0.04	3.4	0.27	19.2	23.1	19.6	0.10	5.4	0.42	13.8	25.1
5	4	8.6	L							24.0	0.06	2.9	0.23	17.7	23.9	21.9	0.07	3.3	0.26	27.6	31.2	20.9	0.05	5.4	0.42	13.1	19.2
5	5	8.6	L	22.9	0.06	3.0	0.23	15.3	23.7	21.9	0.07	3.1	0.25	17.4	27.0	18.5	0.04	3.5	0.27	18.0	28.8	16.4	0.03	5.4	0.42	13.7	19.2
