# Supplementary material for: Extracellular Polymeric Substances (EPS) of Freshwater Biofilms Stabilize and Modify CeO2 and Ag Nanoparticles
Source: PLoS One. 2014 Oct 21;9(10):e110709. doi: 10.1371/journal.pone.0110709 (PMC4204993; doi:10.1371/journal.pone.0110709)
Supplement: Text S1 — Settings for DLS and NTA measurements. (PDF) [file pone.0110709.s023.pdf]

Settings for DLS Measurements

DLS size measurements were performed with a Zetasizer Nano ZS (Malvern Instruments) in DTS1060C cells at 25°C. Refractive indices were 1.828 for Ce and 0.45 for Ag, the dispersant was set to water. Further settings were: 173°C backscatter, automatic measurement duration, 3 measurements per sample, automatic attenuation, and automatic optimum measurement position. Data was processed in general purpose mode. Settings for electrophoretic mobility/zetapotential measurements were automatic attenuation and voltage, a minimum of 10 runs, and a maximum of 30 runs.

Settings for NTA Measurements

Nanoparticle tracking analysis (NTA, NanoSight LM10 equipped with a LM14 temperature controller (NanoSight Ltd.)) was used to determine a number based particle size distribution. Settings were as follows:

Background Extract: On, Brightness: 0, Gain: 1, Blur Size: 9x9, Detection Threshold Type: Single, Detection Threshold: 15, Min track length 10, Min Expected Size: Auto, Temperature: 23 °C, Viscosity: 0.9326.
